# Supplementary material for: Hydroxylation of Progesterone and Its Derivatives by the Entomopathogenic Strain Isaria farinosa KCh KW1.1
Source: Int J Mol Sci. 2022 Jun 24;23(13):7015. doi: 10.3390/ijms23137015 (PMC9266320; doi:10.3390/ijms23137015)
Supplement: Supplementary file 1 [file ijms-23-07015-s001.zip › ijms-1745919-supplementary.pdf]

Supplementary Material

Hydroxylation of progesterone and its derivatives by entomopathogenic strain *Isaria farinosa* KCh KW1.1

Ewa Kozłowska*, Jordan Sycz, Tomasz Janeczko*

Department of Chemistry, Wrocław University of Environmental and Life Sciences, Norwida 25, 50-375 Wrocław, Poland
*e.a.kozlowska@gmail.com (E.K.), janeczko13@interia.pl (T.J.)

Contents:

Figure.S1. ^{1}H NMR spectral of 6β,11α-dihydroxyprogesterone (**2**) ($CDCl_3$, 600 MHz)

Figure.S2. ^{13}C NMR spectral of 6β,11α-dihydroxyprogesterone (**2**) ($CDCl_3$, 151 MHz)

Figure.S3. COSY spectral of 6β,11α-dihydroxyprogesterone (**2**) ($CDCl_3$, 600 MHz)

Figure.S4. HSQC spectral of 6β,11α-dihydroxyprogesterone (**2**) ($CDCl_3$, 151 MHz)

Figure.S5. HMBC spectral of 6β,11α-dihydroxyprogesterone (**2**) ($CDCl_3$, 151 MHz)

Figure.S6. ^{1}H NMR spectral of 6β,11α-dihydroxyprogesterone (**2**) (DMSO-d_6, 600 MHz)

Figure.S7. ^{13}C NMR spectral of 6β,11α-dihydroxyprogesterone (**2**) (DMSO-d_6, 600 MHz)

Figure.S8. COSY spectral of 6β,11α-dihydroxyprogesterone (**2**) (DMSO-d_6, 600 MHz)

Figure.S9. HSQC spectral of 6β,11α-dihydroxyprogesterone (**2**) (DMSO-d_6, 600 MHz)

Figure.S10. HMBC spectral of 6β,11α-dihydroxyprogesterone (**2**) (DMSO-d_6, 600 MHz)

Figure.S11. ^{1}H NMR spectral of 12β,17α-dihydroxyprogesterone (**9**) ($CDCl_3$, 600 MHz)

Figure.S12. ^{13}C NMR spectral of 12β,17α-dihydroxyprogesterone (**9**) ($CDCl_3$, 151 MHz)

Figure.S13. COSY spectral of 12β,17α-dihydroxyprogesterone (**9**) ($CDCl_3$, 600 MHz)

Figure.S14. HSQC spectral of 12β,17α-dihydroxyprogesterone (**9**) ($CDCl_3$, 151 MHz)

Figure.S15. HMBC spectral of 12β,17α-dihydroxyprogesterone (**9**) ($CDCl_3$, 151 MHz)

Figure.S16. ^{1}H NMR spectral of 6β,12β,17α-trihydroxyprogesterone (**10**) ($CDCl_3$, 600 MHz)

Figure.S17. ^{13}C NMR spectral of 6β,12β,17α-trihydroxyprogesterone (**10**) ($CDCl_3$, 151 MHz)

Figure.S18. COSY spectral of 6β,12β,17α-trihydroxyprogesterone (**10**) ($CDCl_3$, 600 MHz)

Figure.S19. HSQC spectral of 6β,12β,17α-trihydroxyprogesterone (**10**) ($CDCl_3$, 151 MHz)

Figure.S20. HMBC spectral of 6β,12β,17α-trihydroxyprogesterone (**10**) ($CDCl_3$, 151 MHz)

Figure.S21. ^{1}H NMR spectral of 6β,11α-dihydroxy-16α,17α-epoxyprogesterone (**12**) ($CDCl_3$, 600 MHz)

Figure.S22. ^{13}C NMR spectral of 6β,11α-dihydroxy-16α,17α-epoxyprogesterone (**12**) ($CDCl_3$, 151 MHz)

Figure.S23. COSY spectral of 6β,11α-dihydroxy-16α,17α-epoxyprogesterone (**12**) ($CDCl_3$, 600 MHz)

Figure.S24. HSQC spectral of 6β,11α-dihydroxy-16α,17α-epoxyprogesterone (**12**) ($CDCl_3$, 151 MHz)

Figure.S25. ^{1}H NMR spectral of 6β,11α-dihydroxy-16α,17α-epoxyprogesterone (**12**) (DMSO-d_6, 600 MHz)

Figure.S26. ^{13}C NMR spectral of 6β,11α-dihydroxy-16α,17α-epoxyprogesterone (**12**) (DMSO-d_6, 600 MHz)

Figure.S27. COSY spectral of 6β,11α-dihydroxy-16α,17α-epoxyprogesterone (**12**) (DMSO-d_6, 600 MHz)

Figure.S28. HSQC spectral of 6β,11α-dihydroxy-16α,17α-epoxyprogesterone (**12**) (DMSO-d_6, 600 MHz)

Figure.S29. HMBC spectral of 6β,11α-dihydroxy-16α,17α-epoxyprogesterone (**12**) (DMSO-d_6, 600 MHz)

Figure.S30. ^{1}H NMR spectral of 11α-hydroxy-7-oxopregnenolone (**13**) ($CDCl_3$, 600 MHz)

Figure.S31. ^{13}C NMR spectral of 11α-hydroxy-7-oxopregnenolone (**13**) ($CDCl_3$, 151 MHz)

Figure.S32. COSY spectral of 11α-hydroxy-7-oxopregnenolone (**13**) ($CDCl_3$, 600 MHz)

Figure.S33. HSQC spectral of 11α-hydroxy-7-oxopregnenolone (**13**) ($CDCl_3$, 151 MHz)

Figure.S34. HMBC spectral of 11α-hydroxy-7-oxopregnenolone (**13**) ($CDCl_3$, 151 MHz)

Figure.S35. ^{1}H NMR spectral of 5α,6α-epoxy-3β,11α-dihydroxypregnan-7,20-dione (**14**) ($CDCl_3$, 600 MHz)

Figure.S36. ^{13}C NMR spectral of 5α,6α-epoxy-3β,11α-dihydroxypregnan-7,20-dione (**14**) ($CDCl_3$, 151 MHz)

Figure.S37. COSY spectral of 5α,6α-epoxy-3β,11α-dihydroxypregnan-7,20-dione (**14**) ($CDCl_3$, 600 MHz)

Figure.S38. HSQC spectral of 5α,6α-epoxy-3β,11α-dihydroxypregnan-7,20-dione (**14**) ($CDCl_3$, 151 MHz) Figure.S39. HMBC spectral of 5α,6α-epoxy-3β,11α-dihydroxypregnan-7,20-dione (**14**) ($CDCl_3$, 151 MHz)

Figure.S40. Thin layer chromatography showing the products of biotransformation 17α-hydroxyprogesterone (**4**): A) one-day transformation; B) three-day transformation; C) seven-day transformation; D) ten-day transformation; E) 6β,12β,17α-trihydroxyprogesterone (**10**); F) 12β,17α-dihydroxyprogesterone (**9**); G) 6β,17α-dihydroxyprogesterone (**8**); H) 17α-hydroxyprogesterone (**4**)

Figure.S41. Thin layer chromatography showing the products of biotransformation 17α-hydroxyprogesterone (**4**): A) one-day transformation; B) three-day transformation; C) seven-day transformation; D) ten-day transformation; E) 6β,12β,17α-trihydroxyprogesterone (**10**); F) 12β,17α-dihydroxyprogesterone (**9**); G) 6β,17α-dihydroxyprogesterone (**8**); H) 17α-hydroxyprogesterone (**4**)

Figure.S42. Thin layer chromatography showing the products of biotransformation 17α-hydroxyprogesterone (**4**): A) one-day transformation; B) three-day transformation; C) seven-day transformation; D) ten-day transformation; E) 6β,12β,17α-trihydroxyprogesterone (**10**); F) 12β,17α-dihydroxyprogesterone (**9**); G) 6β,17α-dihydroxyprogesterone (**8**); H) 17α-hydroxyprogesterone (**4**)

Figure.S43. Thin layer chromatography showing the products of biotransformation 16α,17α-epoxyprogesterone (**5**): A) one-day transformation; B) three-day transformation; C) 16α,17α-epoxyprogesterone (**5**); D) seven-day transformation; E) ten-day transformation

Figure.S44. Thin layer chromatography showing the products of biotransformation 16α,17α-epoxyprogesterone (**5**): A) one-day transformation; B) three-day transformation; C) 16α,17α-epoxyprogesterone (**5**); D) seven-day transformation; E) ten-day transformation

Figure.S45. Progesterone (**1**) physicochemical and ADME parameters prediction using the SwissADME modelling

Figure.S46. 6β,11α-Dihydroxyprogesterone (**2**) physicochemical and ADME parameters prediction using the SwissADME modelling

Figure.S47. 6β-hydroxy-11-oxo-progesterone (**7**) physicochemical and ADME parameters prediction using the SwissADME modelling

Figure.S48. 11α-Hydroxyprogesterone (**3**) physicochemical and ADME parameters prediction using the SwissADME modelling

Figure.S49. 17α-Hydroxyprogesterone (**3**) physicochemical and ADME parameters prediction using the SwissADME modelling

Figure.S50. 6β,17α-dihydroxyprogesterone (**8**) physicochemical and ADME parameters prediction using the SwissADME modelling

Figure.S51. 12β,17α-dihydroxyprogesterone (**8**) physicochemical and ADME parameters prediction using the SwissADME modelling

Figure.S52. 6β,12β,17α-trihydroxyprogesterone (**10**) physicochemical and ADME parameters prediction using the SwissADME modelling

Figure.S53. 16α,17α-epoxyprogesterone (**5**) physicochemical and ADME parameters prediction using the SwissADME modelling

Figure.S54. 6β-hydroxy-16α,17α-epoxyprogesterone (**11**) physicochemical and ADME parameters prediction using the SwissADME modelling

Figure.S55. 6β,11α-dihydroxy-16α,17α-epoxyprogesterone (**12**) physicochemical and ADME parameters prediction using the SwissADME modelling

Figure.S56. Pregnenolone (**6**) physicochemical and ADME parameters prediction using the SwissADME modelling

Figure.S57. 11α-Hydroxy-7-oxopregnenolone (**13**) physicochemical and ADME parameters prediction using the SwissADME modelling

Figure.S58. 5α,6α-epoxy-3β,11α-dihydroxypregnan-7,20-dione (**14**) physicochemical and ADME parameters prediction using the SwissADME modelling

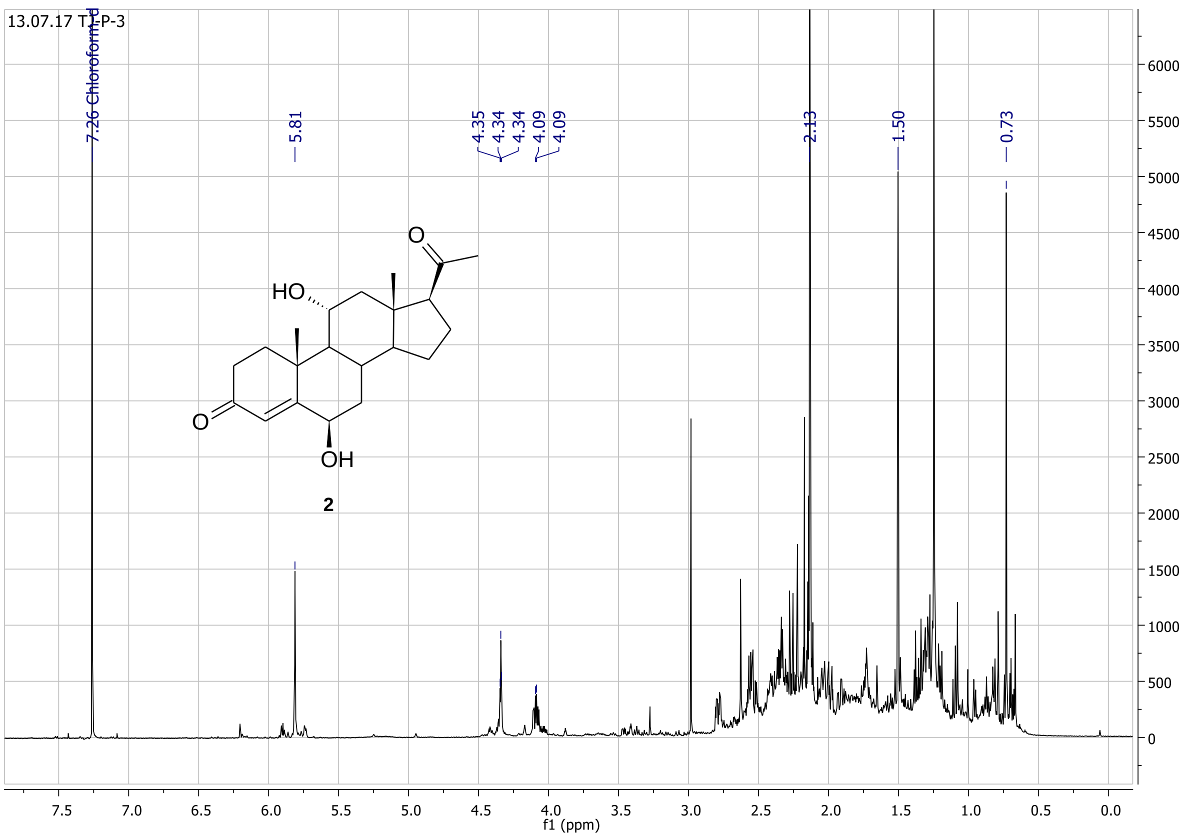

Figure.S1. ^{1}H NMR spectral of 6β,11α-dihydroxyprogesterone (**2**) ($CDCl_3$, 600 MHz)

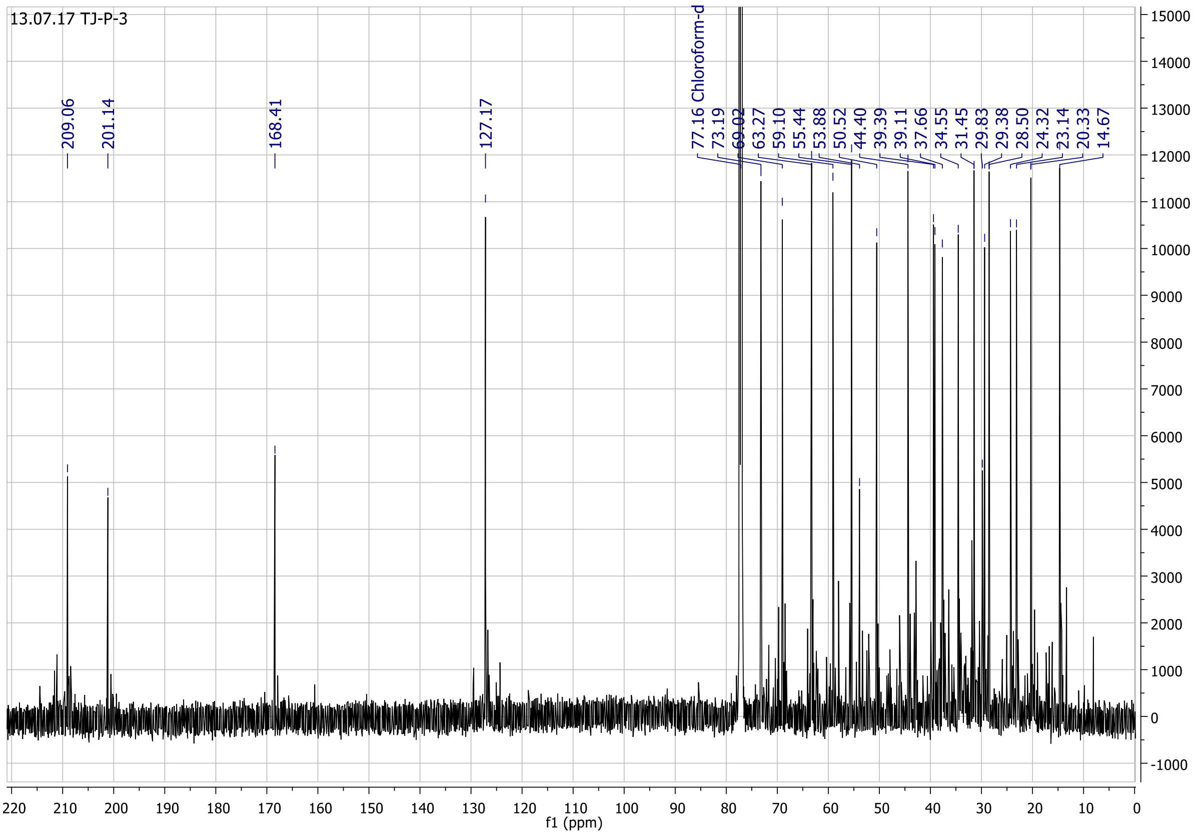

Figure.S2. ^{13}C NMR spectral of 6β,11α-dihydroxyprogesterone (**2**) ($CDCl_3$, 151 MHz)

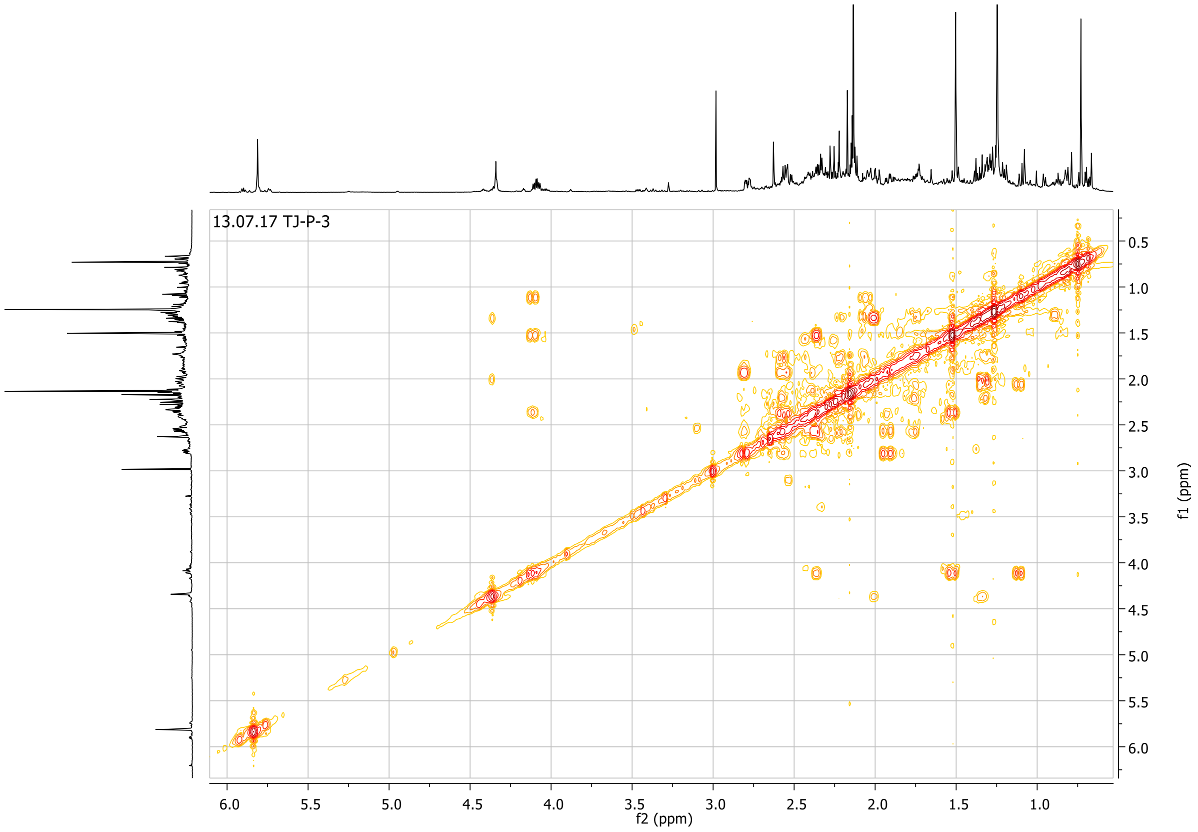

Figure.S3. COSY spectral of 6β,11α-dihydroxyprogesterone (**2**) ($CDCl_3$, 600 MHz)

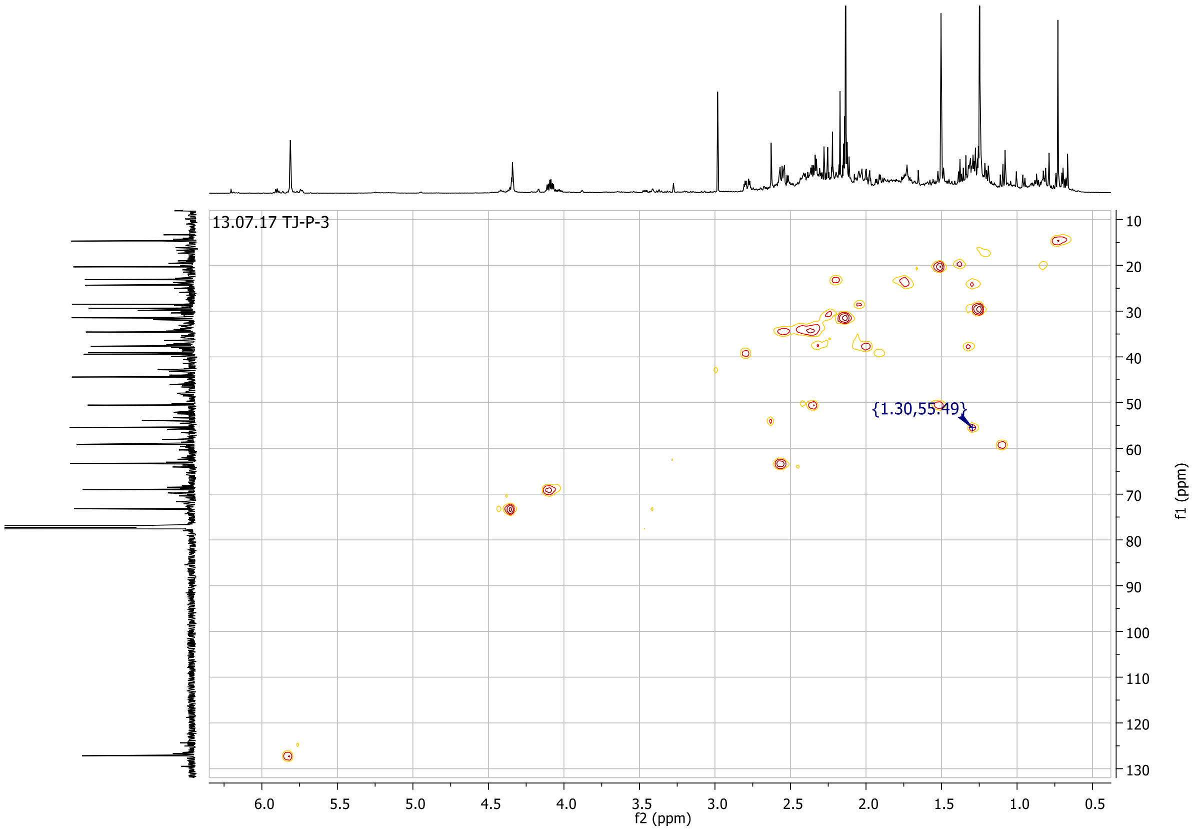

Figure.S4. HSQC spectral of 6β,11α-dihydroxyprogesterone (**2**) ($CDCl_3$, 151 MHz)

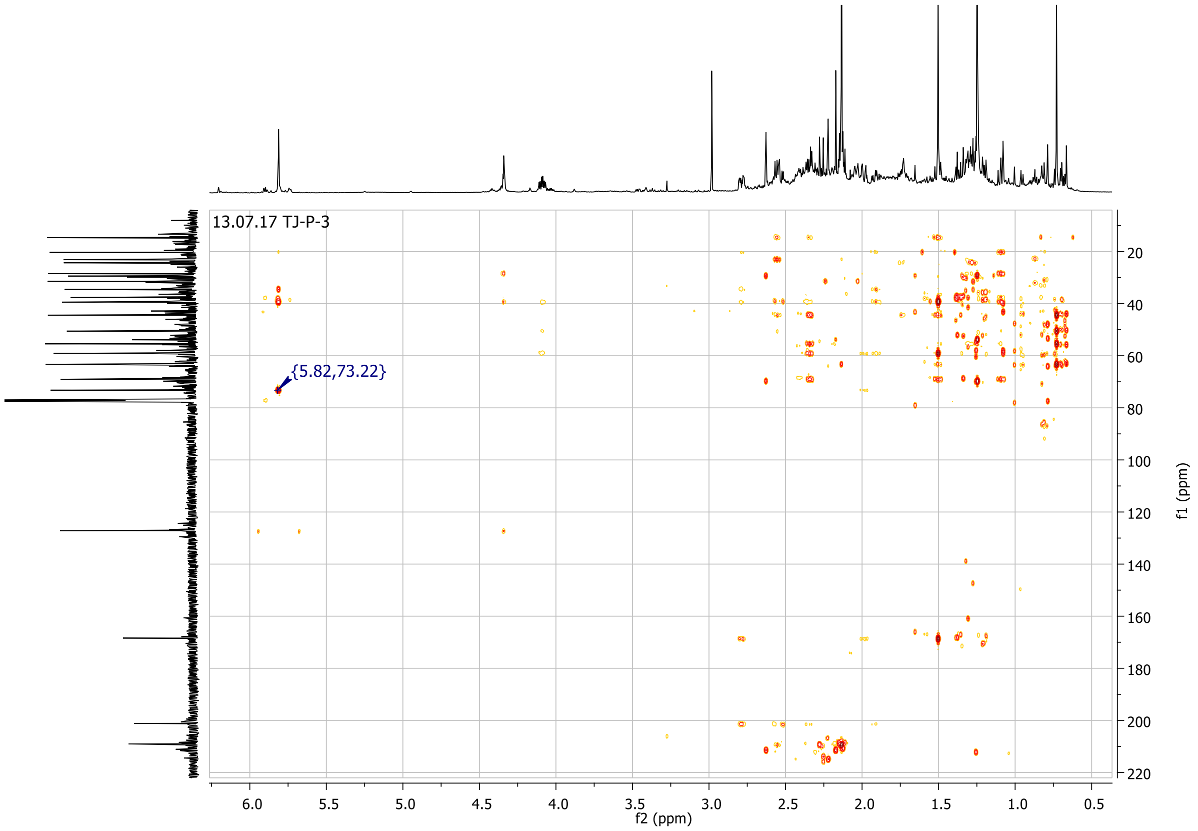

Figure.S5. HMBC spectral of 6β,11α-dihydroxyprogesterone (**2**) ($CDCl_3$, 151 MHz)

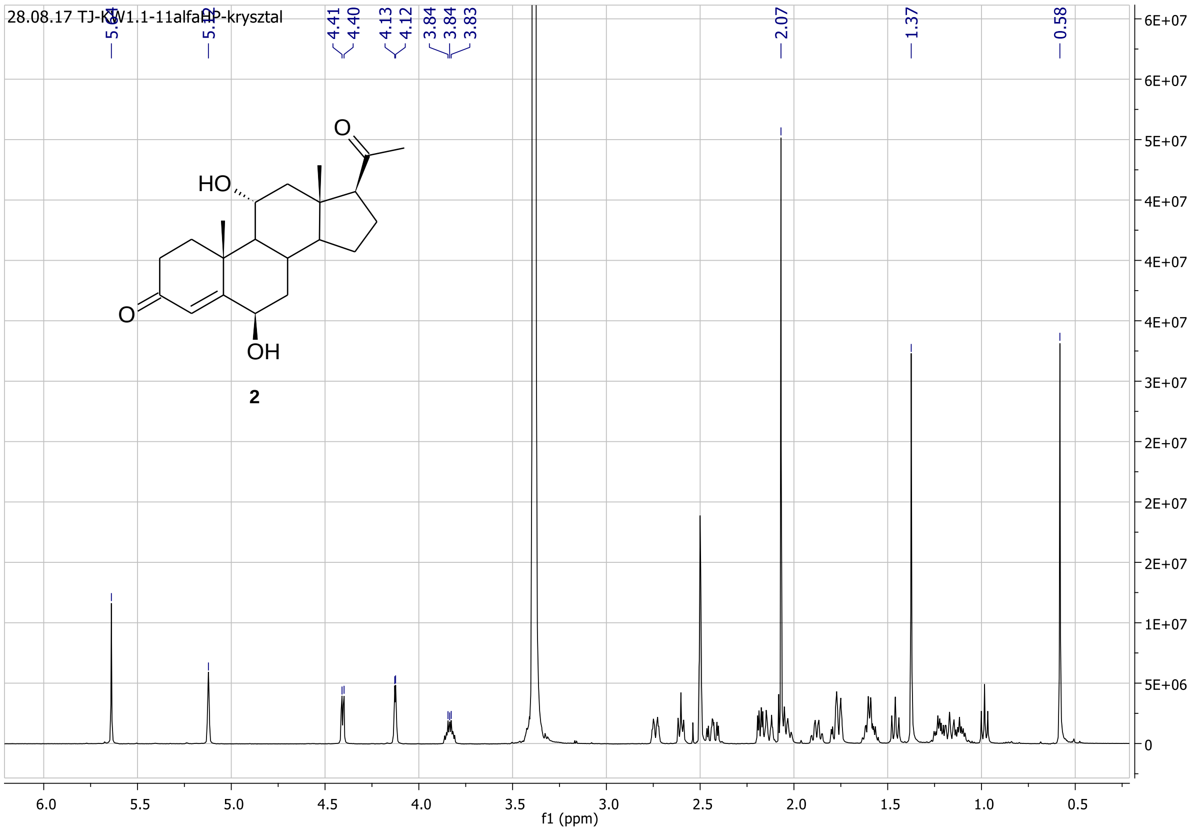

Figure.S6. ^{1}H NMR spectral of 6β,11α-dihydroxyprogesterone (**2**) (DMSO-d_6, 600 MHz)

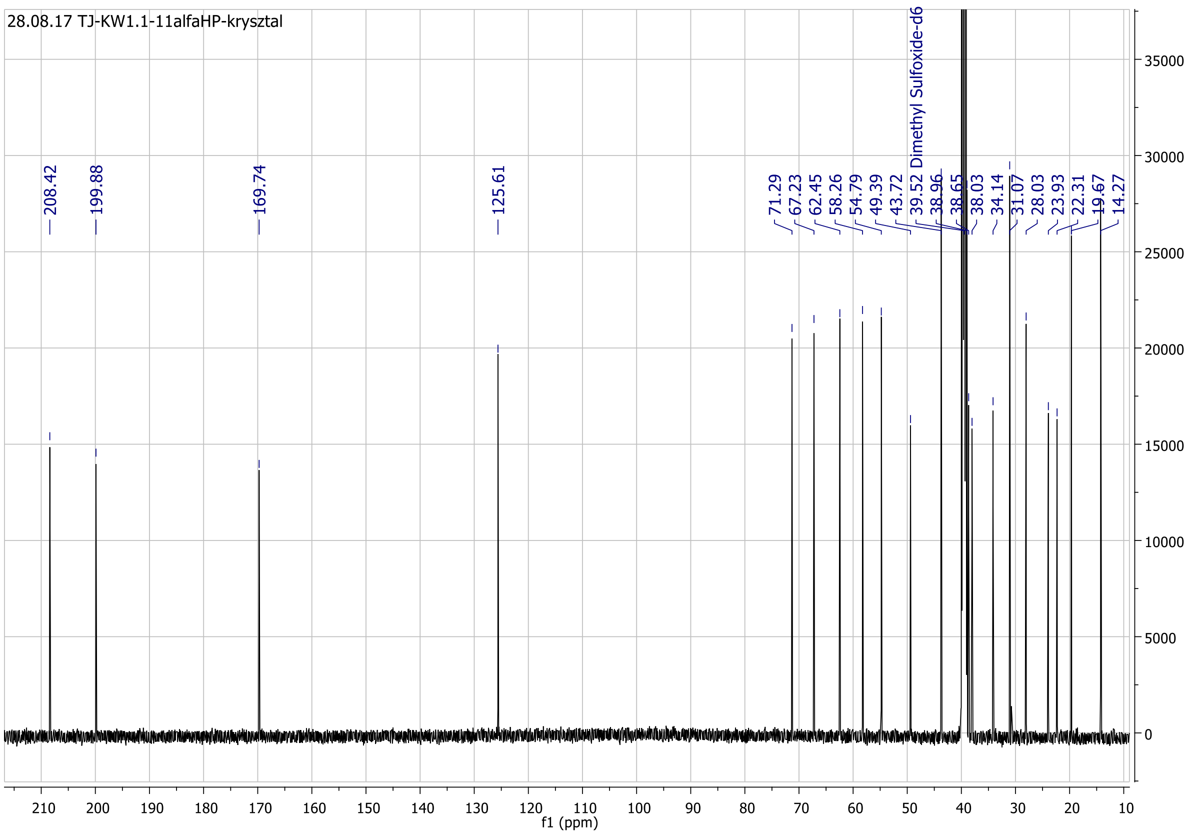

Figure.S7. ^{13}C NMR spectral of 6β,11α-dihydroxyprogesterone (**2**) (DMSO-d_6, 600 MHz)

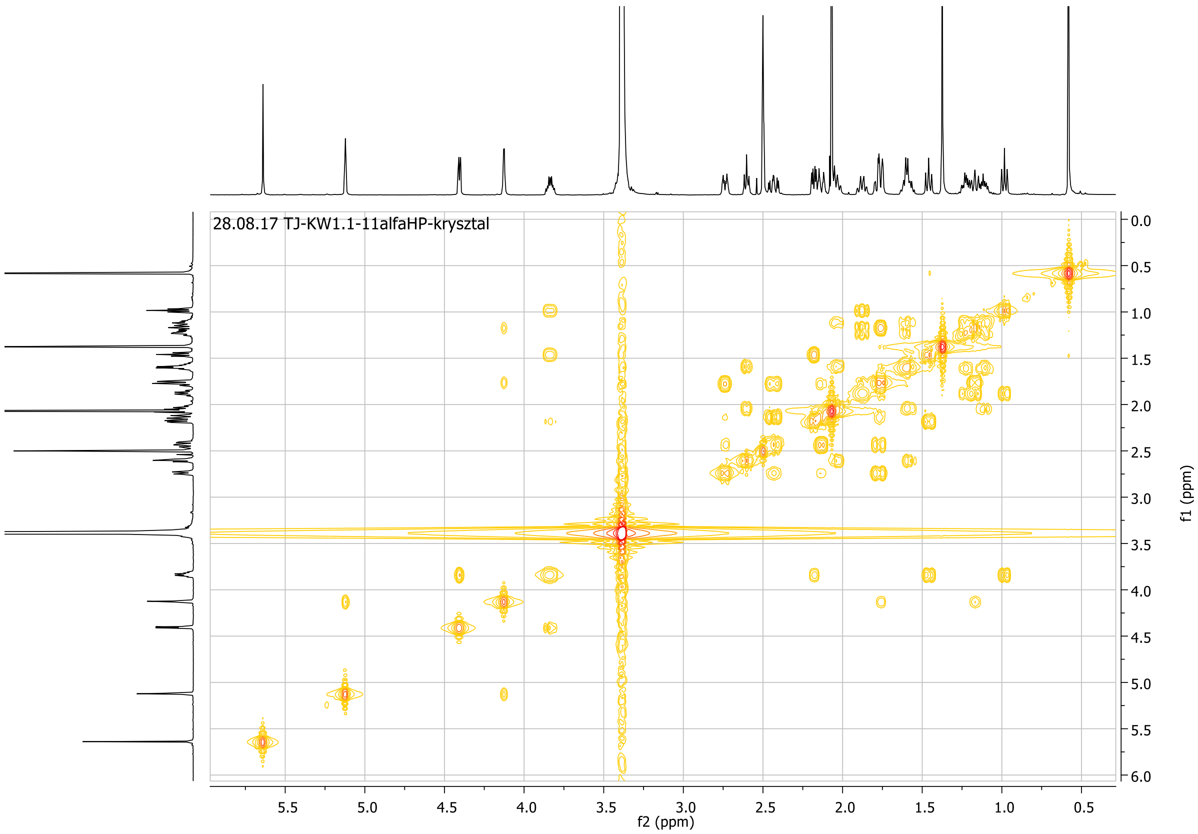

Figure.S8. COSY spectral of 6β,11α-dihydroxyprogesterone (**2**) (DMSO-d_6, 600 MHz)

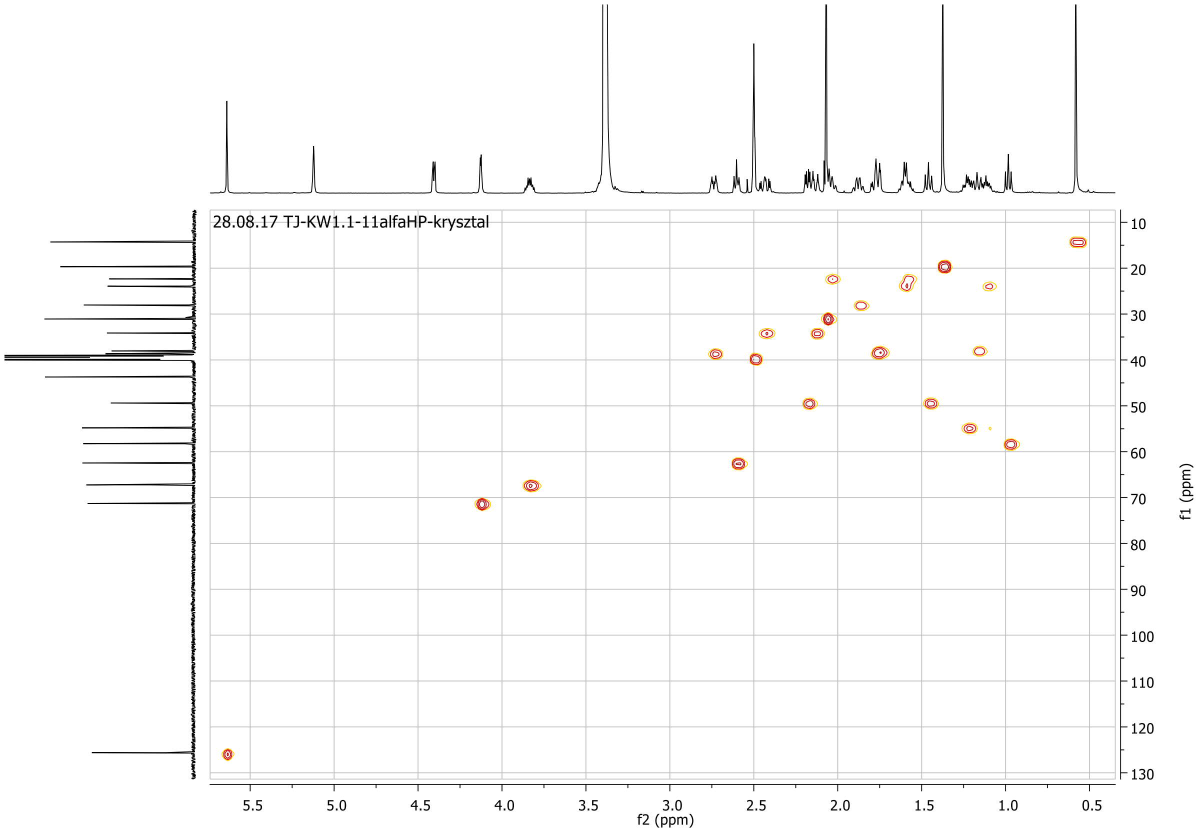

Figure.S9. HSQC spectral of 6β,11α-dihydroxyprogesterone (**2**) (DMSO-d_6, 600 MHz)

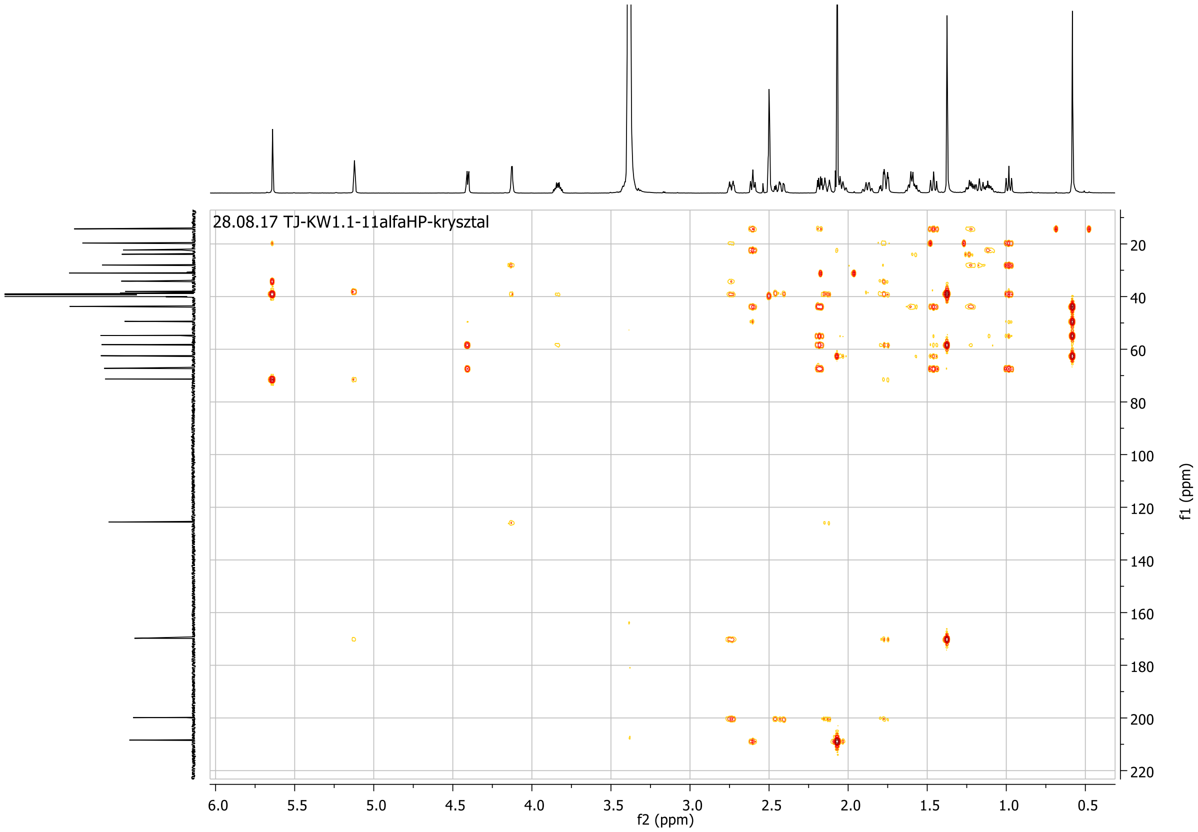

Figure.S10. HMBC spectral of 6β,11α-dihydroxyprogesterone (**2**) (DMSO-d_6, 600 MHz)

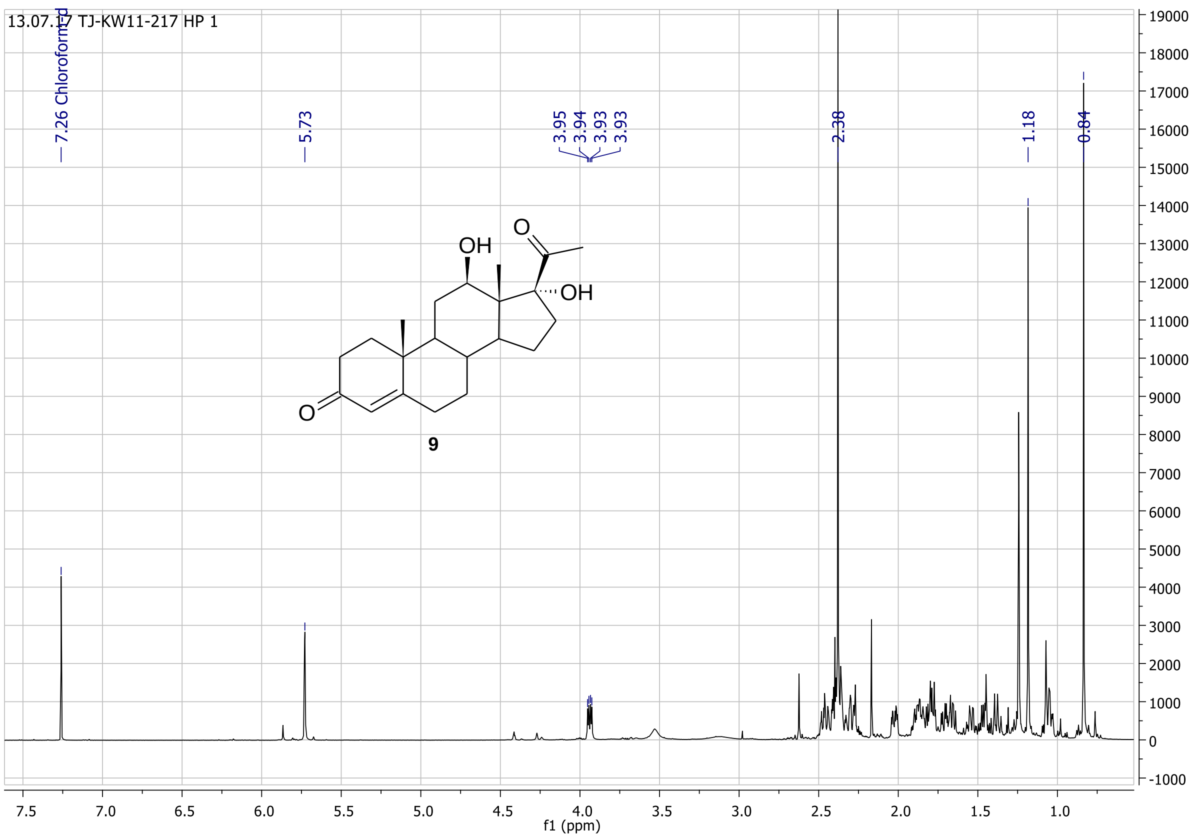

Figure.S11. ^{1}H NMR spectral of 12β,17α-dihydroxyprogesterone (**9**) ($CDCl_3$, 600 MHz)

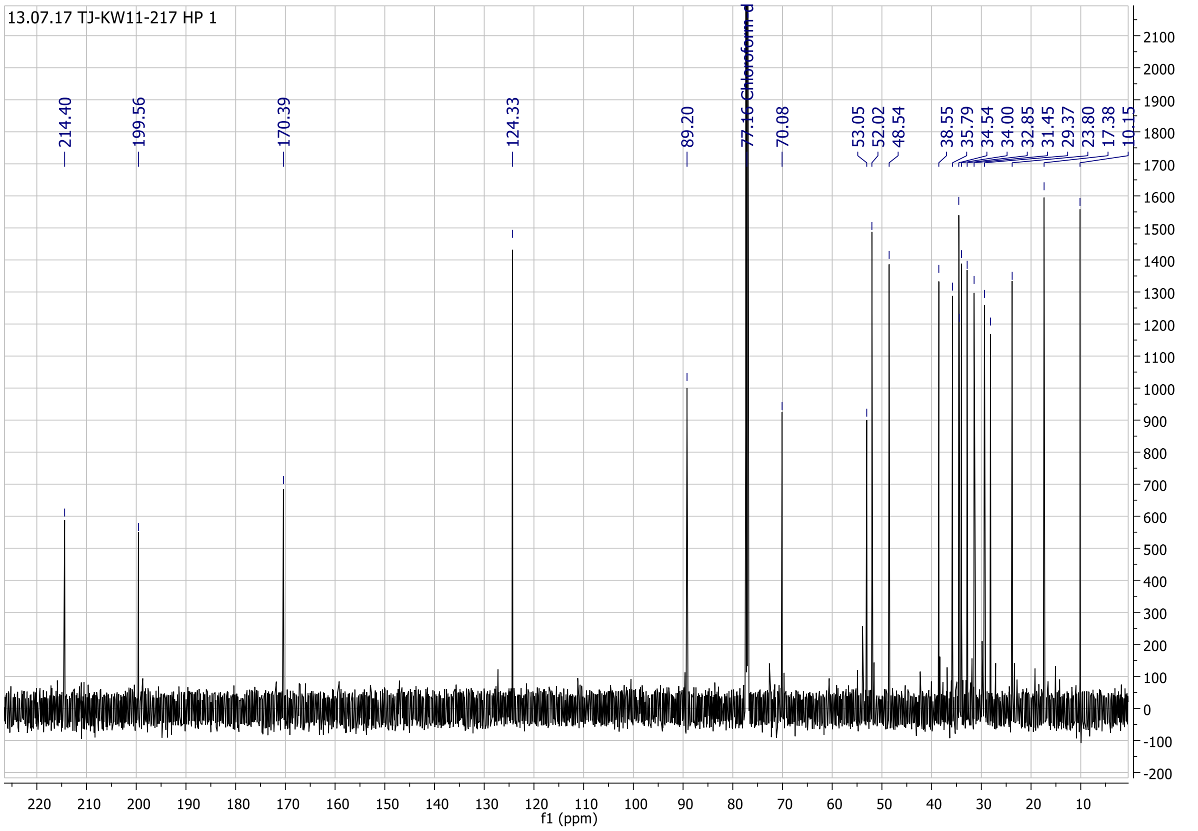

Figure.S12. ^{13}C NMR spectral of 12β,17α-dihydroxyprogesterone (**9**) ($CDCl_3$, 151 MHz)

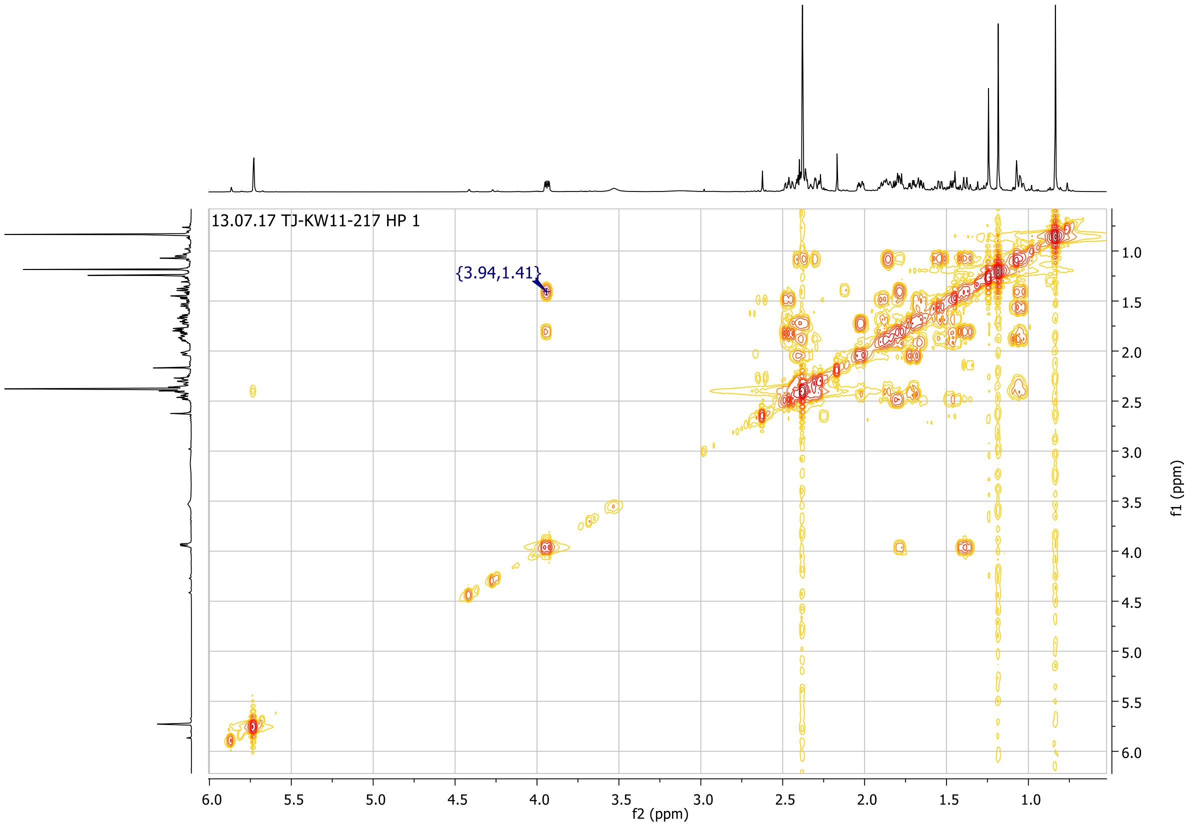

Figure.S13. COSY spectral of 12β,17α-dihydroxyprogesterone (**9**) ($CDCl_3$, 600 MHz)

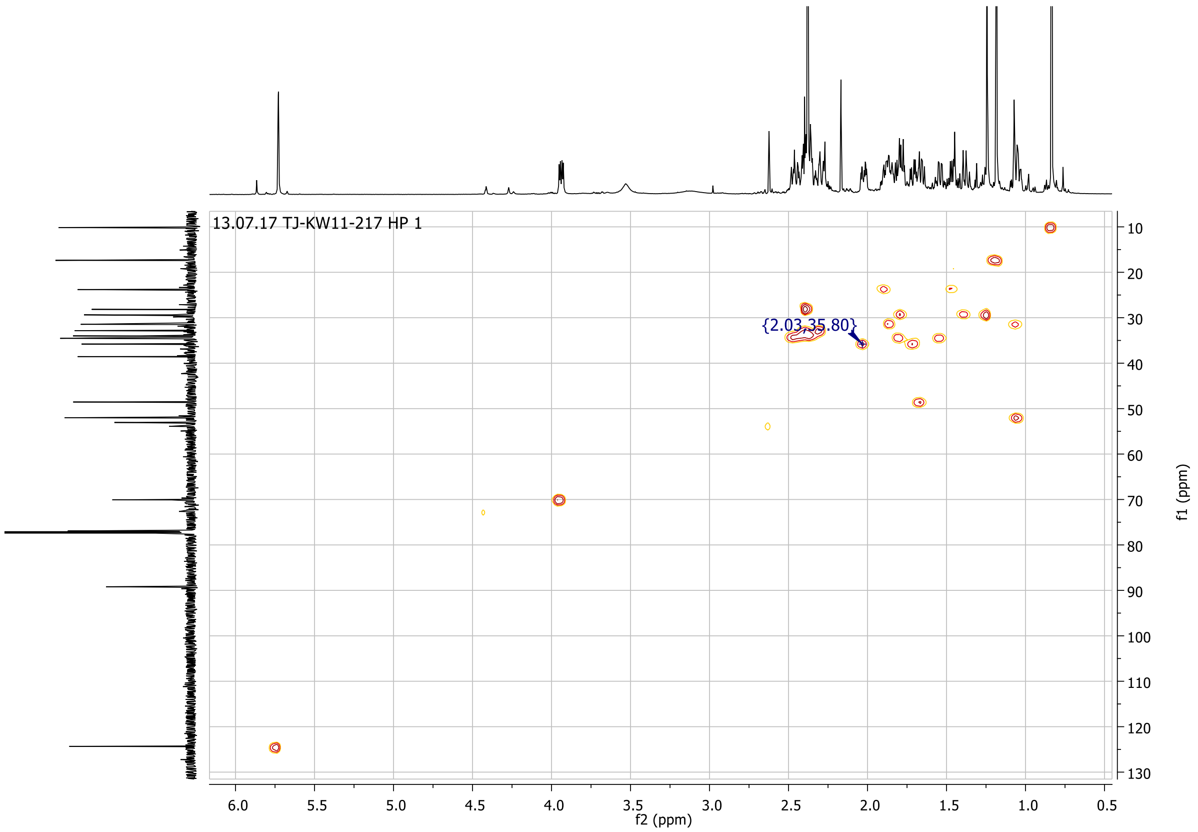

Figure.S14. HSQC spectral of 12β,17α-dihydroxyprogesterone (**9**) ($CDCl_3$, 151 MHz)

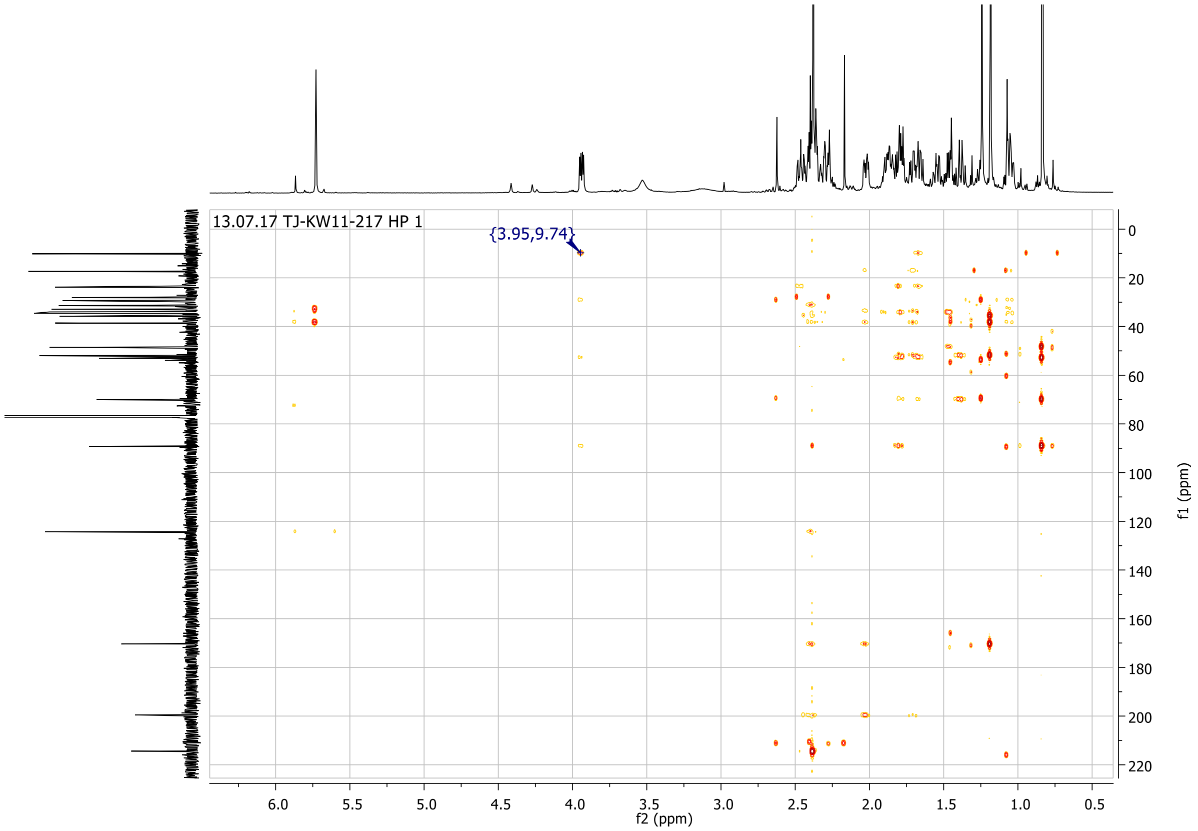

Figure.S15. HMBC spectral of 12β,17α-dihydroxyprogesterone (**9**) ($CDCl_3$, 151 MHz)

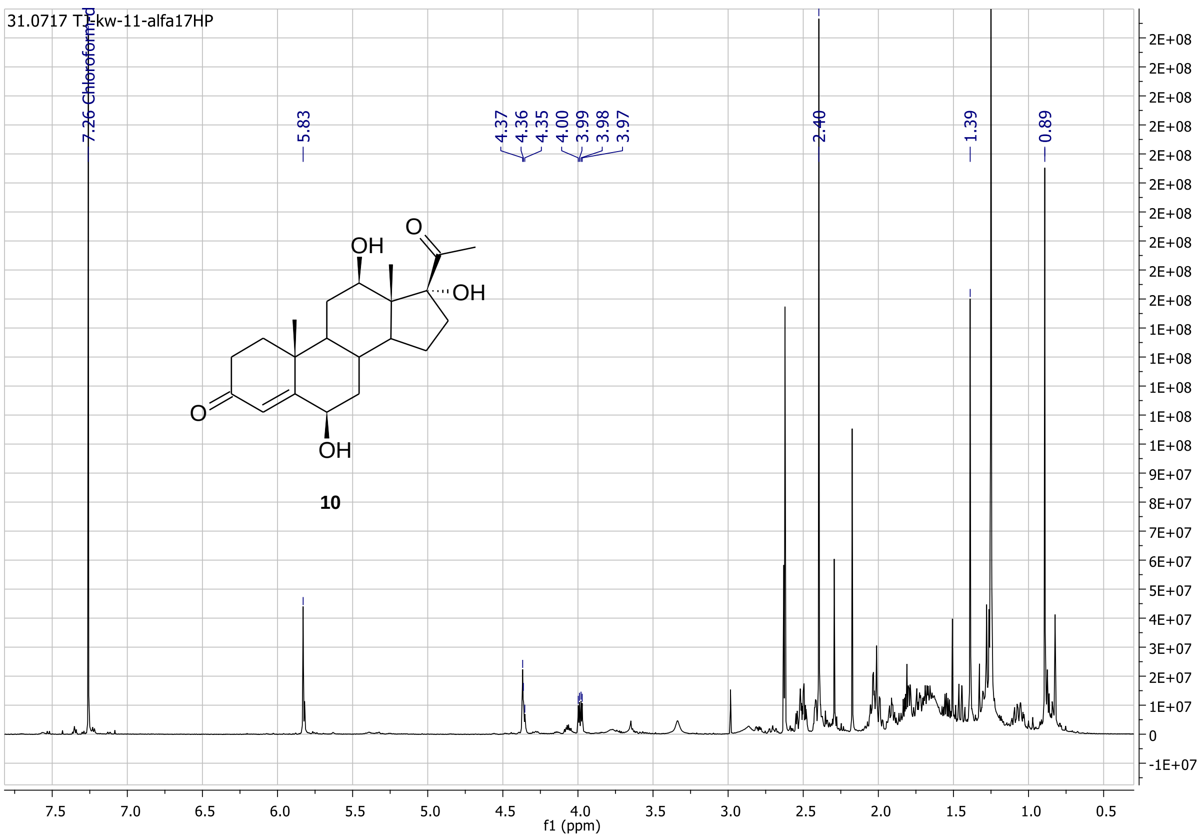

Figure.S16. ^{1}H NMR spectral of 6β,12β,17α-trihydroxyprogesterone (**10**) ($CDCl_3$, 600 MHz)

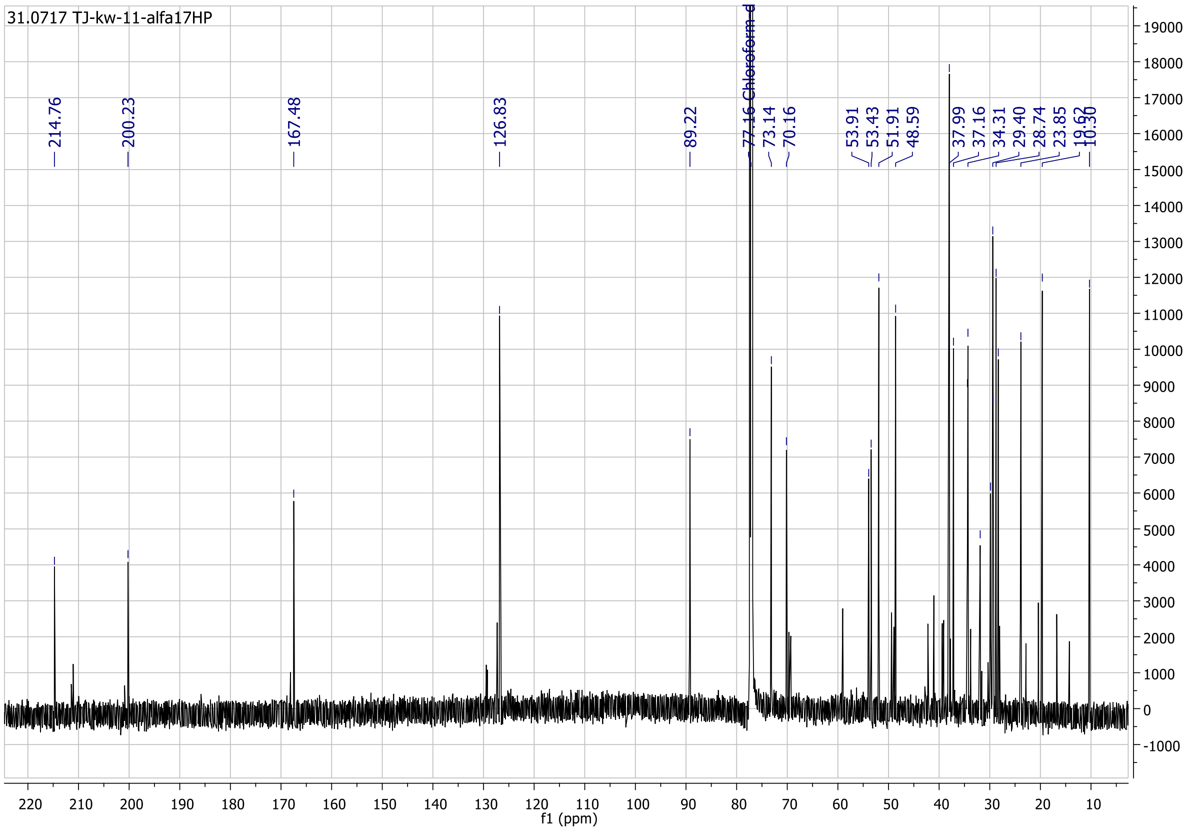

Figure.S17. ^{13}C NMR spectral of 6β,12β,17α-trihydroxyprogesterone (**10**) ($CDCl_3$, 151 MHz)

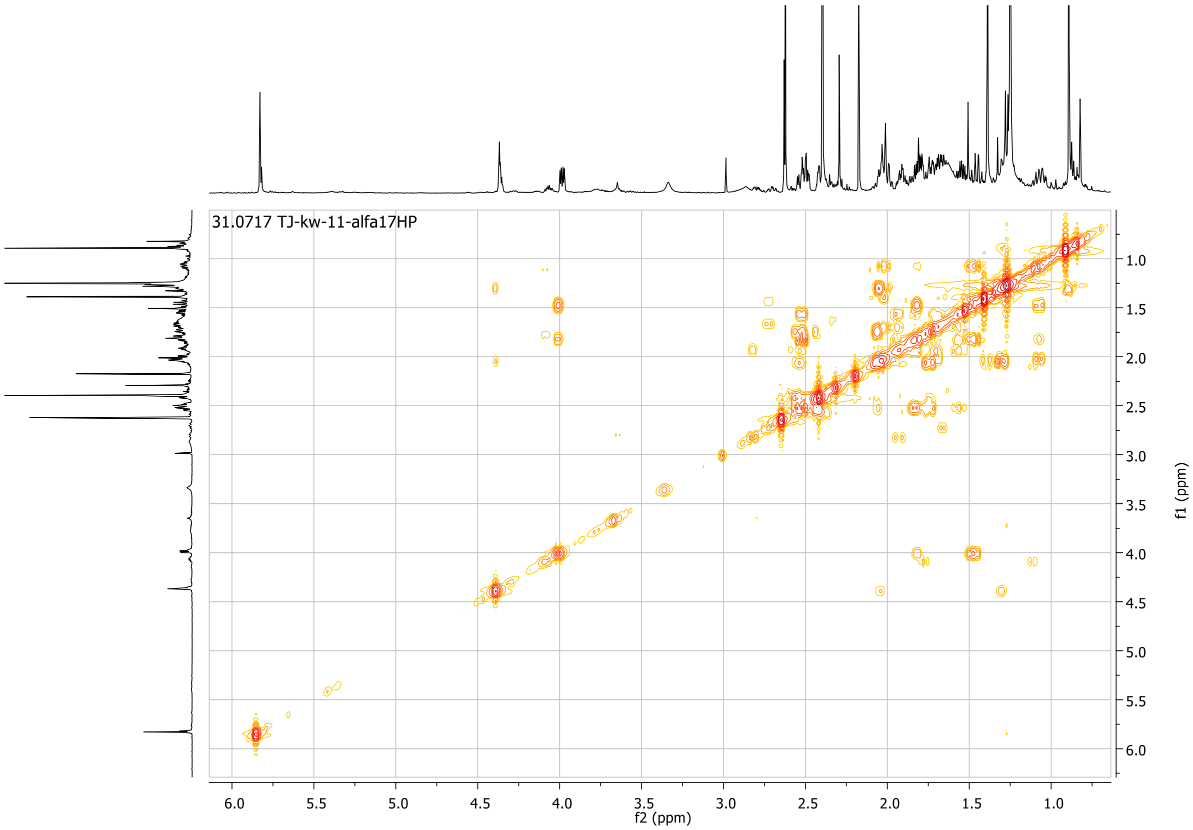

Figure.S18. COSY spectral of 6β,12β,17α-trihydroxyprogesterone (**10**) ($CDCl_3$, 600 MHz)

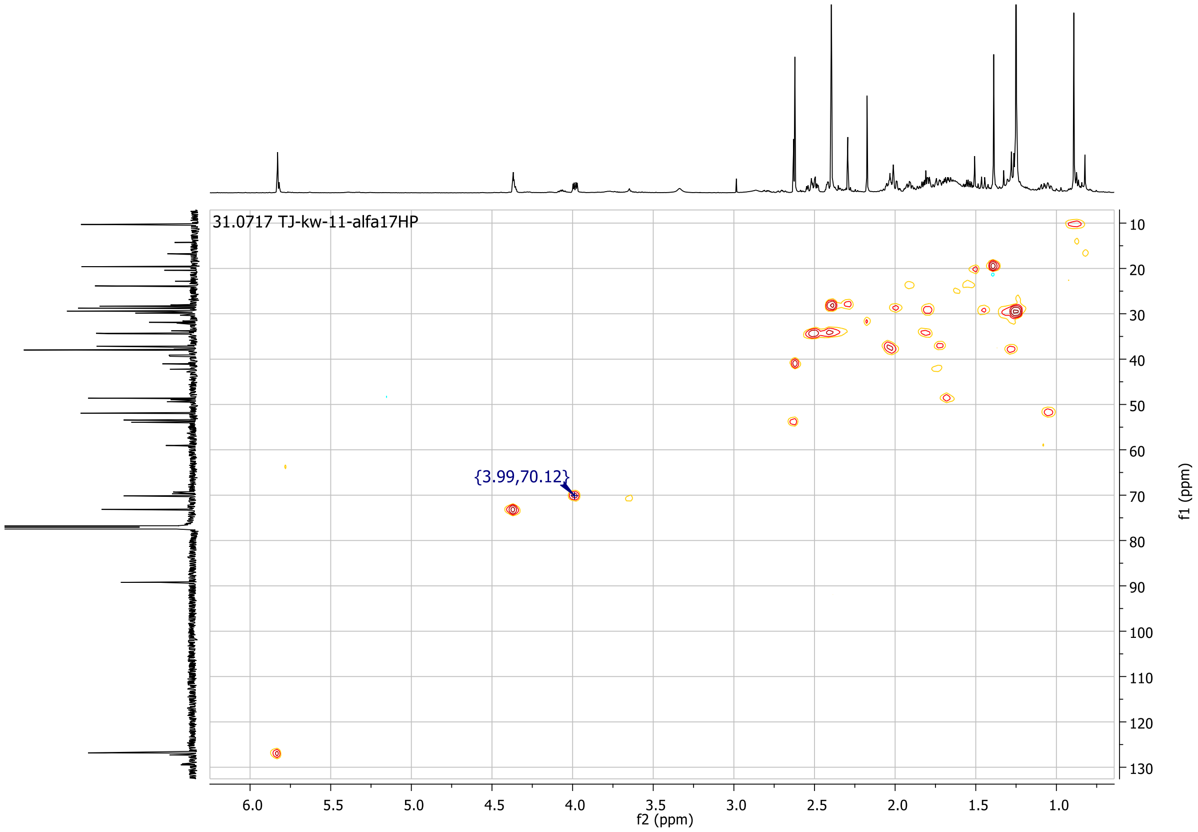

Figure.S19. HSQC spectral of 6β,12β,17α-trihydroxyprogesterone (**10**) ($CDCl_3$, 151 MHz)

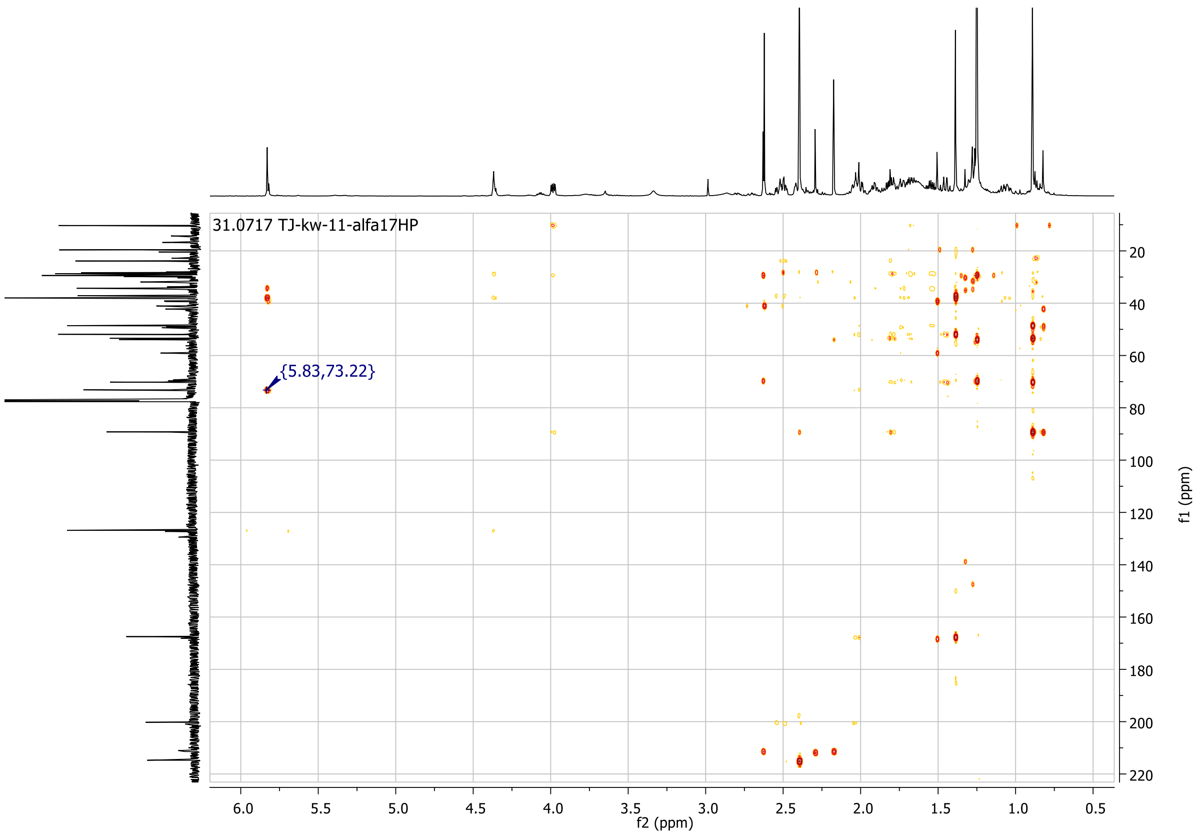

Figure.S20. HMBC spectral of 6β,12β,17α-trihydroxyprogesterone (**10**) ($CDCl_3$, 151 MHz)

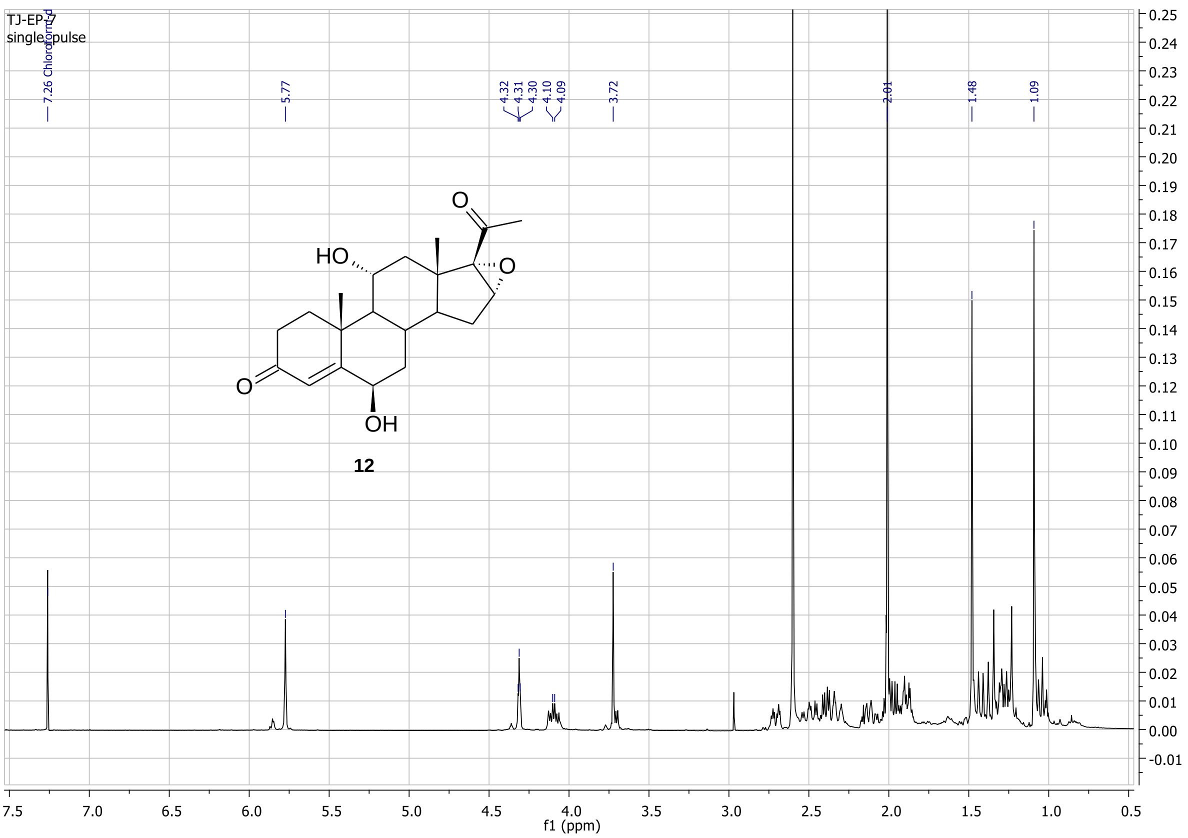

Figure.S21.^{1}H NMR spectral of 6β,11α-dihydroxy-16α,17α-epoxyprogesterone (**12**) ($CDCl_3$, 600 MHz)

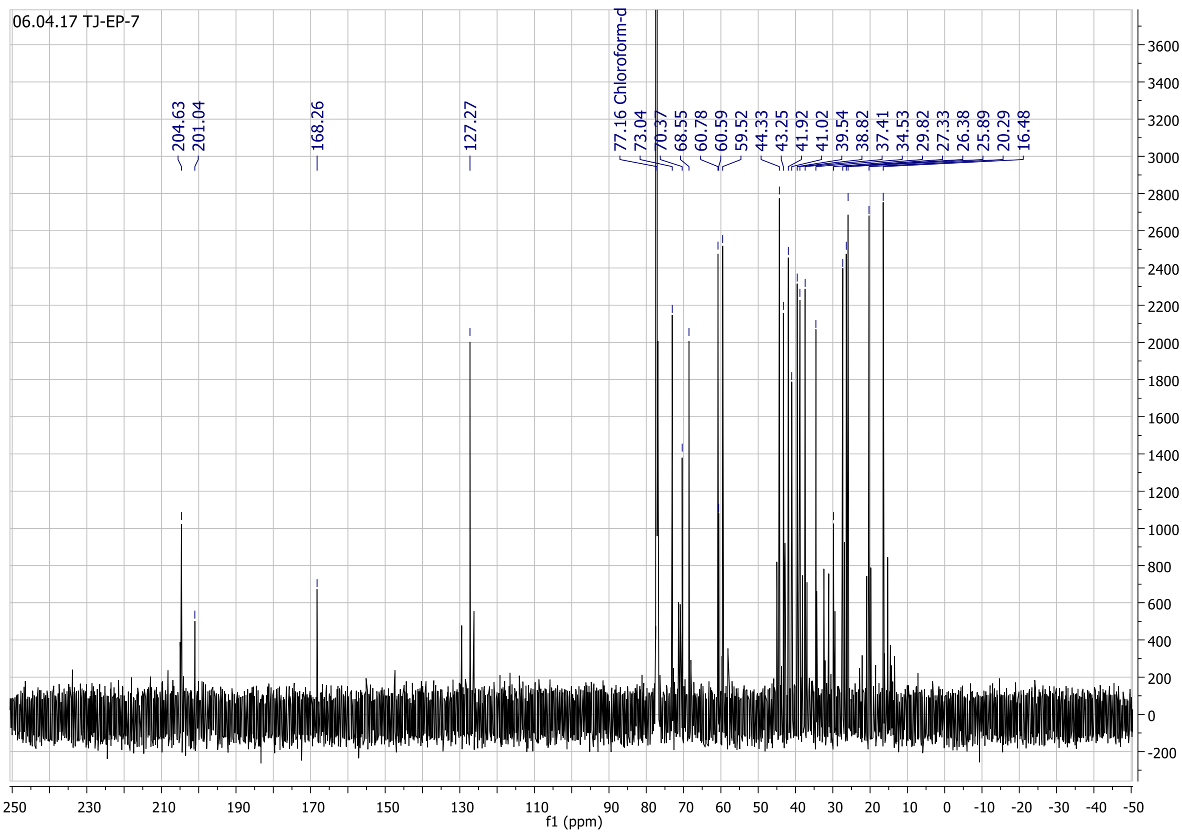

Figure.S22. ^{13}C NMR spectral of 6β,11α-dihydroxy-16α,17α-epoxyprogesterone (**12**) ($CDCl_3$, 151 MHz)

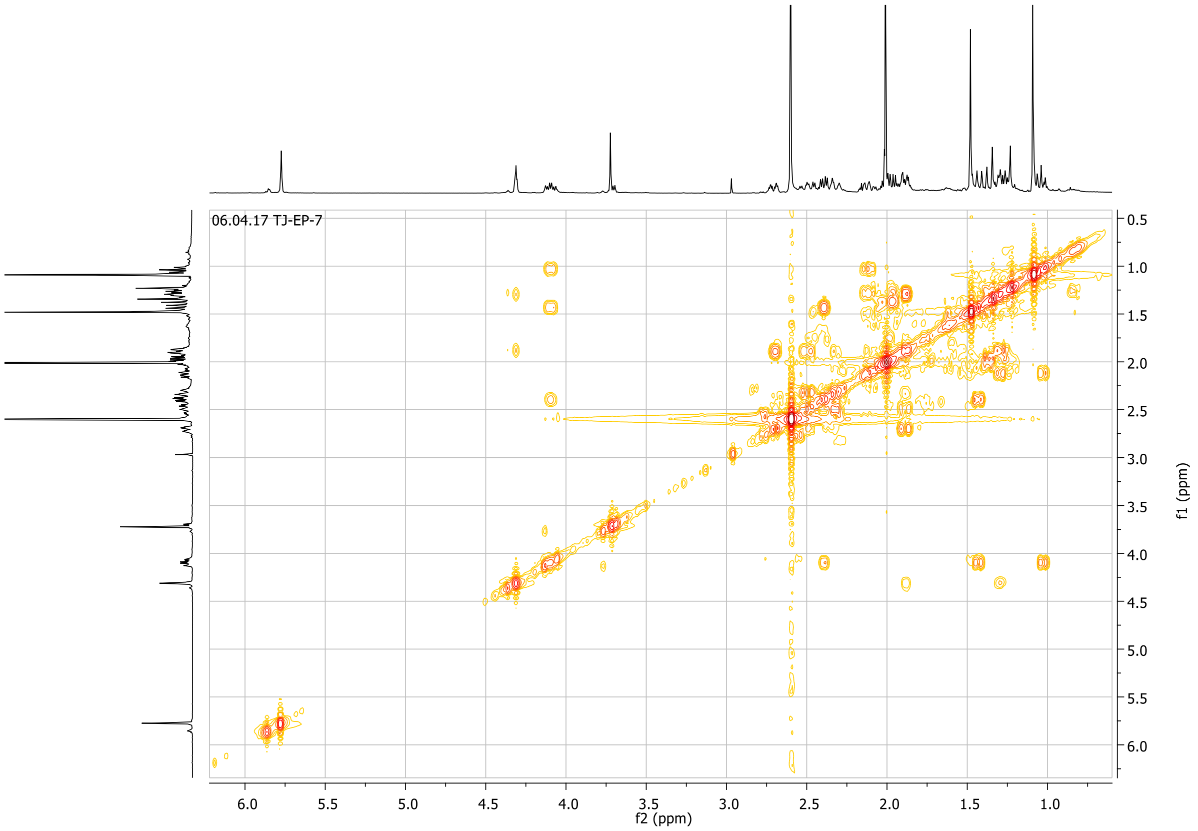

Figure.S23. COSY spectral of 6β,11α-dihydroxy-16α,17α-epoxyprogesterone (**12**) ($CDCl_3$, 600 MHz)

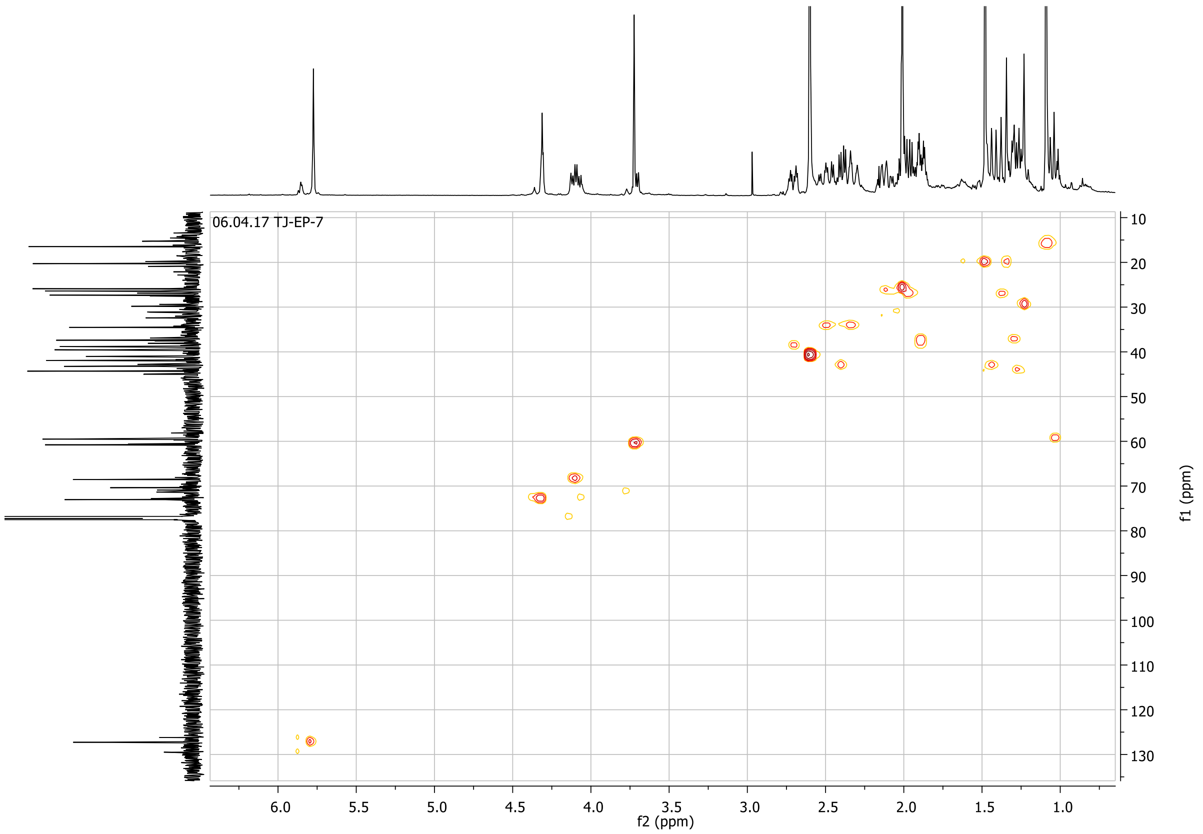

Figure.S24. HSQC spectral of 6β,11α-dihydroxy-16α,17α-epoxyprogesterone (**12**) ($CDCl_3$, 151 MHz)

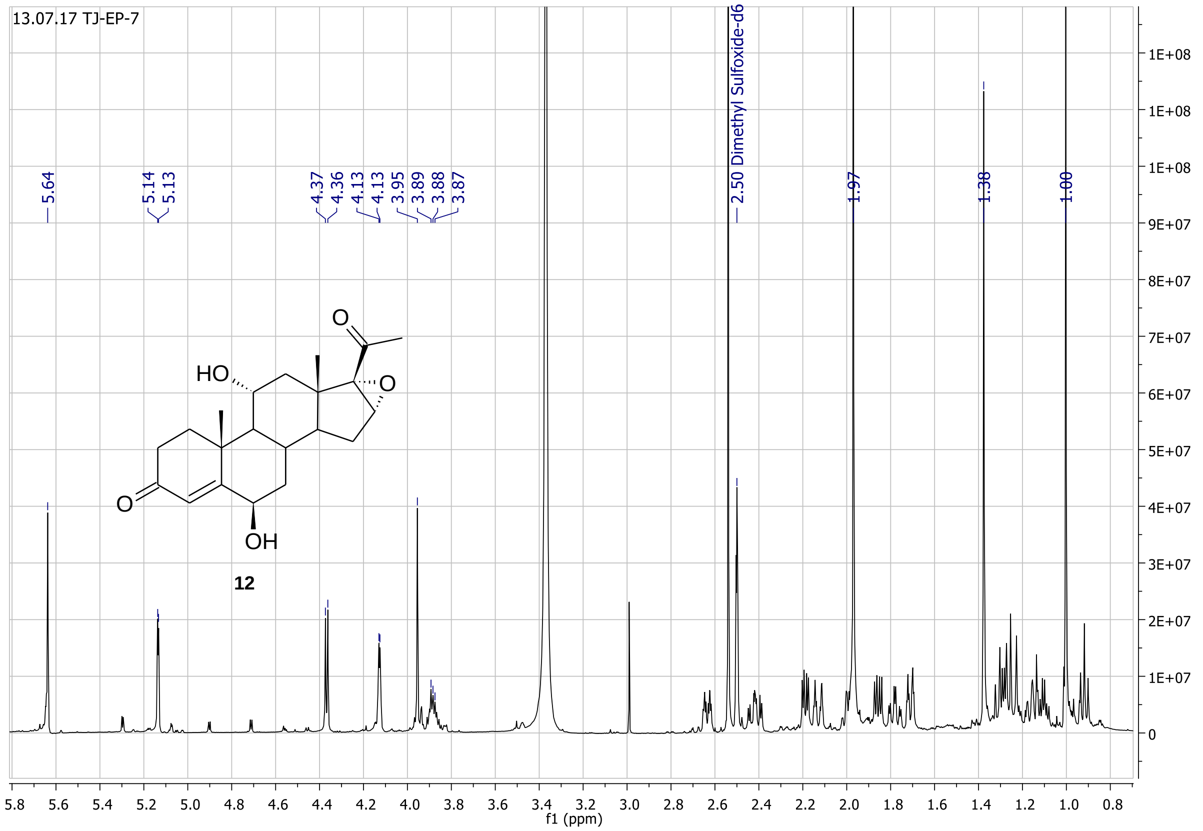

Figure.S25.^{1}H NMR spectral of 6β,11α-dihydroxy-16α,17α-epoxyprogesterone (**12**) (DMSO-d_6, 600 MHz)

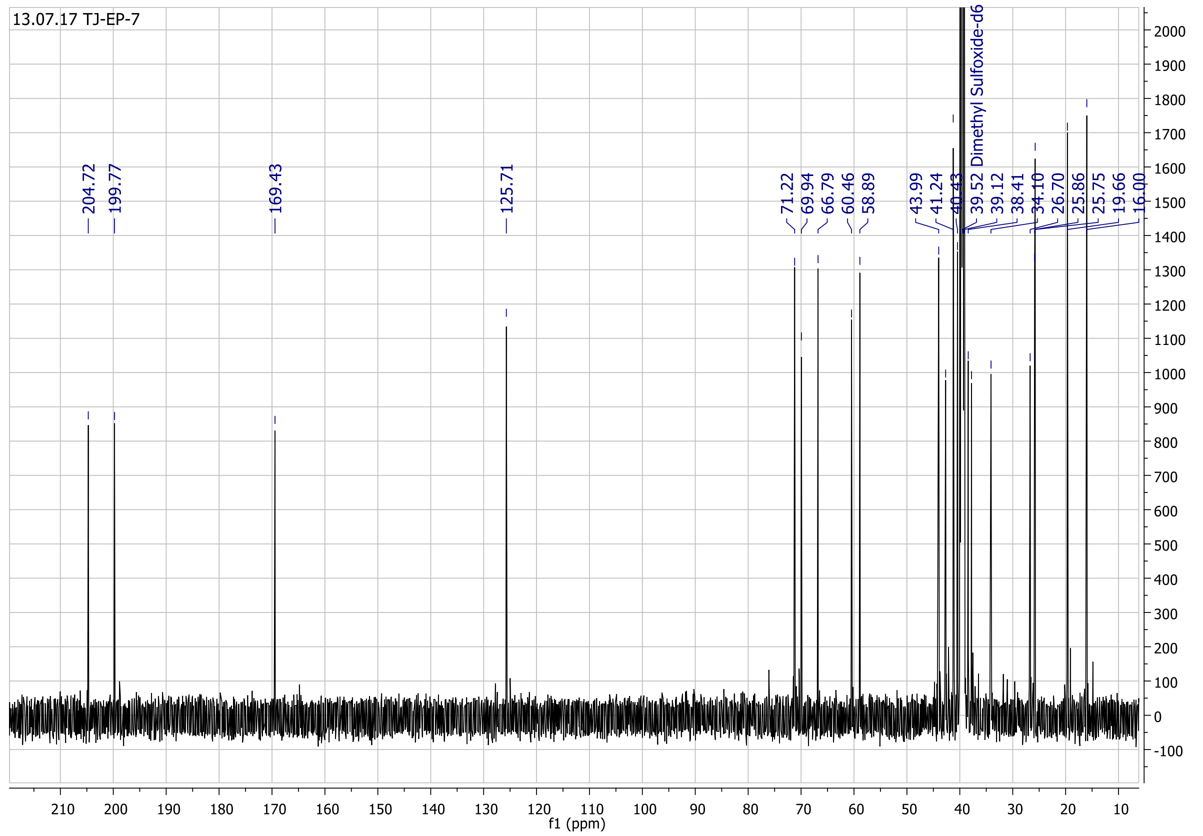

Figure.S26. ^{13}C NMR spectral of 6β,11α-dihydroxy-16α,17α-epoxyprogesterone (**12**) (DMSO-d_6, 600 MHz)

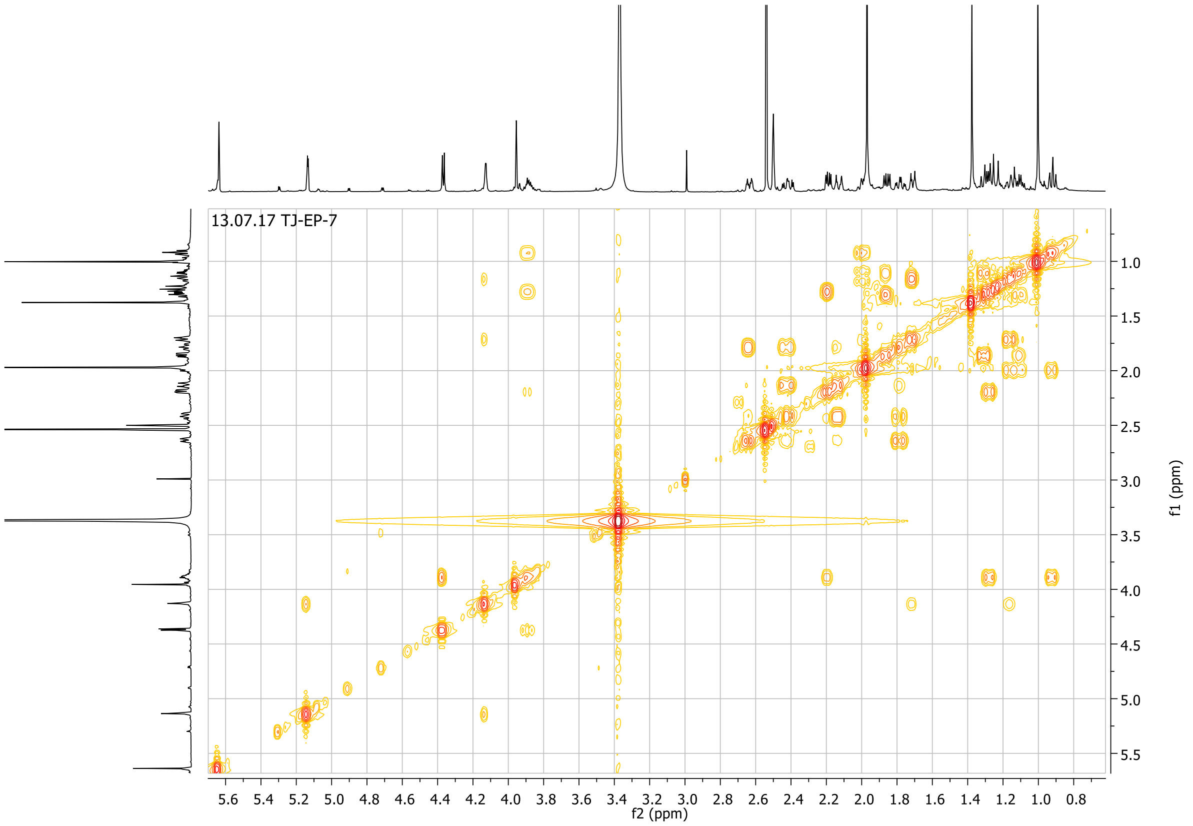

Figure.S27. COSY spectral of 6β,11α-dihydroxy-16α,17α-epoxyprogesterone (**12**) (DMSO-d_6, 600 MHz)

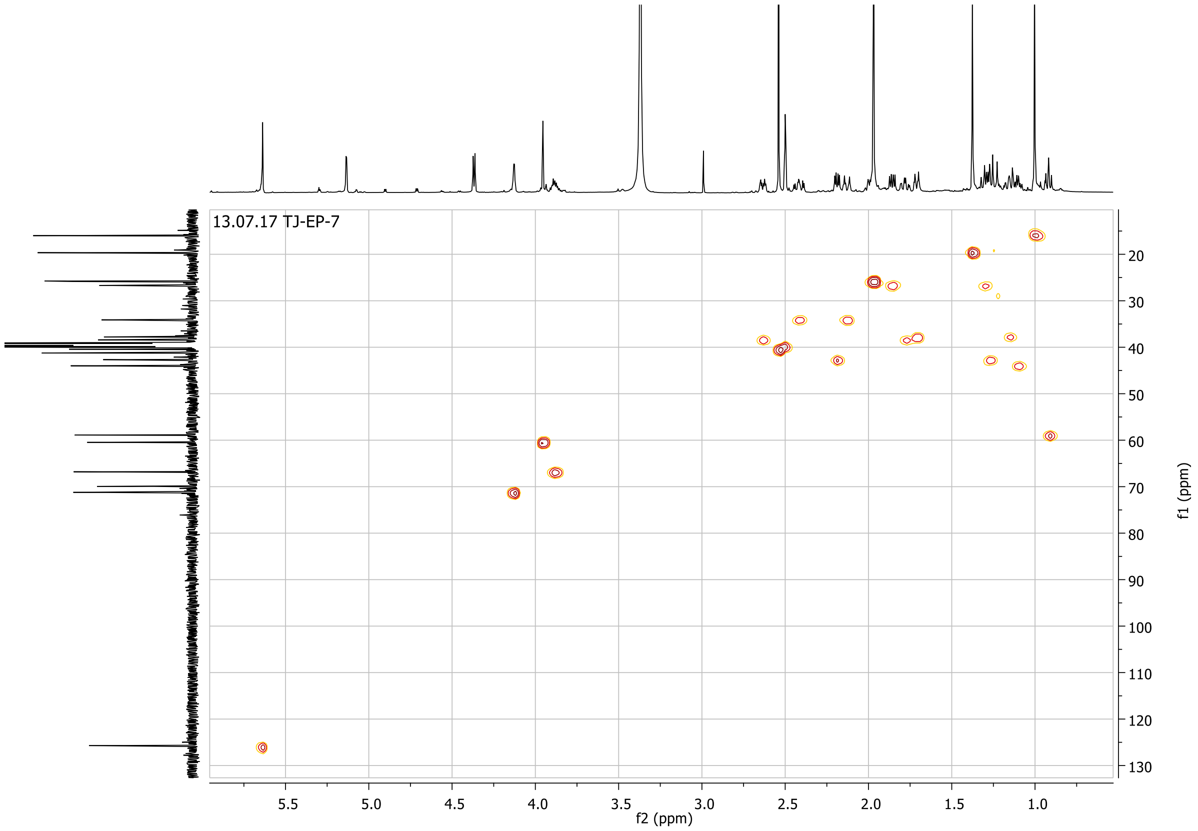

Figure.S28. HSQC spectral of 6β,11α-dihydroxy-16α,17α-epoxyprogesterone (**12**) (DMSO-d_6, 600 MHz)

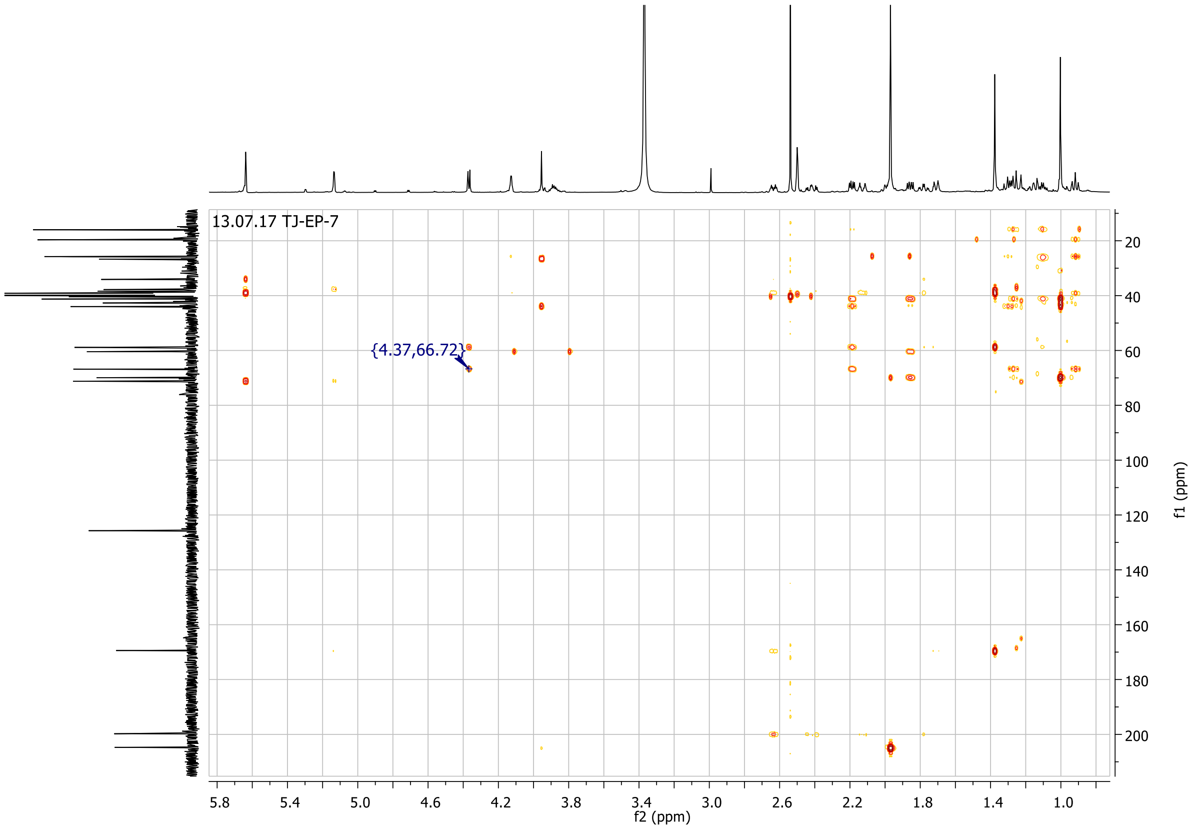

Figure.S29. HMBC spectral of 6β,11α-dihydroxy-16α,17α-epoxyprogesterone (**12**) (DMSO-d_6, 600 MHz)

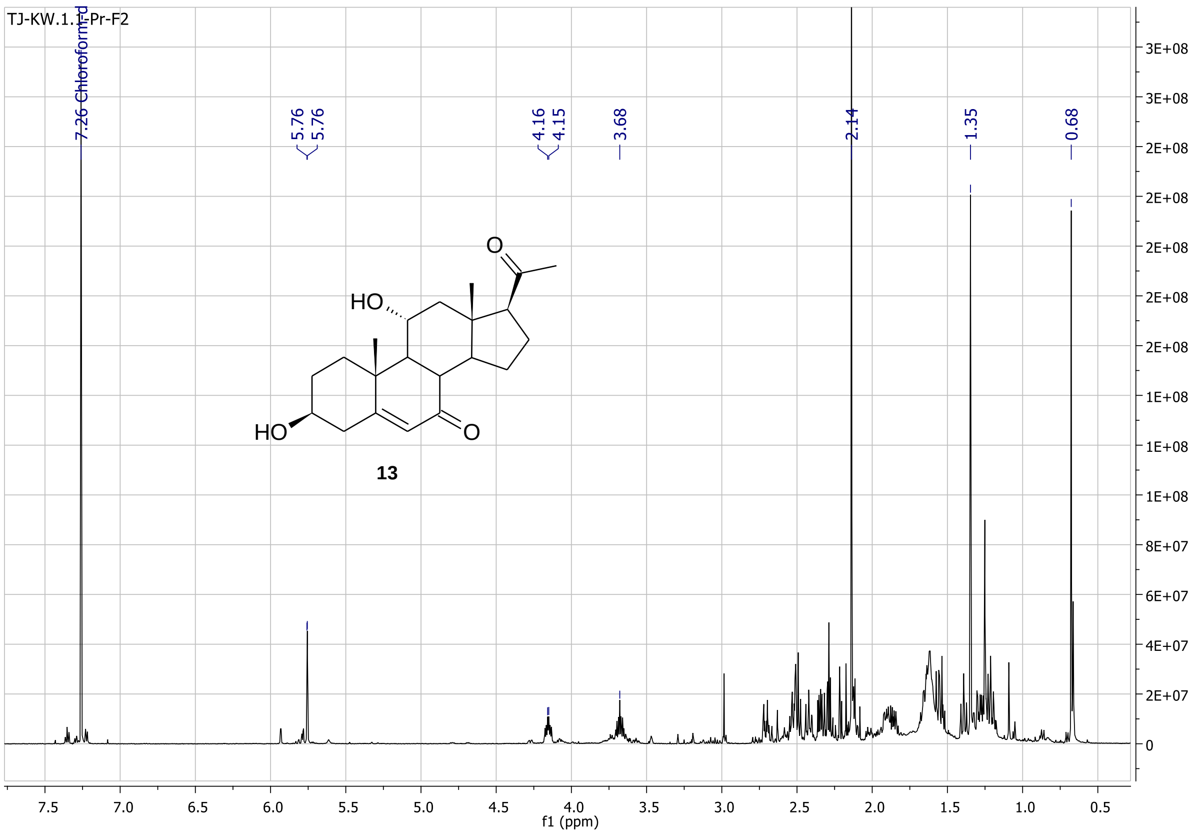

Figure.S30. ^{1}H NMR spectral of 11α-hydroxy-7-oxopregnenolone (**13**)($CDCl_3$, 600 MHz)

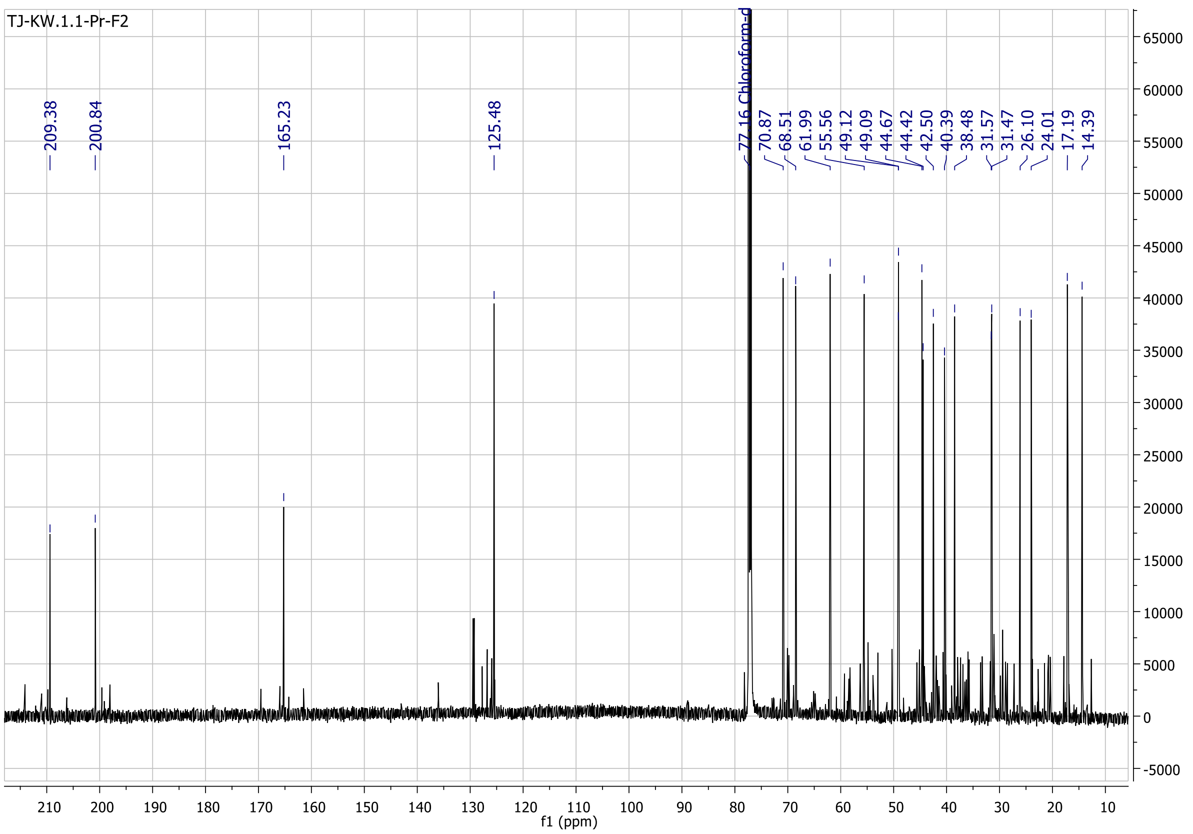

Figure.S31. ^{13}C NMR spectral of 11α-hydroxy-7-oxopregnenolone (**13**) ($CDCl_3$, 151 MHz)

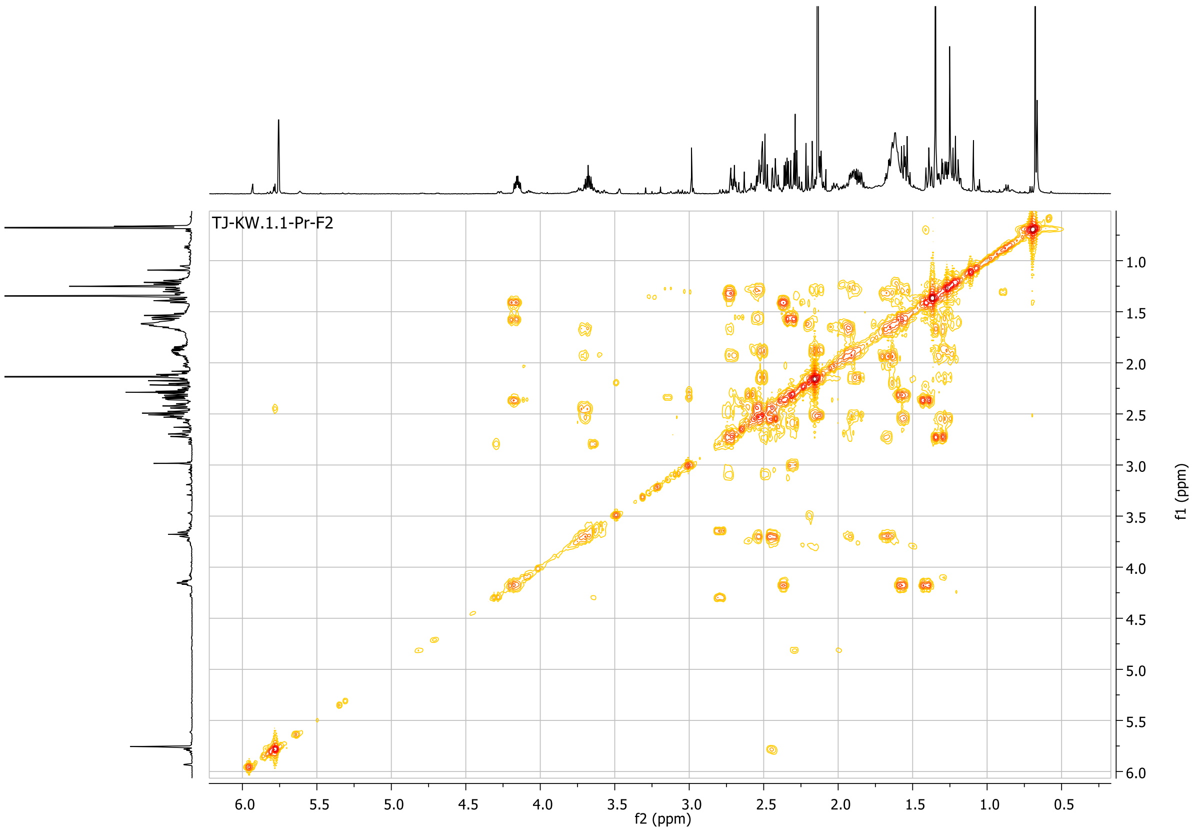

Figure.S32. COSY spectral of 11α-hydroxy-7-oxopregnenolone (**13**) ($CDCl_3$, 600 MHz)

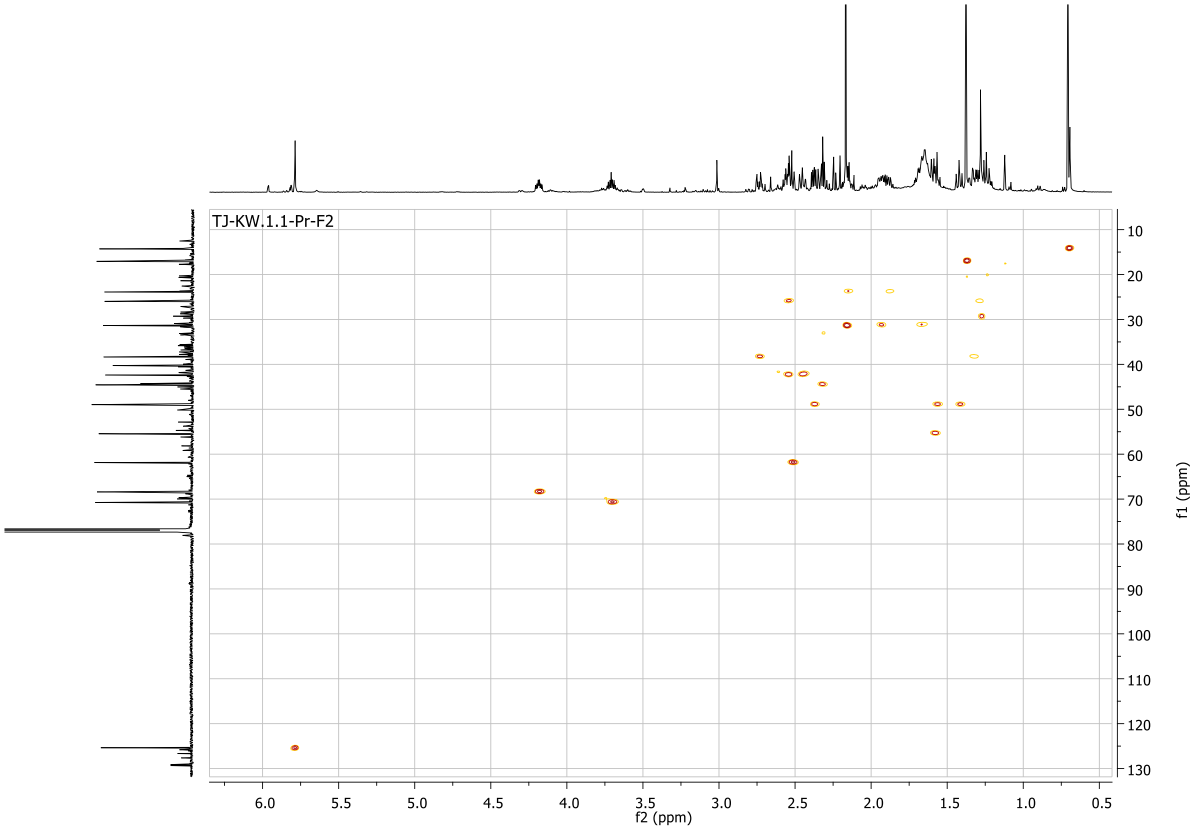

Figure.S33. HSQC spectral of 11α-hydroxy-7-oxopregnenolone (**13**) ($CDCl_3$, 151 MHz)

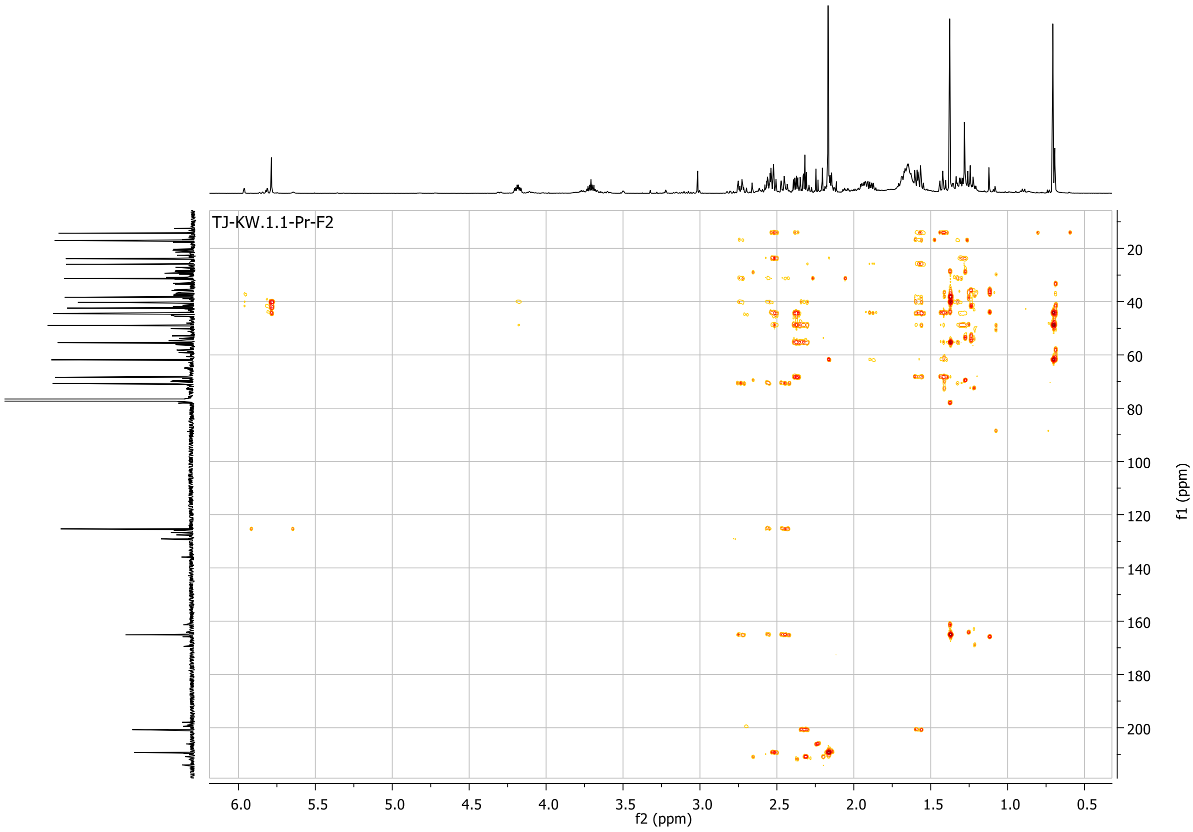

Figure.S34. HMBC spectral of 11α-hydroxy-7-oxopregnenolone (**13**) ($CDCl_3$, 151 MHz)

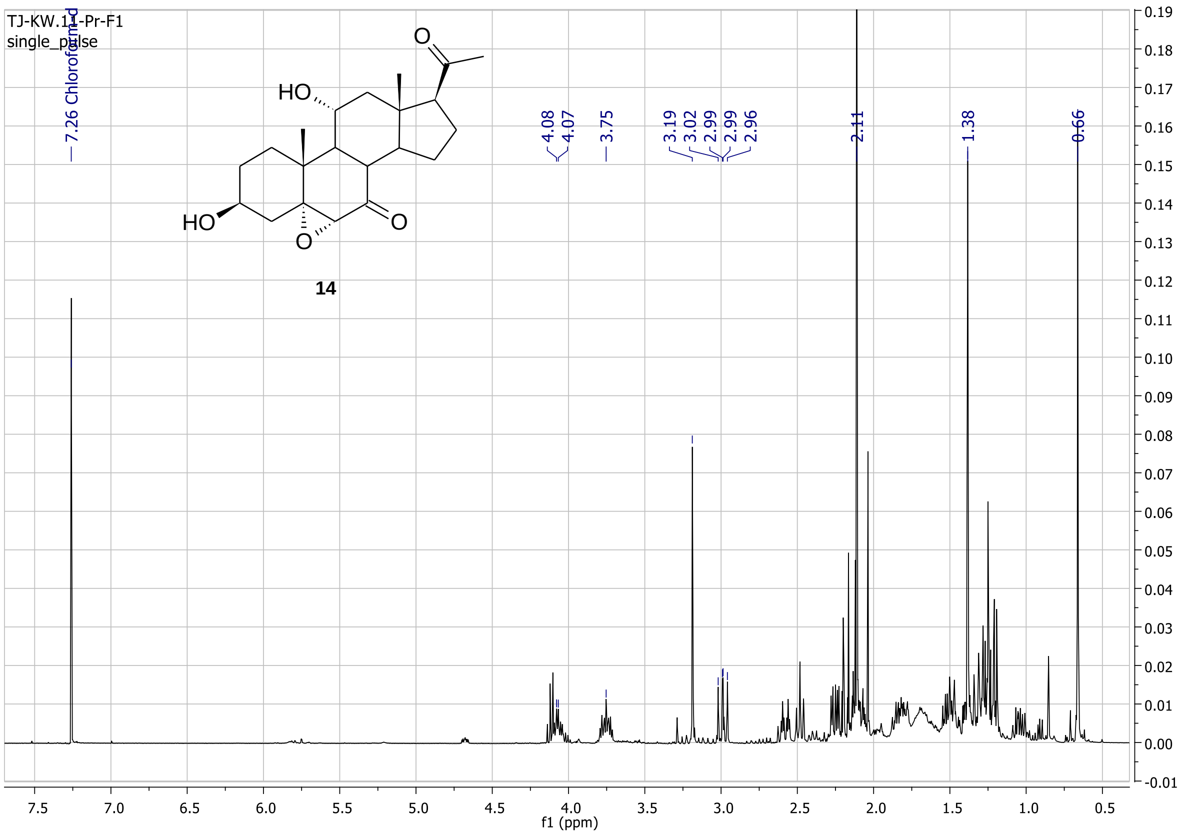

Figure.S35.^{1}H NMR spectral of 5α,6α-epoxy-3β,11α-dihydroxypregnan-7,20-dione (**14**)(CDCl₃, 600 MHz)

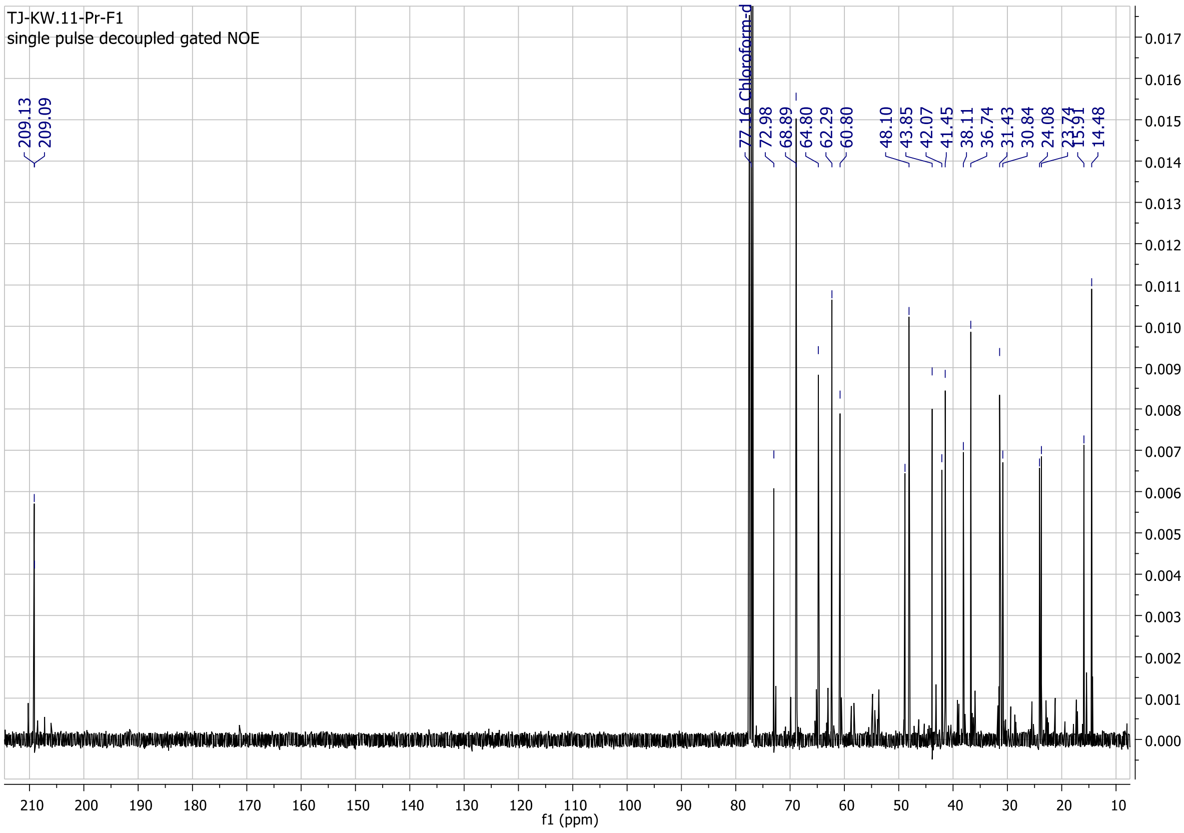

Figure.S36. ^{13}C NMR spectral of 5α,6α-epoxy-3β,11α-dihydroxypregnan-7,20-dione (**14**) (CDCl₃, 151 MHz)

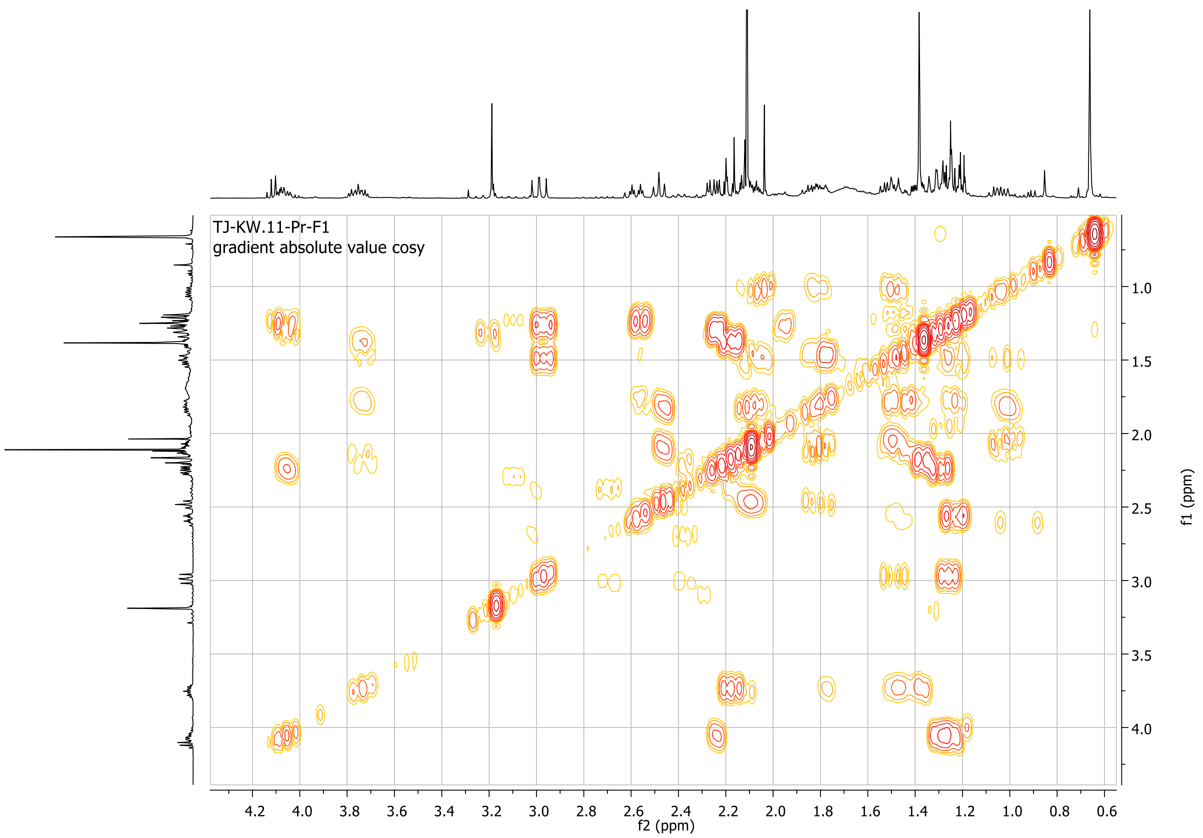

Figure.S37. COSY spectral of 5α,6α-epoxy-3β,11α-dihydroxypregnan-7,20-dione (**14**) ($CDCl_3$, 600 MHz)

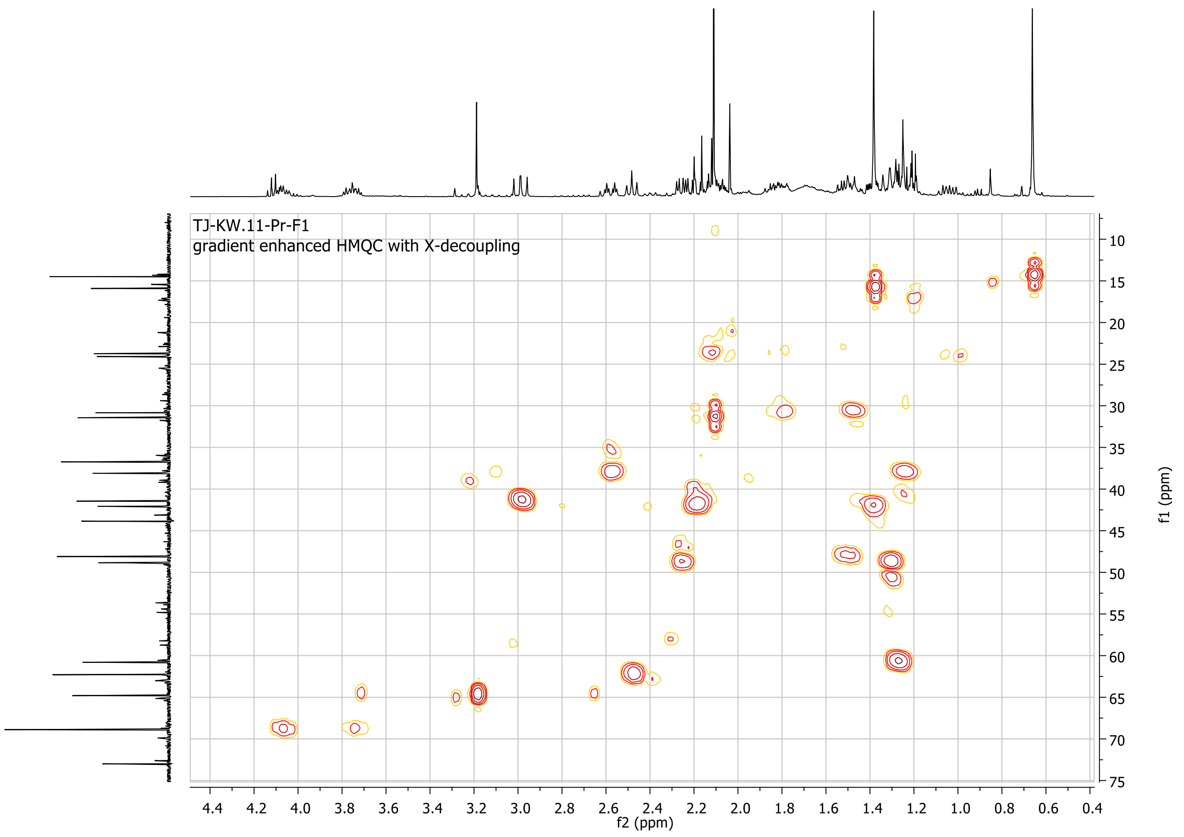

Figure.S38. HSQC spectral of 5α,6α-epoxy-3β,11α-dihydroxypregnan-7,20-dione (**14**) ($CDCl_3$, 151 MHz)

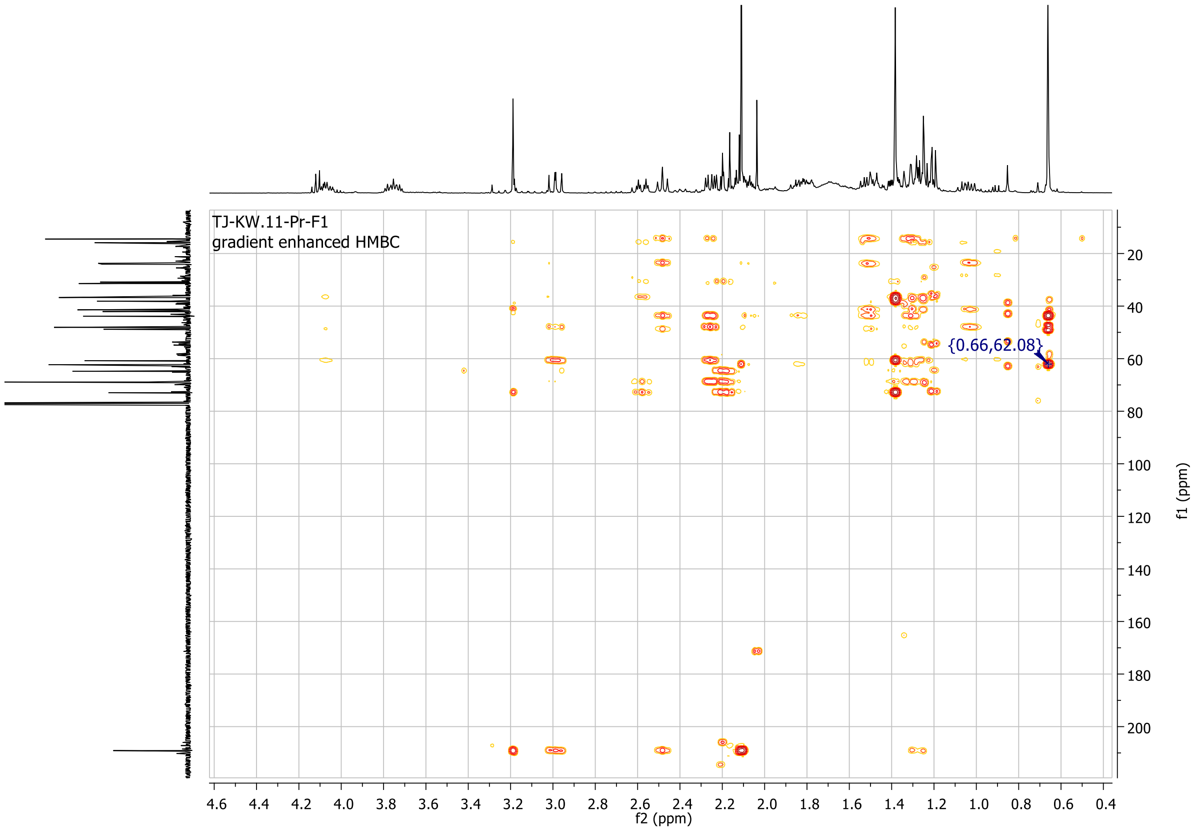

Figure.S39. HMBC spectral of 5α,6α-epoxy-3β,11α-dihydroxypregnan-7,20-dione (**14**) ($CDCl_3$, 151 MHz)

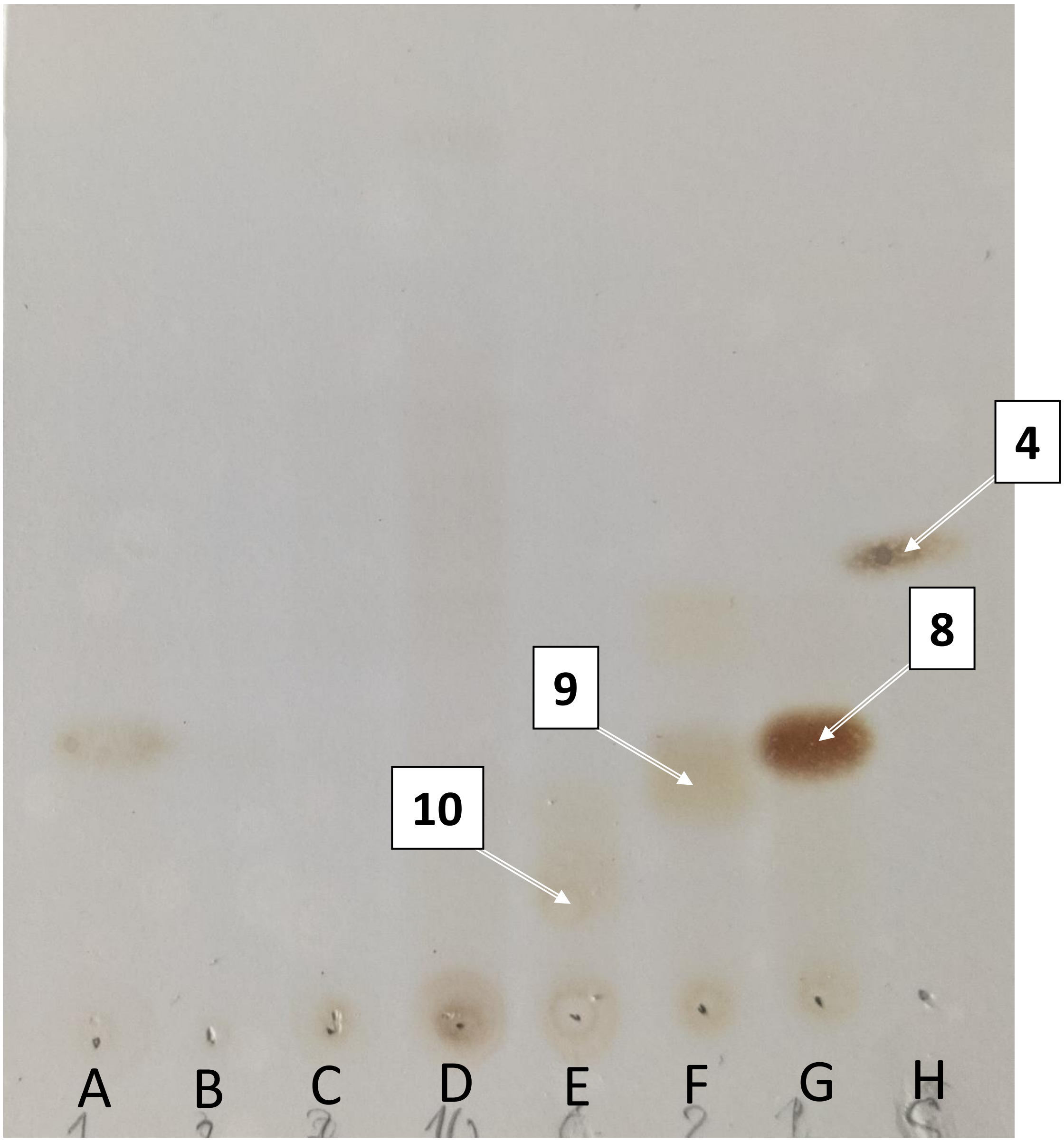

Figure.S40. Thin layer chromatography showing the products of biotransformation 17α-hydroxyprogesterone (**4**): A) one-day transformation; B) three-day transformation; C) seven-day transformation; D) ten-day transformation; E) 6β,12β,17α-trihydroxyprogesterone (**10**); F) 12β,17α-dihydroxyprogesterone (**9**); G) 6β,17α-dihydroxyprogesterone (**8**); H) 17α-hydroxyprogesterone (**4**)

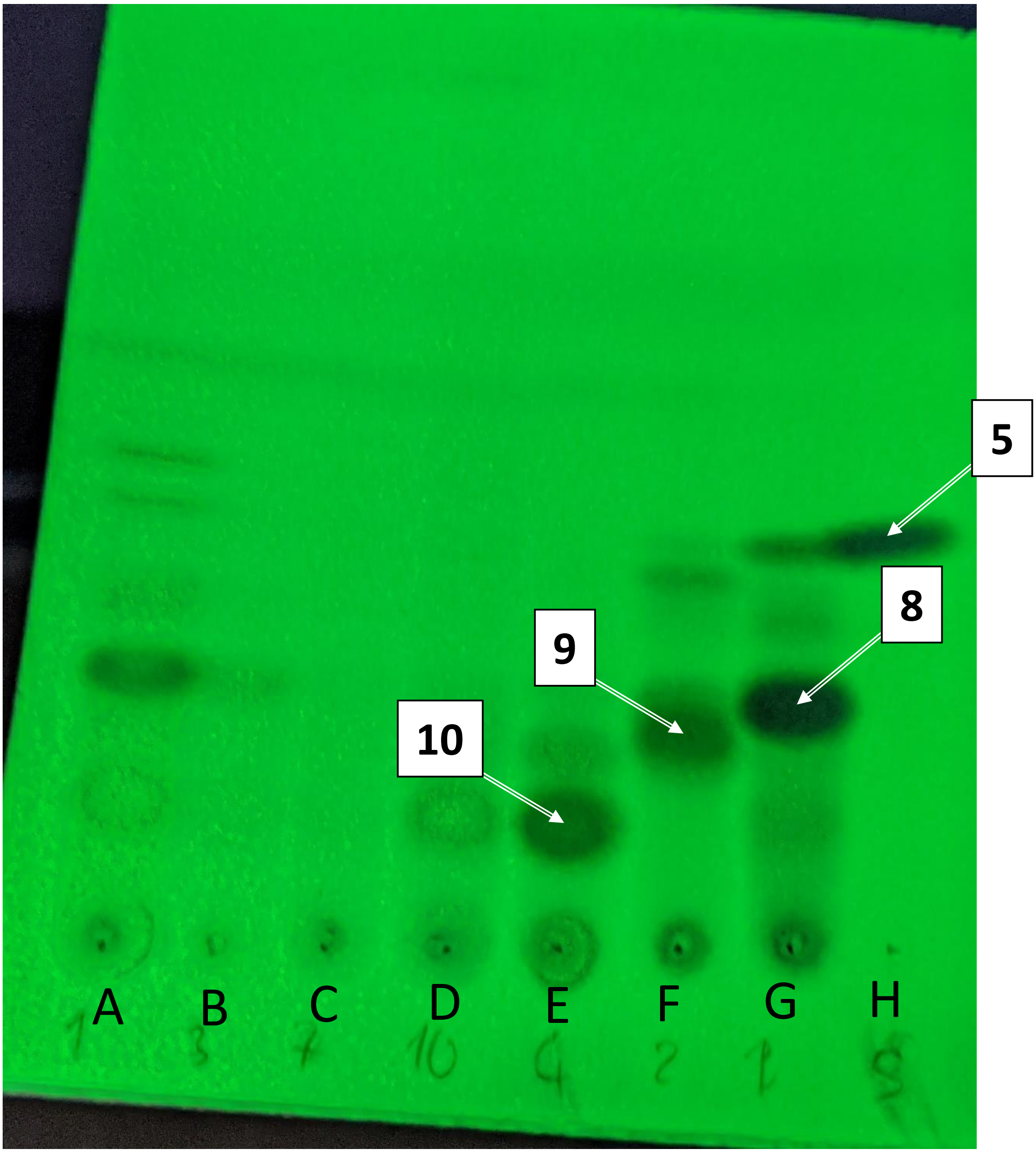

Figure.S41.Thin layer chromatography showing the products of biotransformation 17α-hydroxyprogesterone(**4**):A) one-day transformation;B) three-day transformation; C) seven-day transformation; D) ten-day transformation;E)6β,12β,17α-trihydroxyprogesterone (**10**); F) 12β,17α-dihydroxyprogesterone(**9**); G) 6β,17α-dihydroxyprogesterone(**8**); H) 17α-hydroxyprogesterone(**4**)

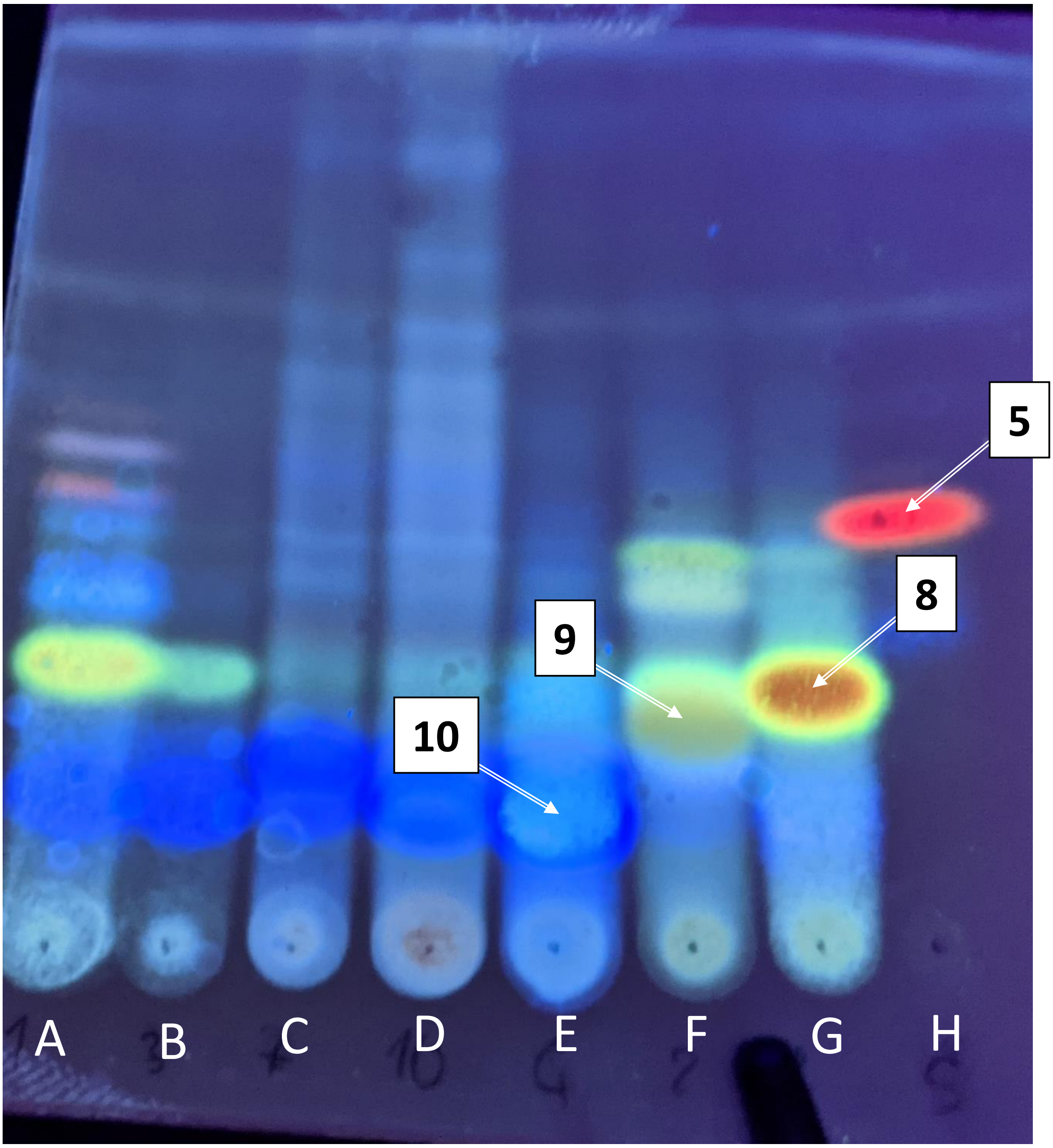

Figure.S42.Thin layer chromatography showing the products of biotransformation 17α-hydroxyprogesterone(**4**):A) one-day transformation;B) three-day transformation; C) seven-day transformation; D) ten-day transformation;E)6β,12β,17α-trihydroxyprogesterone (**10**); F) 12β,17α-dihydroxyprogesterone(**9**); G) 6β,17α-dihydroxyprogesterone(**8**); H)17α-hydroxyprogesterone(**4**)

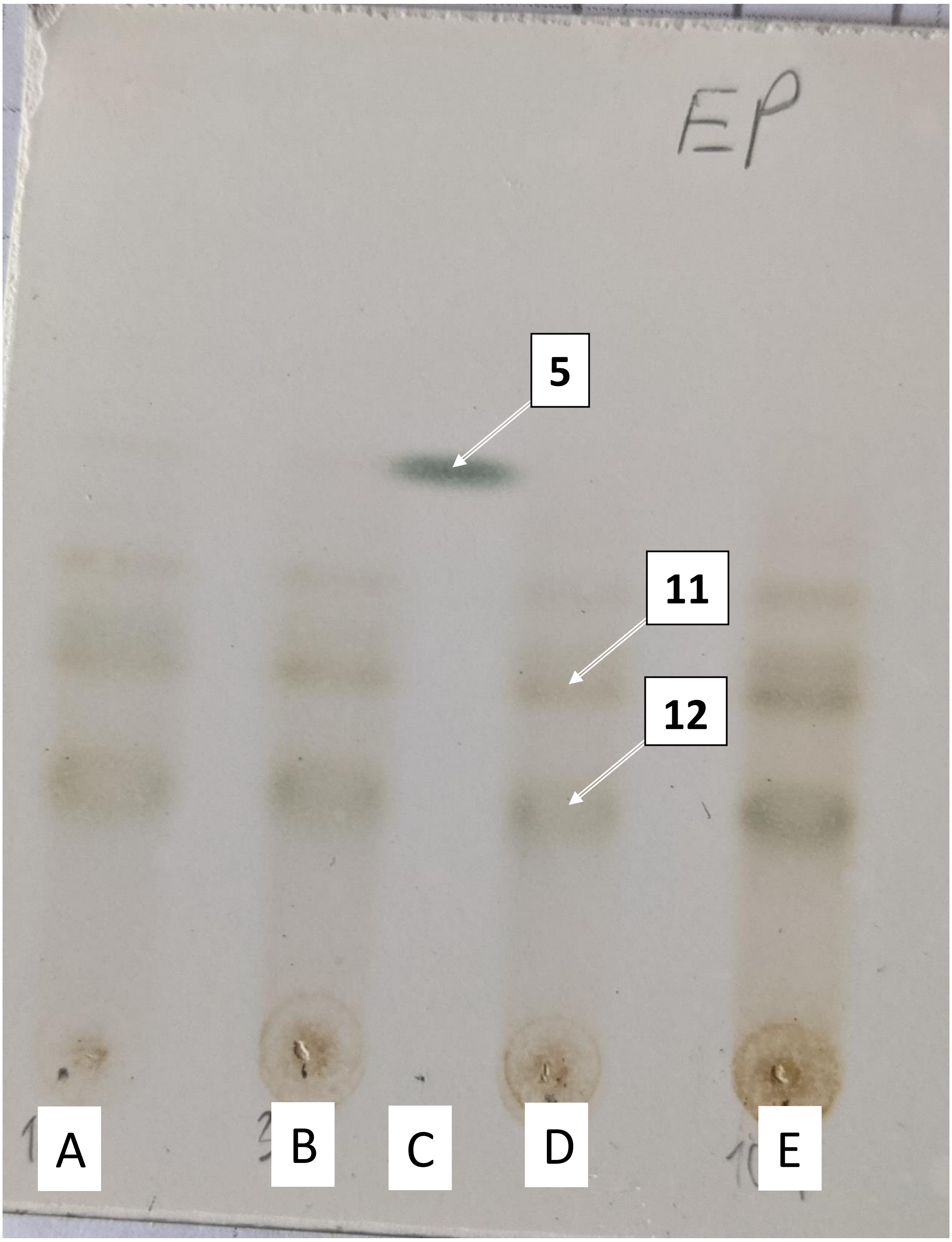

Figure.S43.Thin layer chromatography showing the products of biotransformation 16α,17α-epoxyprogesterone(**5**):A)one-day transformation;B) three-day transformation; C) 16α,17α-epoxyprogesterone(**5**); D) seven-day transformation;E) ten-day transformation

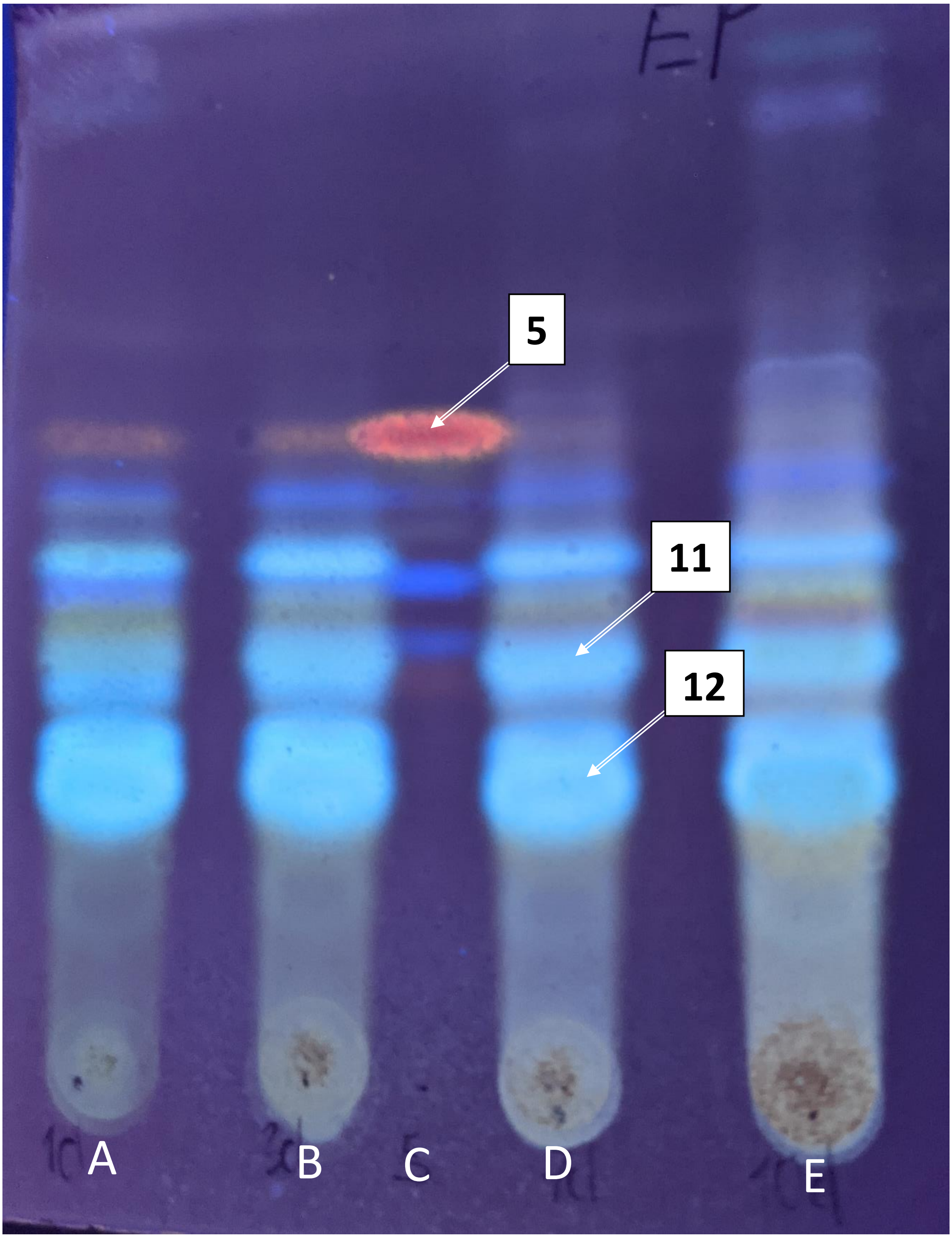

Figure.S44.Thin layer chromatography showing the products of biotransformation 16α,17α-epoxyprogesterone(**5**):A)one-day transformation;B) three-day transformation; C) 16α,17α-epoxyprogesterone(**5**); D)seven-daytransformation;E)ten-day transformation

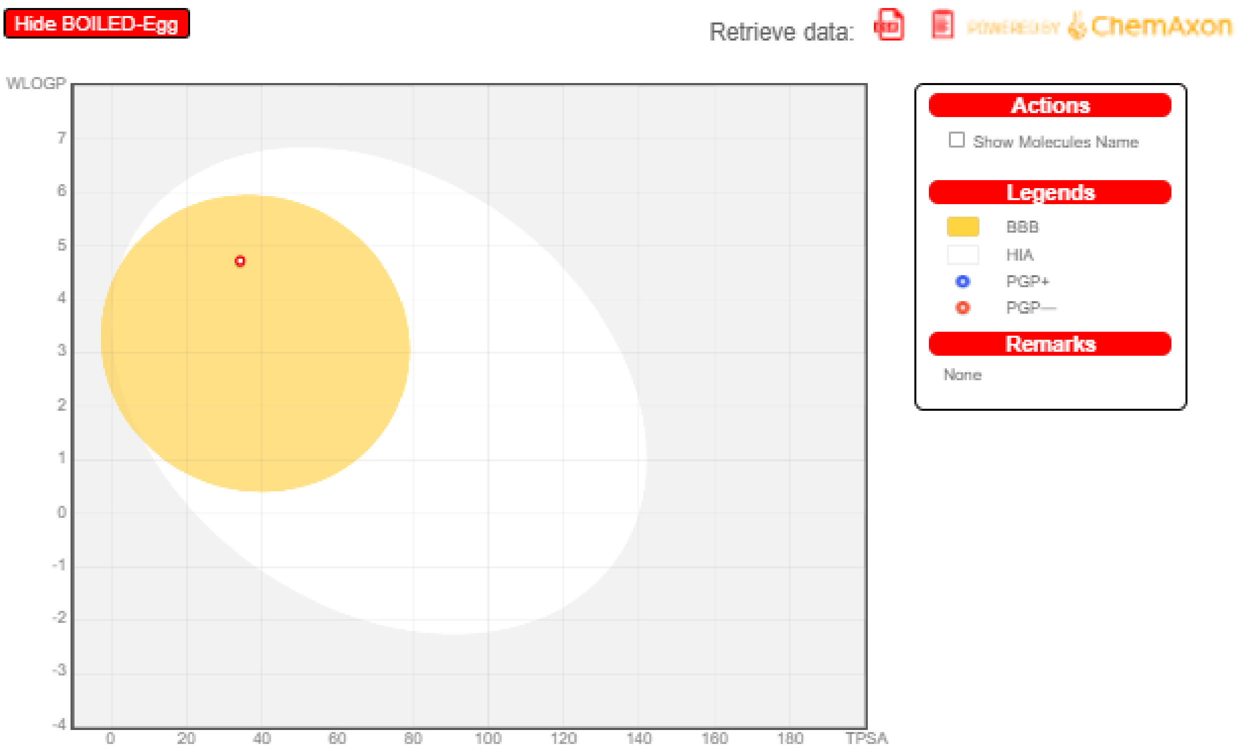

Molecule 1

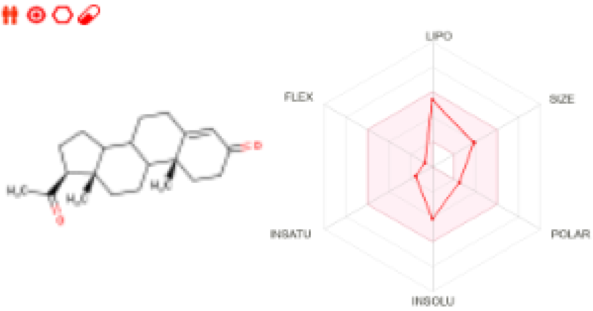

SMILES O=C1CC[C@]2(C(=C1)CCC1C2CC[C@]2(C1CC[C@@H]2C(=O)C)C)C

Physicochemical Properties	
Formula	C21H30O2
Molecular weight	314.46 g/mol
Num. heavy atoms	23
Num. arom. heavy atoms	0
Fraction Csp3	0.81
Num. rotatable bonds	1
Num. H-bond acceptors	2
Num. H-bond donors	0
Molar Refractivity	94.01
TPSA	34.14 Å²

Lipophilicity	
Log $P_{o/w}$ (iLOGP)	3.08
Log $P_{o/w}$ (XLOGP3)	3.87
Log $P_{o/w}$ (WLOGP)	4.72
Log $P_{o/w}$ (MLOGP)	3.95
Log $P_{o/w}$ (SILICOS-IT)	4.49
Consensus Log $P_{o/w}$	4.02

Water Solubility	
Log *S* (ESOL)	-4.16
Solubility	2.17e-02 mg/ml ; 6.89e-05 mol/l
Class	Moderately soluble
Log *S* (Ali)	-4.28
Solubility	1.64e-02 mg/ml ; 5.20e-05 mol/l
Class	Moderately soluble
Log *S* (SILICOS-IT)	-4.38
Solubility	1.30e-02 mg/ml ; 4.15e-05 mol/l
Class	Moderately soluble

Pharmacokinetics	
GI absorption	High
BBB permeant	Yes
P-gp substrate	No
CYP1A2 inhibitor	No
CYP2C19 inhibitor	Yes
CYP2C9 inhibitor	Yes
CYP2D6 inhibitor	No
CYP3A4 inhibitor	No
Log K_p (skin permeation)	-5.47 cm/s

Druglikeness	
Lipinski	Yes; 0 violation
Ghose	Yes
Veber	Yes
Egan	Yes
Muegge	Yes
Bioavailability Score	0.55

Medicinal Chemistry	
PAINS	0 alert
Brenk	0 alert
Leadlikeness	No; 1 violation: XLOGP3>3.5
Synthetic accessibility	4.96

Figure.S45.Progesterone (1) physicochemical and ADME parameters prediction using the SwissADME modelling

Hide BOILED-Egg   Retrieve data:

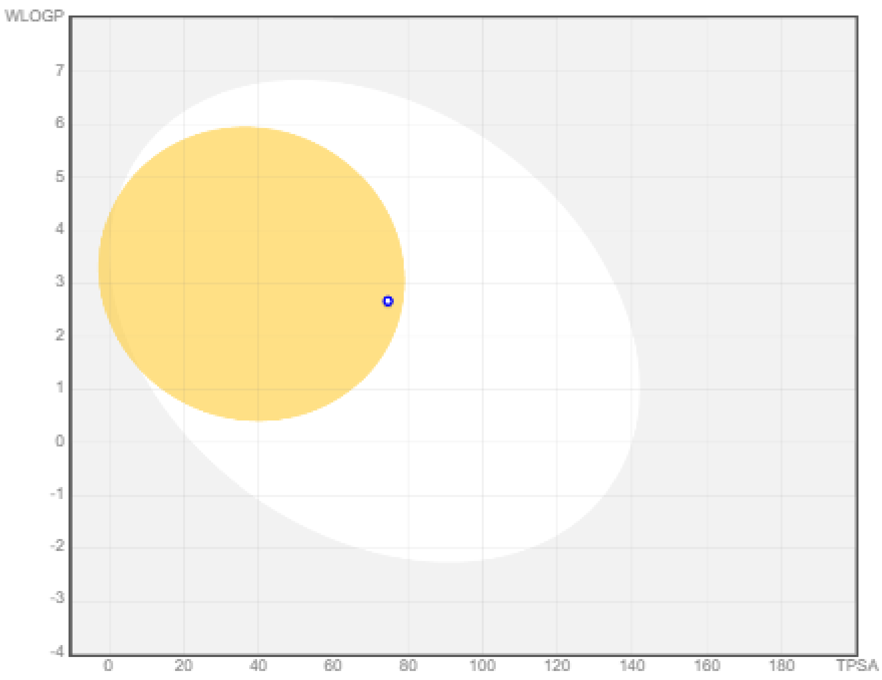

Actions

Show Molecules Name

Legends

BBB

HIA

PGP+

PGP—

Remarks

None

Molecule 1

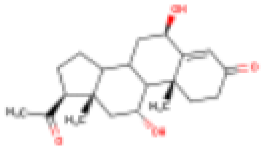

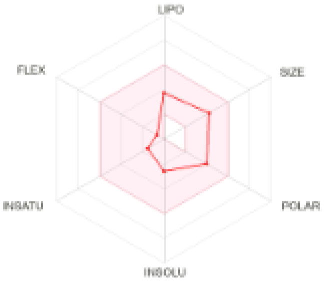

SMILES O=C1CC[C@]2(C(=C1)[C@H](O)CC1C2[C@H](O)C[C@]2(C1CC[C@@H]2C(=O)C)C)C

Physicochemical Properties	
Formula	C21H30O4
Molecular weight	346.46 g/mol
Num. heavy atoms	25
Num. arom. heavy atoms	0
Fraction Csp3	0.81
Num. rotatable bonds	1
Num. H-bond acceptors	4
Num. H-bond donors	2
Molar Refractivity	96.33
TPSA	74.60 Å²

Lipophilicity	
Log $P_{o/w}$ (iLOGP)	2.37
Log $P_{o/w}$ (XLOGP3)	1.06
Log $P_{o/w}$ (WLOGP)	2.67
Log $P_{o/w}$ (MLOGP)	2.22
Log $P_{o/w}$ (SILICOS-IT)	2.69
Consensus Log $P_{o/w}$	2.20

Water Solubility	
Log *S* (ESOL)	-2.59
Solubility	8.91e-01 mg/ml ; 2.57e-03 mol/l
Class	Soluble
Log *S* (Ali)	-2.22
Solubility	2.10e+00 mg/ml ; 6.06e-03 mol/l
Class	Soluble
Log *S* (SILICOS-IT)	-2.75
Solubility	6.12e-01 mg/ml ; 1.77e-03 mol/l
Class	Soluble

Pharmacokinetics	
GI absorption	High
BBB permeant	Yes
P-gp substrate	Yes
CYP1A2 inhibitor	No
CYP2C19 inhibitor	No
CYP2C9 inhibitor	No
CYP2D6 inhibitor	No
CYP3A4 inhibitor	No
Log K_p (skin permeation)	-7.66 cm/s

Druglikeness	
Lipinski	Yes; 0 violation
Ghose	Yes
Veber	Yes
Egan	Yes
Muegge	Yes
Bioavailability Score	0.55

Medicinal Chemistry	
PAINS	0 alert
Brenk	0 alert
Leadlikeness	Yes
Synthetic accessibility	5.19

Figure.S46.6β,11α-Dihydroxyprogesterone (2) physicochemical and ADME parameters prediction usingthe SwissADME modelling

Hide BOILED-Egg

Retrieve data: POWERED BY ChemAxon

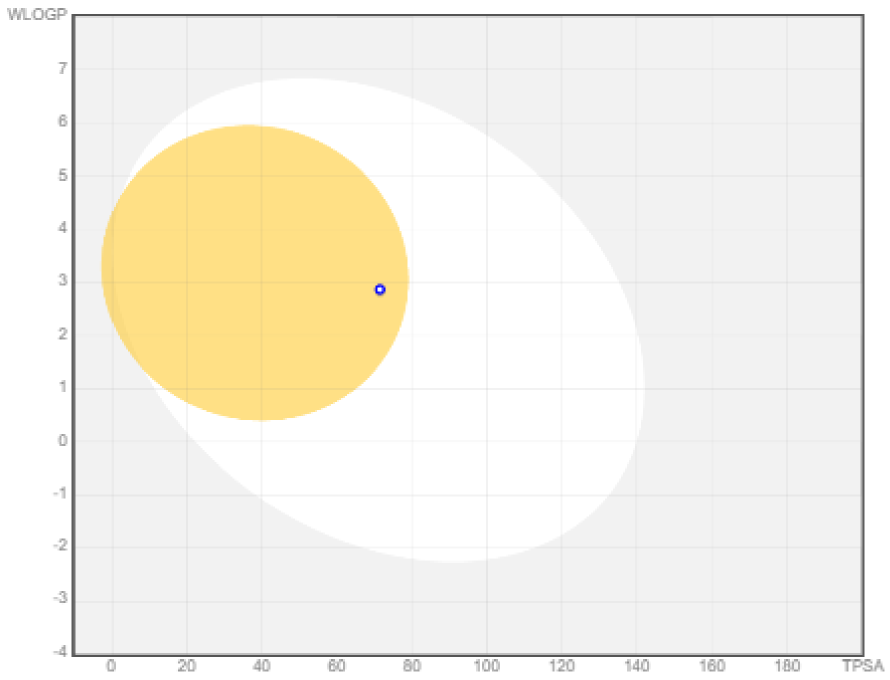

Actions

☐ Show Molecules Name

Legends

- BBB
- HIA
- PGP+
- PGP—

Remarks

None

Molecule 1

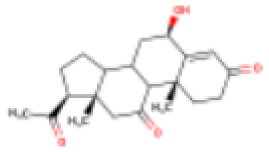

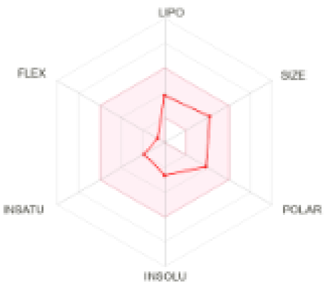

SMILES O=C1CC[C@]2(C(=C1)[C@H](O)CC1C2C(=O)C[C@]2(C1CC[C@@H]2C(=O)C)C)C

Physicochemical Properties	
Formula	C21H28O4
Molecular weight	344.44 g/mol
Num. heavy atoms	25
Num. arom. heavy atoms	0
Fraction Csp3	0.76
Num. rotatable bonds	1
Num. H-bond acceptors	4
Num. H-bond donors	1
Molar Refractivity	95.37
TPSA	71.44 Å²

Lipophilicity	
Log $P_{o/w}$ (iLOGP)	2.18
Log $P_{o/w}$ (XLOGP3)	1.12
Log $P_{o/w}$ (WLOGP)	2.87
Log $P_{o/w}$ (MLOGP)	2.13
Log $P_{o/w}$ (SILICOS-IT)	3.28
Consensus Log $P_{o/w}$	2.32

Water Solubility	
Log *S* (ESOL)	-2.62
Solubility	8.36e-01 mg/ml ; 2.43e-03 mol/l
Class	Soluble
Log *S* (Ali)	-2.21
Solubility	2.11e+00 mg/ml ; 6.11e-03 mol/l
Class	Soluble
Log *S* (SILICOS-IT)	-3.45
Solubility	1.23e-01 mg/ml ; 3.56e-04 mol/l
Class	Soluble

Pharmacokinetics	
GI absorption	High
BBB permeant	Yes
P-gp substrate	Yes
CYP1A2 inhibitor	No
CYP2C19 inhibitor	No
CYP2C9 inhibitor	No
CYP2D6 inhibitor	No
CYP3A4 inhibitor	No
Log K_p (skin permeation)	-7.61 cm/s

Druglikeness	
Lipinski	Yes; 0 violation
Ghose	Yes
Veber	Yes
Egan	Yes
Muegge	Yes
Bioavailability Score	0.55

Medicinal Chemistry	
PAINS	1 alert: steroid_A
Brenk	0 alert
Leadlikeness	Yes
Synthetic accessibility	5.08

Figure.S47.6β-hydroxy-11-oxo-progesterone (7) physicochemical and ADME parameters prediction usingthe SwissADMEmodelling

Hide BOILED-Egg

Retrieve data: POWERED BY ChemAxon

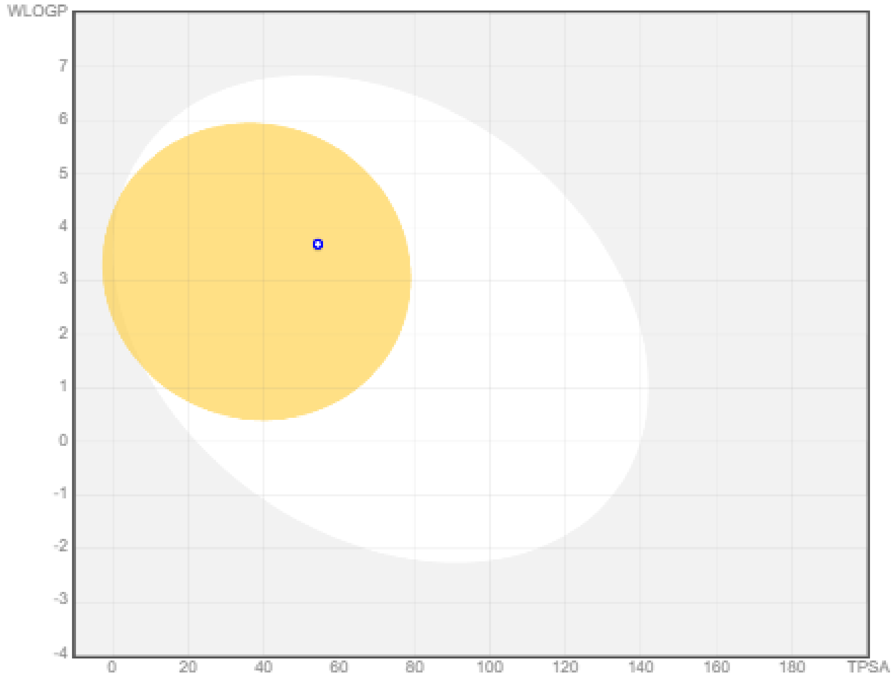

Actions

☐ Show Molecules Name

Legends

BBB
HIA
PGP+
PGP—

Remarks

None

Molecule 1

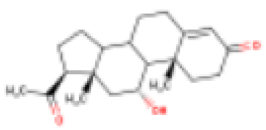

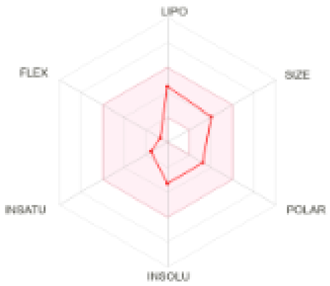

SMILES O=C1CC[C@]2(C(=C1)CCC1C2[C@H](O)C[C@]2(C1CC[C@@H]2C(=O)C)C)C

Physicochemical Properties	
Formula	C21H30O3
Molecular weight	330.46 g/mol
Num. heavy atoms	24
Num. arom. heavy atoms	0
Fraction Csp3	0.81
Num. rotatable bonds	1
Num. H-bond acceptors	3
Num. H-bond donors	1
Molar Refractivity	95.17
TPSA	54.37 Å²

Lipophilicity	
Log $P_{o/w}$ (iLOGP)	2.75
Log $P_{o/w}$ (XLOGP3)	2.36
Log $P_{o/w}$ (WLOGP)	3.69
Log $P_{o/w}$ (MLOGP)	3.07
Log $P_{o/w}$ (SILICOS-IT)	3.59
Consensus Log $P_{o/w}$	3.09

Water Solubility	
Log *S* (ESOL)	-3.31
Solubility	1.62e-01 mg/ml ; 4.90e-04 mol/l
Class	Soluble
Log *S* (Ali)	-3.14
Solubility	2.38e-01 mg/ml ; 7.21e-04 mol/l
Class	Soluble
Log *S* (SILICOS-IT)	-3.57
Solubility	8.94e-02 mg/ml ; 2.70e-04 mol/l
Class	Soluble

Pharmacokinetics	
GI absorption	High
BBB permeant	Yes
P-gp substrate	Yes
CYP1A2 inhibitor	No
CYP2C19 inhibitor	No
CYP2C9 inhibitor	No
CYP2D6 inhibitor	No
CYP3A4 inhibitor	No
Log K_p (skin permeation)	-6.84 cm/s

Druglikeness	
Lipinski	Yes; 0 violation
Ghose	Yes
Veber	Yes
Egan	Yes
Muegge	Yes
Bioavailability Score	0.55

Medicinal Chemistry	
PAINS	0 alert
Brenk	0 alert
Leadlikeness	Yes
Synthetic accessibility	4.96

Figure.S48.11α-Hydroxyprogesterone (3) physicochemical and ADME parameters prediction using the SwissADME modelling

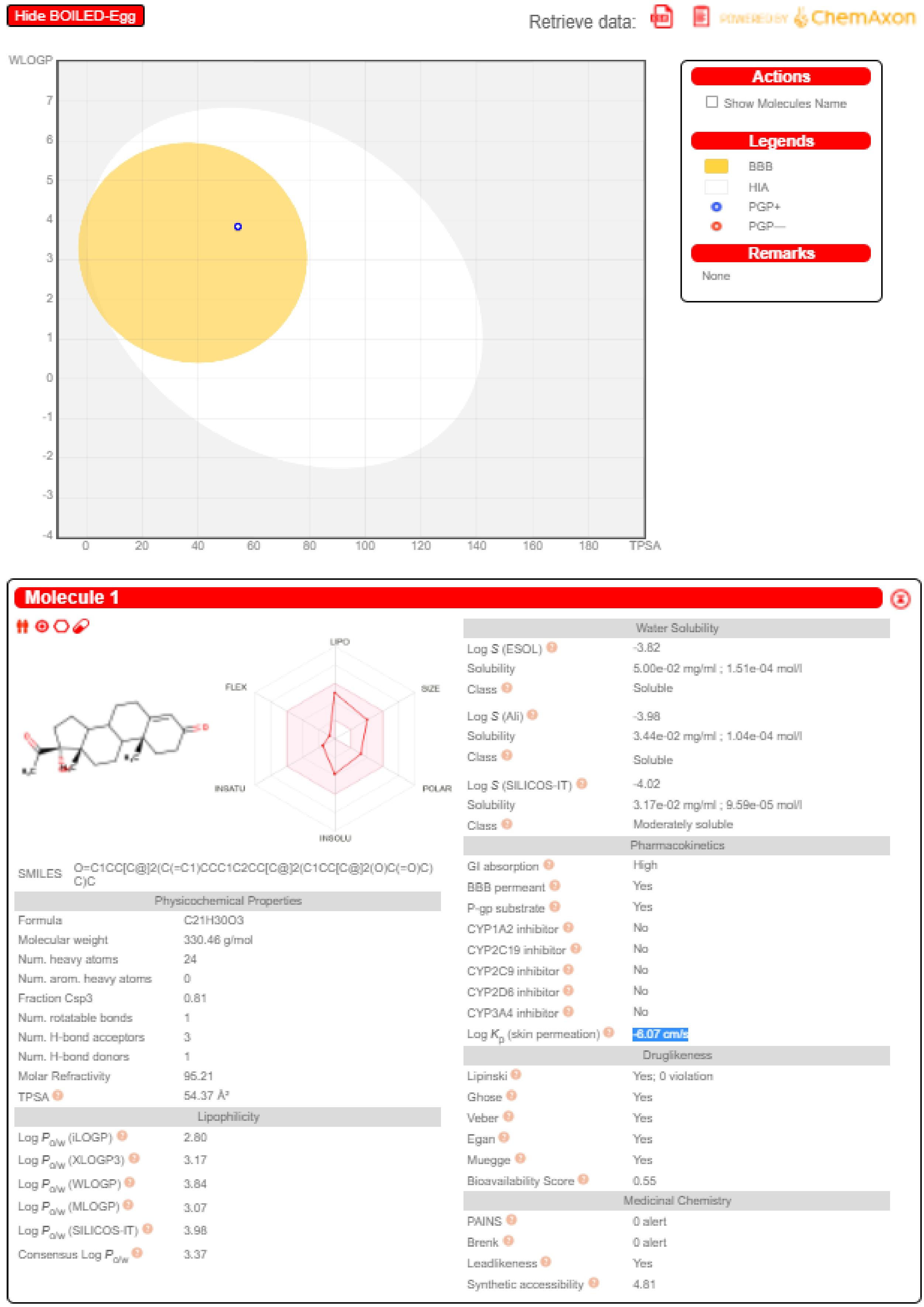

Figure.S49. 17α-Hydroxyprogesterone (**3**) physicochemical and ADME parameters prediction using the SwissADME modelling

Hide BOILED-Egg

Retrieve data: ChemAxon

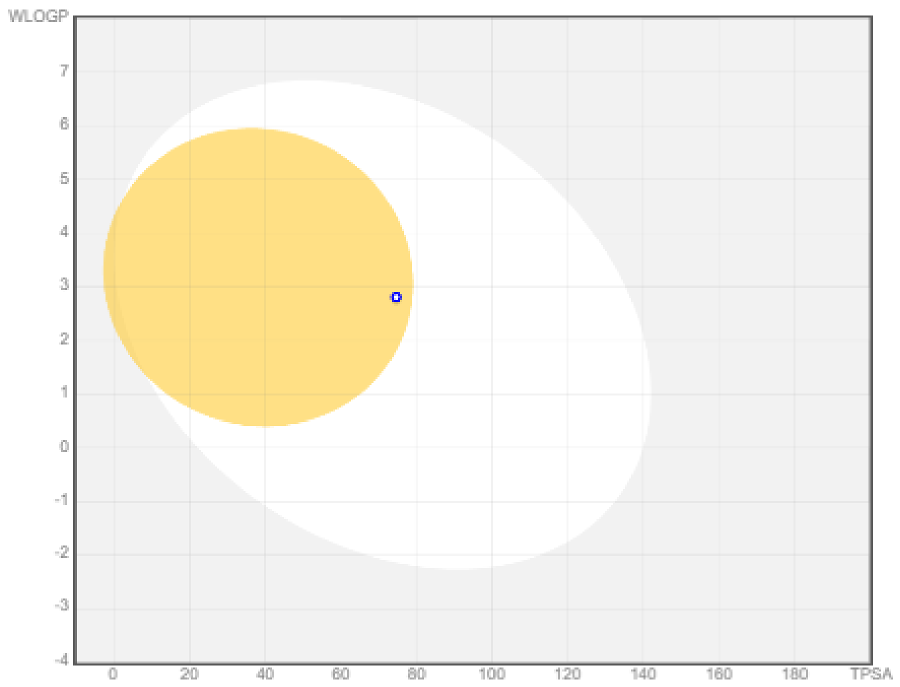

Actions

☐ Show Molecules Name

Legends

BBB
HIA
PGP+
PGP−

Remarks

None

Molecule 1

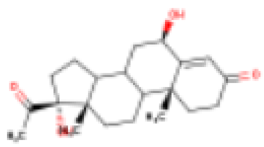

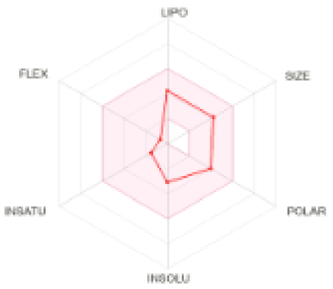

SMILES O=C1CC[C@]2(C(=C1)[C@H](O)CC1C2CC[C@]2(C1CC[C@]2(O)C(=O)C)C)C

Physicochemical Properties	
Formula	C21H30O4
Molecular weight	346.46 g/mol
Num. heavy atoms	25
Num. arom. heavy atoms	0
Fraction Csp3	0.81
Num. rotatable bonds	1
Num. H-bond acceptors	4
Num. H-bond donors	2
Molar Refractivity	96.37
TPSA	74.60 Å²

Lipophilicity	
Log $P_{o/w}$ (iLOGP)	2.54
Log $P_{o/w}$ (XLOGP3)	1.87
Log $P_{o/w}$ (WLOGP)	2.81
Log $P_{o/w}$ (MLOGP)	2.22
Log $P_{o/w}$ (SILICOS-IT)	3.08
Consensus Log $P_{o/w}$	2.50

Water Solubility	
Log *S* (ESOL)	-3.10
Solubility	2.75e-01 mg/ml ; 7.94e-04 mol/l
Class	Soluble
Log *S* (Ali)	-3.06
Solubility	3.03e-01 mg/ml ; 8.74e-04 mol/l
Class	Soluble
Log *S* (SILICOS-IT)	-3.20
Solubility	2.18e-01 mg/ml ; 6.28e-04 mol/l
Class	Soluble

Pharmacokinetics	
GI absorption	High
BBB permeant	Yes
P-gp substrate	Yes
CYP1A2 inhibitor	No
CYP2C19 inhibitor	No
CYP2C9 inhibitor	No
CYP2D6 inhibitor	No
CYP3A4 inhibitor	No
Log K_p (skin permeation)	-7.09 cm/s

Druglikeness	
Lipinski	Yes; 0 violation
Ghose	Yes
Veber	Yes
Egan	Yes
Muegge	Yes
Bioavailability Score	0.55

Medicinal Chemistry	
PAINS	0 alert
Brenk	0 alert
Leadlikeness	Yes
Synthetic accessibility	5.00

Figure.S50. 6β,17α-dihydroxyprogesterone (**8**) physicochemical and ADME parameters prediction using the SwissADME modelling

Hide BOILED-Egg

Retrieve data:

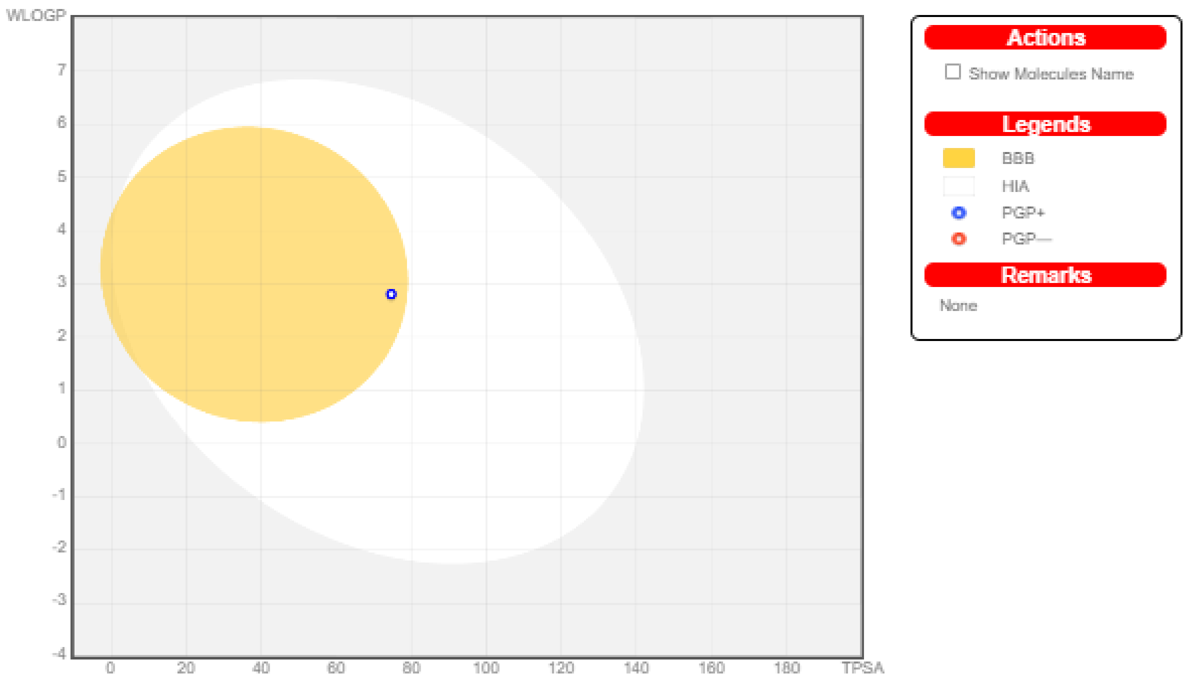

Molecule 1

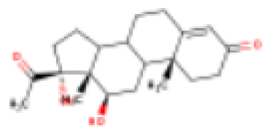

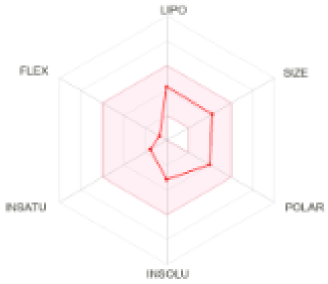

SMILES O=C1CC[C@]2(C(=C1)CCC1C2C[C@@H](O)[C@]2(C1CC[C@]2(O)C(=O)C)C)C

Physicochemical Properties	
Formula	C21H30O4
Molecular weight	346.46 g/mol
Num. heavy atoms	25
Num. arom. heavy atoms	0
Fraction Csp3	0.81
Num. rotatable bonds	1
Num. H-bond acceptors	4
Num. H-bond donors	2
Molar Refractivity	96.37
TPSA	74.60 Å²

Lipophilicity	
Log $P_{o/w}$ (iLOGP)	1.60
Log $P_{o/w}$ (XLOGP3)	2.01
Log $P_{o/w}$ (WLOGP)	2.81
Log $P_{o/w}$ (MLOGP)	2.22
Log $P_{o/w}$ (SILICOS-IT)	3.08
Consensus Log $P_{o/w}$	2.34

Water Solubility	
Log *S* (ESOL)	-3.19
Solubility	2.25e-01 mg/ml ; 6.48e-04 mol/l
Class	Soluble
Log *S* (Ali)	-3.20
Solubility	2.17e-01 mg/ml ; 6.26e-04 mol/l
Class	Soluble
Log *S* (SILICOS-IT)	-3.20
Solubility	2.18e-01 mg/ml ; 6.28e-04 mol/l
Class	Soluble

Pharmacokinetics	
GI absorption	High
BBB permeant	Yes
P-gp substrate	Yes
CYP1A2 inhibitor	No
CYP2C19 inhibitor	No
CYP2C9 inhibitor	No
CYP2D6 inhibitor	No
CYP3A4 inhibitor	No
Log K_p (skin permeation)	-6.99 cm/s

Druglikeness	
Lipinski	Yes; 0 violation
Ghose	Yes
Veber	Yes
Egan	Yes
Muegge	Yes
Bioavailability Score	0.55

Medicinal Chemistry	
PAINS	0 alert
Brenk	0 alert
Leadlikeness	Yes
Synthetic accessibility	5.10

Figure.S51. 12β,17α-dihydroxyprogesterone (**8**) physicochemical and ADME parameters prediction using the SwissADME modelling

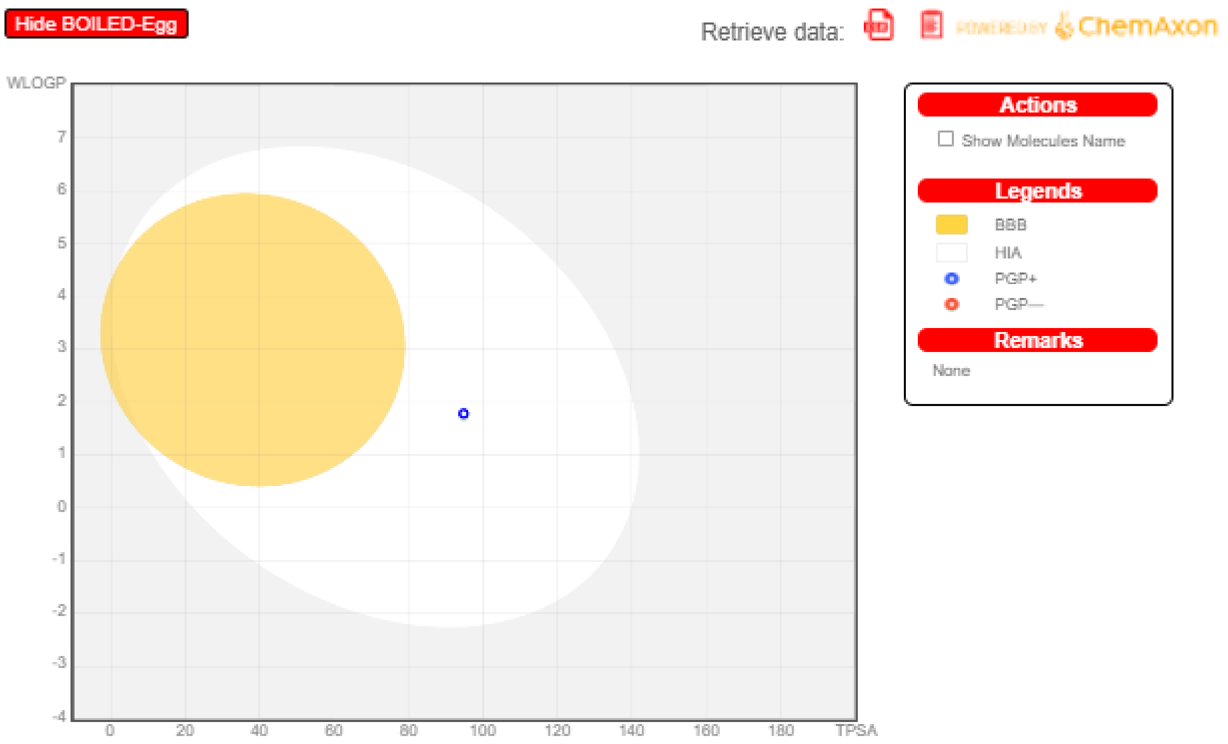

Molecule 1

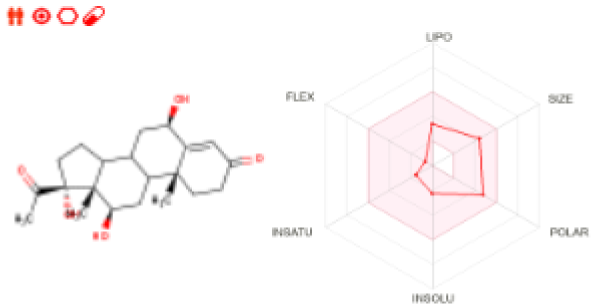

SMILES O=C1CC[C@]2(C(=C1)[C@H](O)CC1C2C[C@@H](O)[C@]2(C1CC[C@]2(O)C(=O)C)C)C

Physicochemical Properties	
Formula	C21H30O5
Molecular weight	362.46 g/mol
Num. heavy atoms	26
Num. arom. heavy atoms	0
Fraction Csp3	0.81
Num. rotatable bonds	1
Num. H-bond acceptors	5
Num. H-bond donors	3
Molar Refractivity	97.53
TPSA	94.83 Å²

Lipophilicity	
Log $P_{o/w}$ (iLOGP)	2.53
Log $P_{o/w}$ (XLOGP3)	0.41
Log $P_{o/w}$ (WLOGP)	1.78
Log $P_{o/w}$ (MLOGP)	1.39
Log $P_{o/w}$ (SILICOS-IT)	2.19
Consensus Log $P_{o/w}$	1.66

Water Solubility	
Log *S* (ESOL)	-2.28
Solubility	1.90e+00 mg/ml ; 5.25e-03 mol/l
Class	Soluble
Log *S* (Ali)	-1.97
Solubility	3.90e+00 mg/ml ; 1.08e-02 mol/l
Class	Very soluble
Log *S* (SILICOS-IT)	-2.39
Solubility	1.49e+00 mg/ml ; 4.11e-03 mol/l
Class	Soluble

Pharmacokinetics	
GI absorption	High
BBB permeant	No
P-gp substrate	Yes
CYP1A2 inhibitor	No
CYP2C19 inhibitor	No
CYP2C9 inhibitor	No
CYP2D6 inhibitor	No
CYP3A4 inhibitor	No
Log K_p (skin permeation)	-8.22 cm/s

Druglikeness	
Lipinski	Yes; 0 violation
Ghose	Yes
Veber	Yes
Egan	Yes
Muegge	Yes
Bioavailability Score	0.55

Medicinal Chemistry	
PAINS	0 alert
Brenk	0 alert
Leadlikeness	No; 1 violation: MW>350
Synthetic accessibility	5.26

Figure.S52. 6β,12β,17α-trihydroxyprogesterone (**10**) physicochemical and ADME parameters prediction using the SwissADME modelling

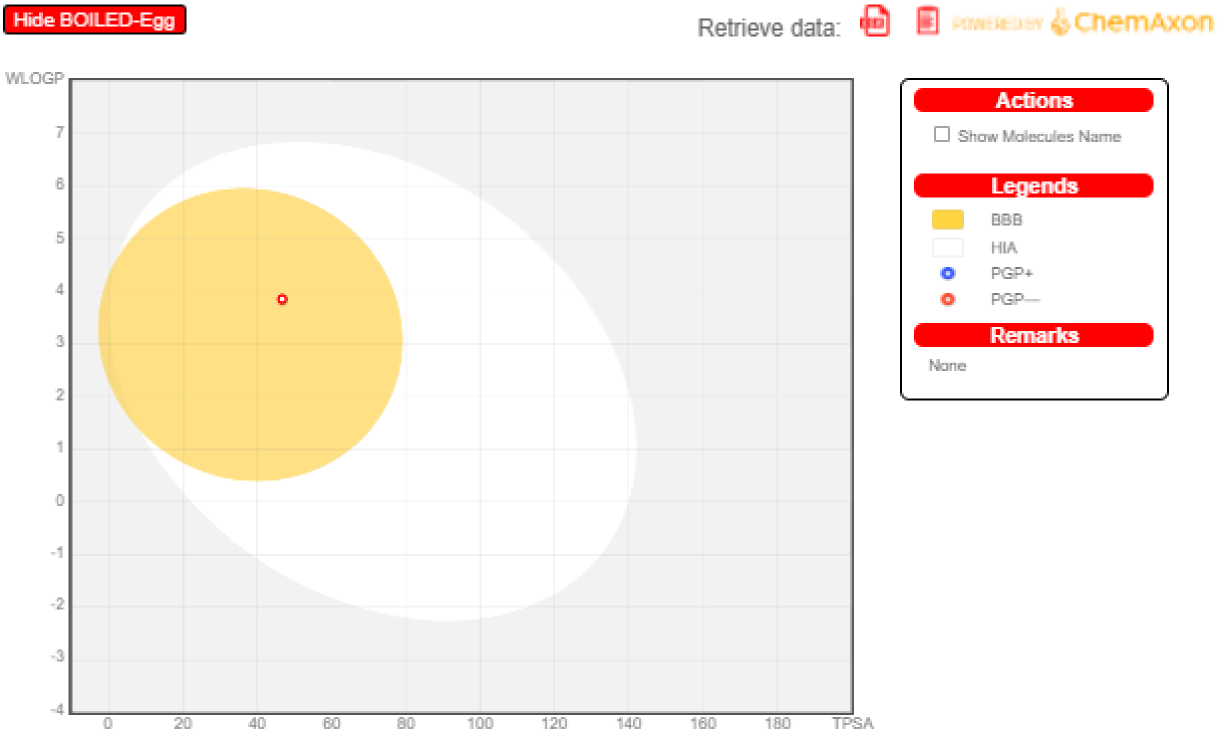

Molecule 1

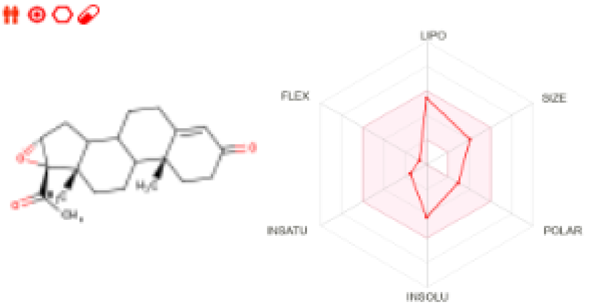

SMILES O=C1CC[C@]2(C(=C1)CCC1C2CC[C@]2(C1C[C@@H]1[C@]2(O1)C(=O)C)C)C

Physicochemical Properties	
Formula	C21H28O3
Molecular weight	328.45 g/mol
Num. heavy atoms	24
Num. arom. heavy atoms	0
Fraction Csp3	0.81
Num. rotatable bonds	1
Num. H-bond acceptors	3
Num. H-bond donors	0
Molar Refractivity	93.02
TPSA	46.67 Å²

Lipophilicity	
Log $P_{o/w}$ (iLOGP)	3.11
Log $P_{o/w}$ (XLOGP3)	3.93
Log $P_{o/w}$ (WLOGP)	3.85
Log $P_{o/w}$ (MLOGP)	3.07
Log $P_{o/w}$ (SILICOS-IT)	4.33
Consensus Log $P_{o/w}$	3.66

Water Solubility	
Log *S* (ESOL)	-4.29
Solubility	1.70e-02 mg/ml ; 5.17e-05 mol/l
Class	Moderately soluble
Log *S* (Ali)	-4.61
Solubility	8.07e-03 mg/ml ; 2.46e-05 mol/l
Class	Moderately soluble
Log *S* (SILICOS-IT)	-4.10
Solubility	2.63e-02 mg/ml ; 8.00e-05 mol/l
Class	Moderately soluble

Pharmacokinetics	
GI absorption	High
BBB permeant	Yes
P-gp substrate	No
CYP1A2 inhibitor	No
CYP2C19 inhibitor	No
CYP2C9 inhibitor	Yes
CYP2D6 inhibitor	No
CYP3A4 inhibitor	No
Log K_p (skin permeation)	-5.51 cm/s

Druglikeness	
Lipinski	Yes; 0 violation
Ghose	Yes
Veber	Yes
Egan	Yes
Muegge	Yes
Bioavailability Score	0.55

Medicinal Chemistry	
PAINS	0 alert
Brenk	1 alert: Three-membered_heterocycle
Leadlikeness	No; 1 violation: XLOGP3>3.5
Synthetic accessibility	4.91

Figure.S53. 16α,17α-epoxyprogesterone (**5**) physicochemical and ADME parameters prediction using the SwissADME modelling

Hide BOILED-Egg

Retrieve data: POWERED BY ChemAxon

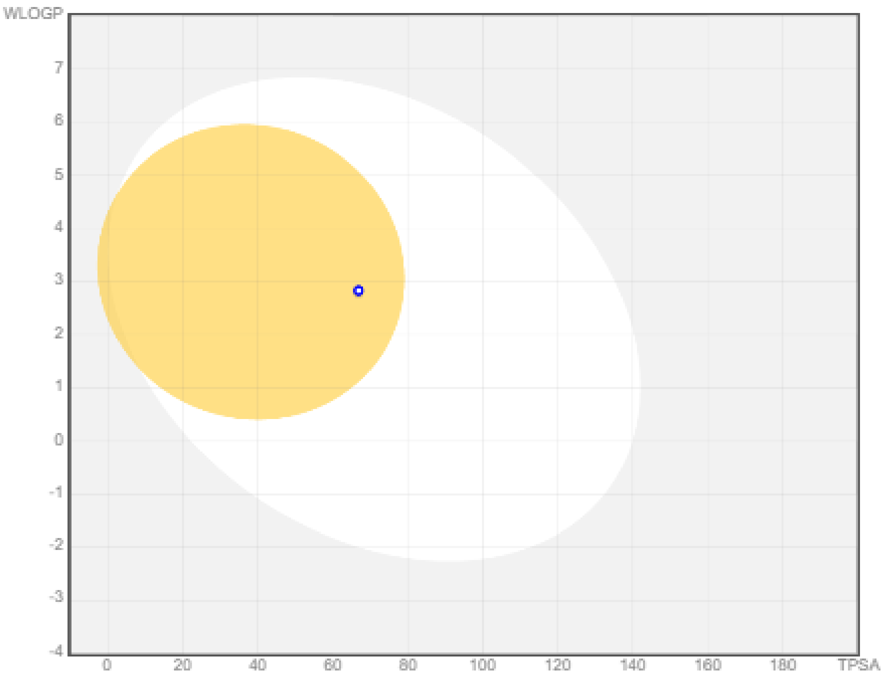

Actions

Show Molecules Name

Legends

BBB

HIA

PGP+

PGP—

Remarks

None

Molecule 1

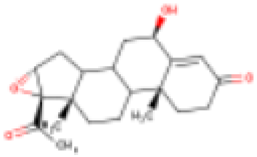

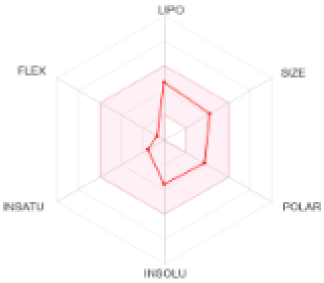

SMILES O=C1CC[C@]2(C(=C1)[C@H](O)CC1C2CC[C@]2(C1C[C@@H]1[C@]2(O1)C(=O)C)C)C

Physicochemical Properties	
Formula	C21H28O4
Molecular weight	344.44 g/mol
Num. heavy atoms	25
Num. arom. heavy atoms	0
Fraction Csp3	0.81
Num. rotatable bonds	1
Num. H-bond acceptors	4
Num. H-bond donors	1
Molar Refractivity	94.18
TPSA	66.90 Å²

Lipophilicity	
Log $P_{o/w}$ (iLOGP)	2.81
Log $P_{o/w}$ (XLOGP3)	2.64
Log $P_{o/w}$ (WLOGP)	2.83
Log $P_{o/w}$ (MLOGP)	2.22
Log $P_{o/w}$ (SILICOS-IT)	3.43
Consensus Log $P_{o/w}$	2.78

Water Solubility	
Log *S* (ESOL)	-3.57
Solubility	9.21e-02 mg/ml ; 2.67e-04 mol/l
Class	Soluble
Log *S* (Ali)	-3.70
Solubility	6.94e-02 mg/ml ; 2.02e-04 mol/l
Class	Soluble
Log *S* (SILICOS-IT)	-3.28
Solubility	1.80e-01 mg/ml ; 5.24e-04 mol/l
Class	Soluble

Pharmacokinetics	
GI absorption	High
BBB permeant	Yes
P-gp substrate	Yes
CYP1A2 inhibitor	No
CYP2C19 inhibitor	No
CYP2C9 inhibitor	No
CYP2D6 inhibitor	No
CYP3A4 inhibitor	No
Log K_p (skin permeation)	-6.53 cm/s

Druglikeness	
Lipinski	Yes; 0 violation
Ghose	Yes
Veber	Yes
Egan	Yes
Muegge	Yes
Bioavailability Score	0.55

Medicinal Chemistry	
PAINS	0 alert
Brenk	1 alert: Three-membered_heterocycle
Leadlikeness	Yes
Synthetic accessibility	5.07

Figure.S54. 6β-hydroxy-16α,17α-epoxyprogesterone (**11**) physicochemical and ADME parameters prediction using the SwissADME modelling

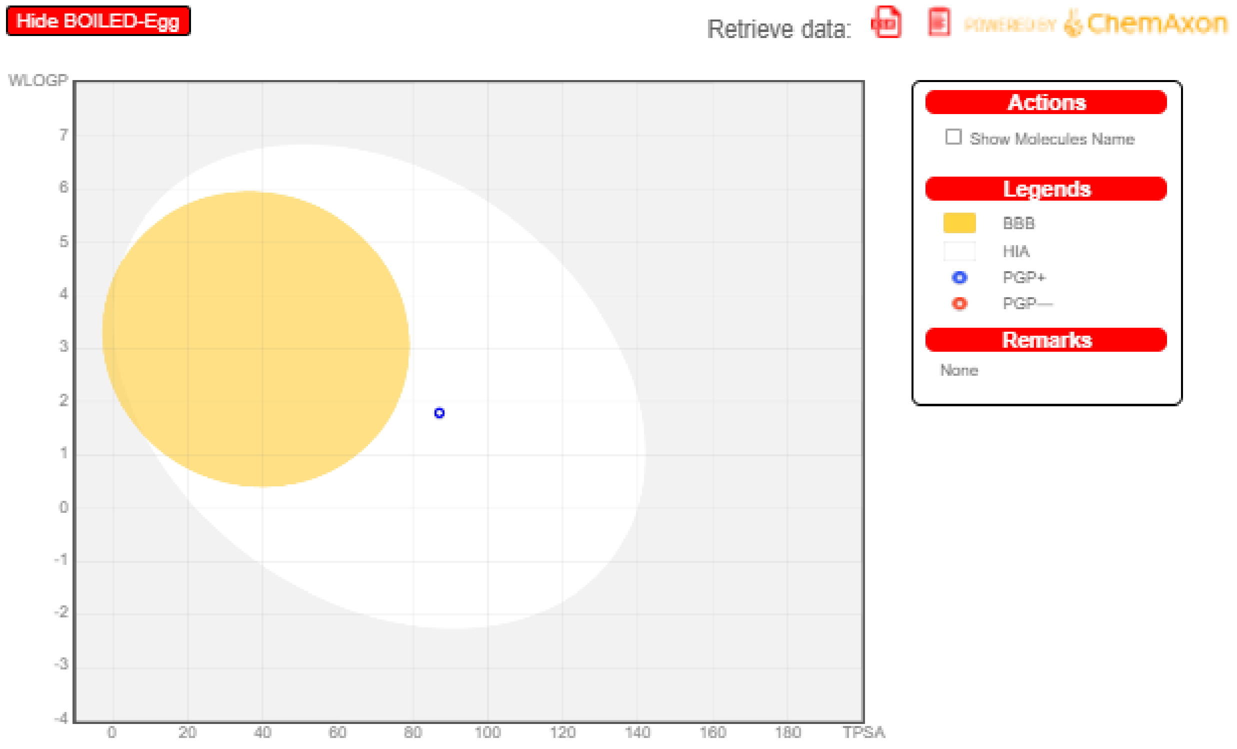

Molecule 1

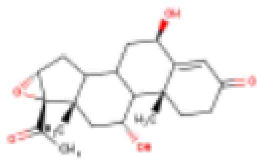

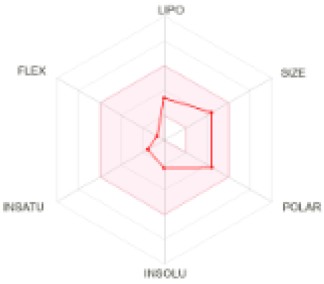

SMILES O=C1CC[C@]2(C(=C1)[C@H](O)CC1C2[C@H](O)C[C@]2(C1C[C@@H]1[C@]2(O1)C(=O)C)C)C

Physicochemical Properties	
Formula	C21H28O5
Molecular weight	360.44 g/mol
Num. heavy atoms	26
Num. arom. heavy atoms	0
Fraction Csp3	0.81
Num. rotatable bonds	1
Num. H-bond acceptors	5
Num. H-bond donors	2
Molar Refractivity	95.34
TPSA	87.13 Å²

Lipophilicity	
Log $P_{o/w}$ (iLOGP)	2.34
Log $P_{o/w}$ (XLOGP3)	0.43
Log $P_{o/w}$ (WLOGP)	1.80
Log $P_{o/w}$ (MLOGP)	1.39
Log $P_{o/w}$ (SILICOS-IT)	2.53
Consensus Log $P_{o/w}$	1.70

Water Solubility	
Log *S* (ESOL)	-2.28
Solubility	1.89e+00 mg/ml ; 5.25e-03 mol/l
Class	Soluble
Log *S* (Ali)	-1.83
Solubility	5.37e+00 mg/ml ; 1.49e-02 mol/l
Class	Very soluble
Log *S* (SILICOS-IT)	-2.46
Solubility	1.24e+00 mg/ml ; 3.43e-03 mol/l
Class	Soluble

Pharmacokinetics	
GI absorption	High
BBB permeant	No
P-gp substrate	Yes
CYP1A2 inhibitor	No
CYP2C19 inhibitor	No
CYP2C9 inhibitor	No
CYP2D6 inhibitor	No
CYP3A4 inhibitor	No
Log K_p (skin permeation)	-8.19 cm/s

Druglikeness	
Lipinski	Yes; 0 violation
Ghose	Yes
Veber	Yes
Egan	Yes
Muegge	Yes
Bioavailability Score	0.55

Medicinal Chemistry	
PAINS	0 alert
Brenk	1 alert: Three-membered_heterocycle
Leadlikeness	No; 1 violation: MW>350
Synthetic accessibility	5.34

Figure.S55. 6β,11α-dihydroxy-16α,17α-epoxyprogesterone (**12**) physicochemical and ADME parameters prediction using the SwissADME modelling

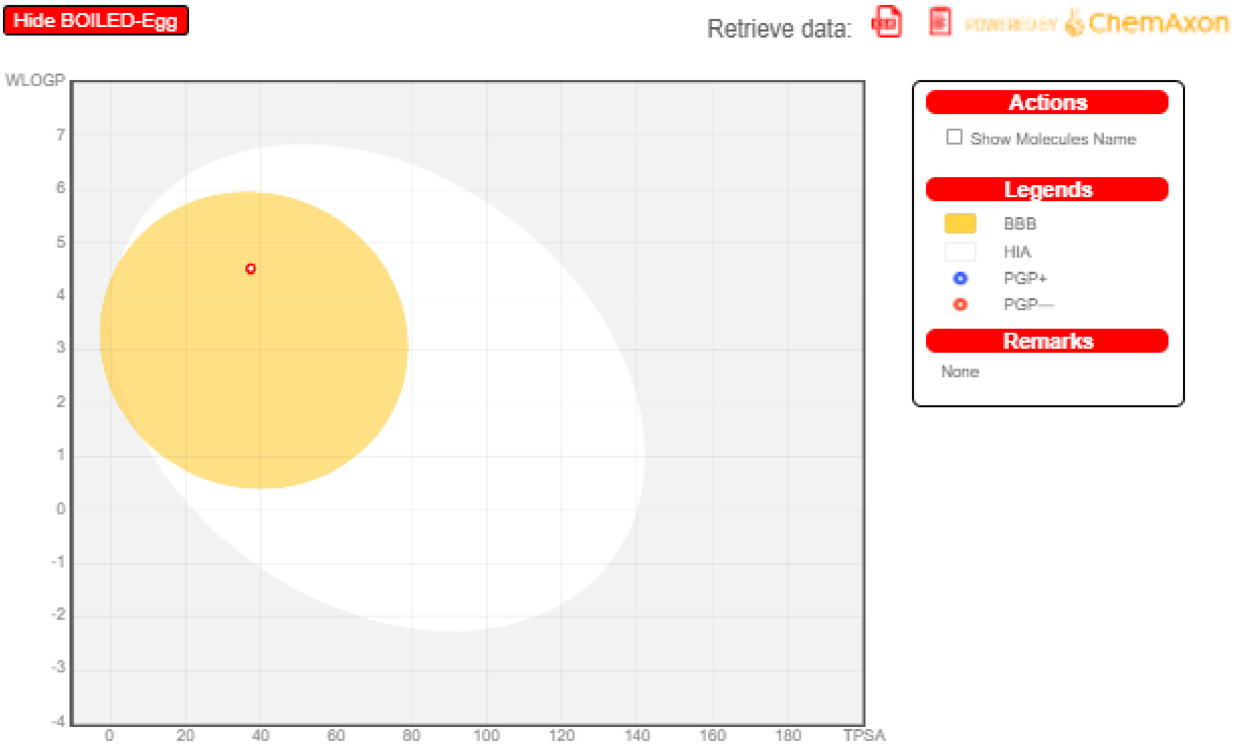

Molecule 1

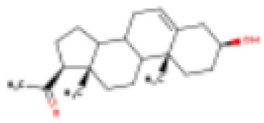

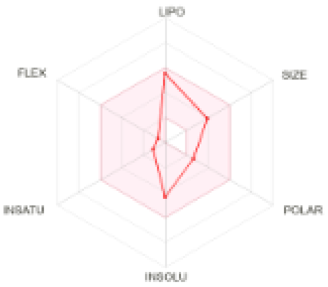

SMILES O[C@H]1CC[C@]2(C(=CCC3C2CC[C@]2(C3CC[C@@H]2C(=O)C)C)C1)C

Physicochemical Properties	
Formula	C21H32O2
Molecular weight	316.48 g/mol
Num. heavy atoms	23
Num. arom. heavy atoms	0
Fraction Csp3	0.86
Num. rotatable bonds	1
Num. H-bond acceptors	2
Num. H-bond donors	1
Molar Refractivity	94.97
TPSA	37.30 Å²

Lipophilicity	
Log $P_{o/w}$ (iLOGP)	3.15
Log $P_{o/w}$ (XLOGP3)	4.22
Log $P_{o/w}$ (WLOGP)	4.52
Log $P_{o/w}$ (MLOGP)	4.05
Log $P_{o/w}$ (SILICOS-IT)	3.90
Consensus Log $P_{o/w}$	3.97

Water Solubility	
Log *S* (ESOL)	-4.39
Solubility	1.28e-02 mg/ml ; 4.03e-05 mol/l
Class	Moderately soluble
Log *S* (Ali)	-4.71
Solubility	6.12e-03 mg/ml ; 1.93e-05 mol/l
Class	Moderately soluble
Log *S* (SILICOS-IT)	-3.69
Solubility	6.51e-02 mg/ml ; 2.06e-04 mol/l
Class	Soluble

Pharmacokinetics	
GI absorption	High
BBB permeant	Yes
P-gp substrate	No
CYP1A2 inhibitor	No
CYP2C19 inhibitor	No
CYP2C9 inhibitor	Yes
CYP2D6 inhibitor	No
CYP3A4 inhibitor	No
Log K_p (skin permeation)	-5.23 cm/s

Druglikeness	
Lipinski	Yes; 0 violation
Ghose	Yes
Veber	Yes
Egan	Yes
Muegge	Yes
Bioavailability Score	0.55

Medicinal Chemistry	
PAINS	0 alert
Brenk	1 alert: isolated_alkene
Leadlikeness	No; 1 violation: XLOGP3>3.5
Synthetic accessibility	4.97

Figure.S56. Pregnenolone (**6**) physicochemical and ADME parameters prediction using the SwissADME modelling

Hide BOILED-Egg

Retrieve data:

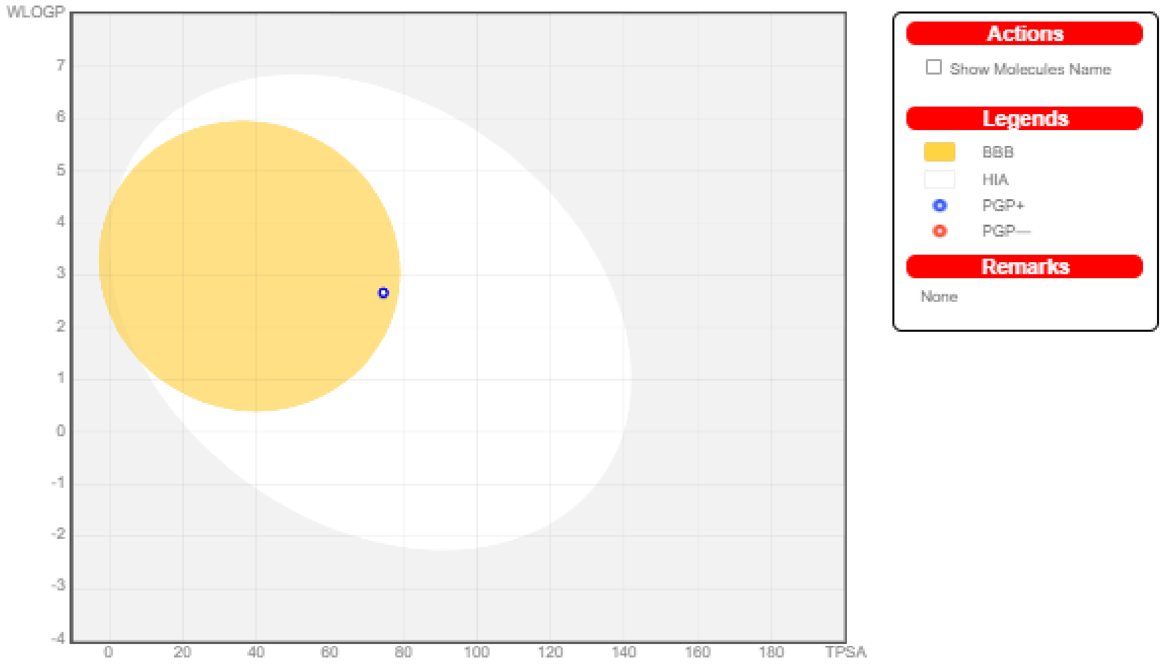

Molecule 1

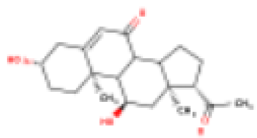

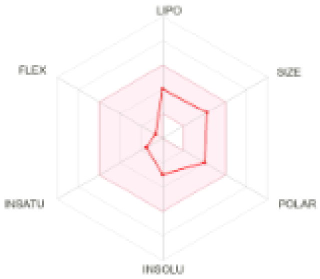

SMILES O[C@H]1CC[C@]2(C(=CC(=O)C3C2[C@H](O)C[C@]2(C3CC[C@@H]2C(=O)C)C)C1)C

Physicochemical Properties	
Formula	C21H30O4
Molecular weight	346.46 g/mol
Num. heavy atoms	25
Num. arom. heavy atoms	0
Fraction Csp3	0.81
Num. rotatable bonds	1
Num. H-bond acceptors	4
Num. H-bond donors	2
Molar Refractivity	96.33
TPSA	74.60 Å²

Lipophilicity	
Log $P_{o/w}$ (iLOGP)	2.45
Log $P_{o/w}$ (XLOGP3)	1.63
Log $P_{o/w}$ (WLOGP)	2.67
Log $P_{o/w}$ (MLOGP)	2.22
Log $P_{o/w}$ (SILICOS-IT)	2.69
Consensus Log $P_{o/w}$	2.33

Water Solubility	
Log *S* (ESOL)	-2.95
Solubility	3.90e-01 mg/ml ; 1.12e-03 mol/l
Class	Soluble
Log *S* (Ali)	-2.81
Solubility	5.38e-01 mg/ml ; 1.55e-03 mol/l
Class	Soluble
Log *S* (SILICOS-IT)	-2.75
Solubility	6.12e-01 mg/ml ; 1.77e-03 mol/l
Class	Soluble

Pharmacokinetics	
GI absorption	High
BBB permeant	Yes
P-gp substrate	Yes
CYP1A2 inhibitor	No
CYP2C19 inhibitor	No
CYP2C9 inhibitor	No
CYP2D6 inhibitor	No
CYP3A4 inhibitor	No
Log K_p (skin permeation)	-7.26 cm/s

Druglikeness	
Lipinski	Yes; 0 violation
Ghose	Yes
Veber	Yes
Egan	Yes
Muegge	Yes
Bioavailability Score	0.55

Medicinal Chemistry	
PAINS	0 alert
Brenk	0 alert
Leadlikeness	Yes
Synthetic accessibility	5.30

Figure.S57. 11α-Hydroxy-7-oxopregnenolone (**13**) physicochemical and ADME parameters prediction using the SwissADME modelling

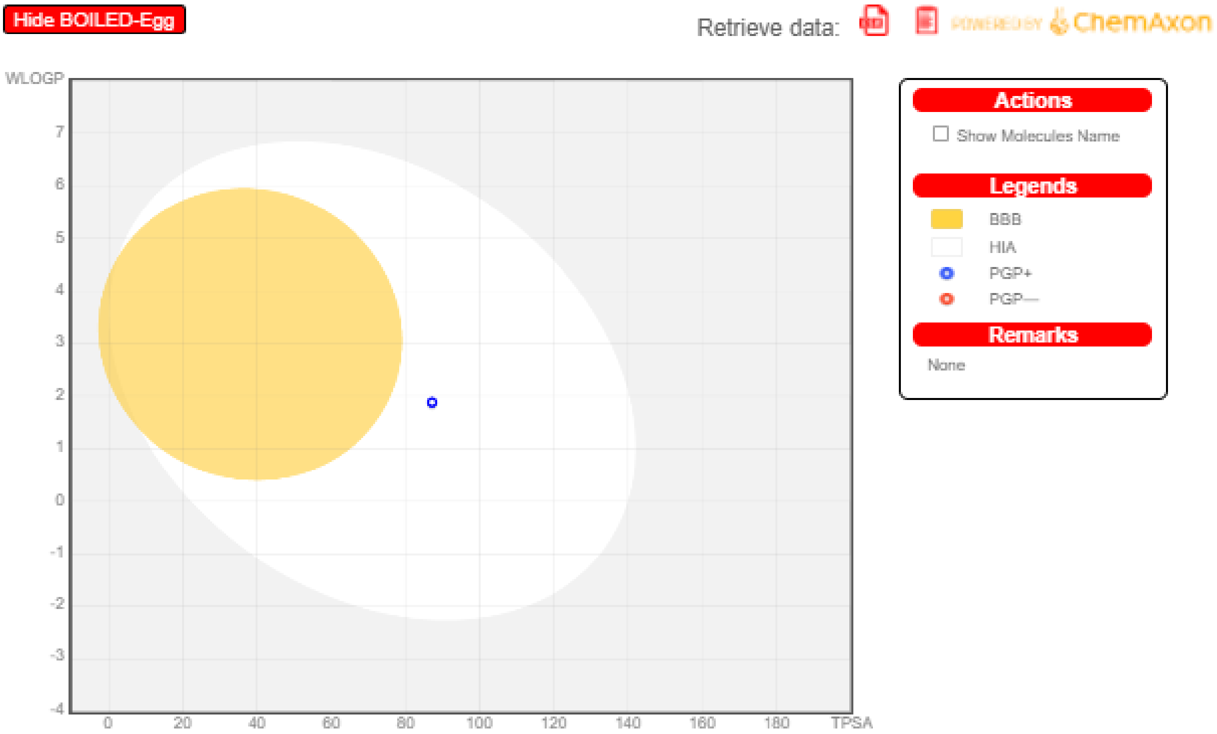

Molecule 1

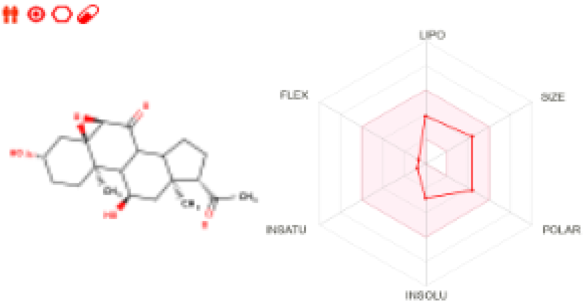

SMILES O[C@H]1CC[C@]2([C@@]3(C1)O[C@H]3C(=O)C1C2[C@H](O)C[C@]2(C1CC[C@@H]2C(=O)C)C)C

Physicochemical Properties	
Formula	C21H30O5
Molecular weight	362.46 g/mol
Num. heavy atoms	26
Num. arom. heavy atoms	0
Fraction Csp3	0.90
Num. rotatable bonds	1
Num. H-bond acceptors	5
Num. H-bond donors	2
Molar Refractivity	95.82
TPSA	87.13 Å²

Lipophilicity	
Log $P_{o/w}$ (iLOGP)	2.32
Log $P_{o/w}$ (XLOGP3)	1.28
Log $P_{o/w}$ (WLOGP)	1.88
Log $P_{o/w}$ (MLOGP)	1.49
Log $P_{o/w}$ (SILICOS-IT)	2.54
Consensus Log $P_{o/w}$	1.90

Water Solubility	
Log *S* (ESOL)	-2.83
Solubility	5.39e-01 mg/ml ; 1.49e-03 mol/l
Class	Soluble
Log *S* (Ali)	-2.71
Solubility	7.08e-01 mg/ml ; 1.95e-03 mol/l
Class	Soluble
Log *S* (SILICOS-IT)	-2.48
Solubility	1.19e+00 mg/ml ; 3.30e-03 mol/l
Class	Soluble

Pharmacokinetics	
GI absorption	High
BBB permeant	No
P-gp substrate	Yes
CYP1A2 inhibitor	No
CYP2C19 inhibitor	No
CYP2C9 inhibitor	No
CYP2D6 inhibitor	No
CYP3A4 inhibitor	No
Log K_p (skin permeation)	-7.60 cm/s

Druglikeness	
Lipinski	Yes; 0 violation
Ghose	Yes
Veber	Yes
Egan	Yes
Muegge	Yes
Bioavailability Score	0.55

Medicinal Chemistry	
PAINS	0 alert
Brenk	1 alert: Three-membered_heterocycle
Leadlikeness	No; 1 violation: MW>350
Synthetic accessibility	5.70

Figure.S58. 5α,6α-epoxy-3β,11α-dihydroxypregnan-7,20-dione (**14**) physicochemical and ADME parameters prediction using the SwissADME modelling
